# Modelling knowledge, health beliefs, and health-promoting behaviours related to cardiovascular disease prevention among Malaysian university students

**DOI:** 10.1371/journal.pone.0250627

**Published:** 2021-04-28

**Authors:** Bee Chiu Lim, Yee Cheng Kueh, Wan Nor Arifin, Kok Huan Ng

**Affiliations:** 1 Clinical Research Centre Hospital Tengku Ampuan Afzan, Ministry of Health Malaysia, Kuantan, Pahang, Malaysia; 2 Biostatistics and Research Methodology Unit, School of Medical Sciences, Universiti Sains Malaysia, Kubang Kerian, Kelantan, Malaysia; 3 KPJ Pahang Specialist Hospital, Kuantan, Pahang, Malaysia; University of Lincoln-UK, UNITED KINGDOM

## Abstract

**Background:**

Healthy lifestyle habits formed during young adulthood may have a sustaining impact on health across later life. The current study aimed to test the theoretical model of factors (selected demographic variables, knowledge of heart disease, health belief related to cardiovascular disease (CVD), self-efficacy, cues to action, and screening intention) influencing health-promoting behaviours among Malaysian university students.

**Methods:**

In a cross-sectional survey, the undergraduate students in Universiti Sains Malaysia were invited to complete the self-administered questionnaires. Participants were selected using a purposive sampling method. The proposed hypothesised model was analysed using a structural equation modelling with Mplus 7.3 program. A total of 788 (70.7% female) undergraduate students with a mean age of 20.2 (SD = 1.02) participated in the study. The primary outcome of knowledge, health beliefs, and health-promoting behaviours related to CVD were measured by questionnaires namely: Knowledge of Heart Disease, Health Beliefs Related to CVD, and Health Promoting Lifestyle Profiles-II.

**Results:**

The final hypothetical structural model showed a good fit to the data based on several fit indices: with comparative fit index (CFI) at .921, standardised root mean square residual (SRMR) at .037, and root mean square error of approximation (RMSEA) at .044 (90% CI: .032, .054). The final structural model supported 13 significant path estimates. These variables explained 12% of the total variance in health-promoting behaviours. Through perceived benefits, total knowledge had an indirect effect on health-promoting behaviours.

**Conclusion:**

The results suggest that perceived barriers, perceived benefits, family history of CVD, and screening intention enable young adults to engage in health-promoting behaviours.

## Introduction

Cardiovascular diseases (CVDs) such as coronary heart disease, hypertension, and stroke are the biggest threats to public health globally. It was estimated that 17.9 million people died from CVDs every year, representing 31% of all global deaths [1]. In Malaysia, the prevalence of lifestyles related diseases including diabetes, hypertension, and obesity are increasing at an alarming rate [2–6]. This increase will cause a major burden to the nation’s economic, society, and health care system.

Furthermore, this health issue is not only affecting older adults but also among young adults at an early stage which are asymptomatic [7]. Previous studies found that among university students, 7.5% were hypertensive, 29.8% were overweight, 26.3% had elevated blood lipid profiles, and 5.1% had elevated fasting glucose levels [8, 9]. More than half of young adults exhibit at least one CVD risk factors, and this greatly increases the lifetime heart disease risk [10]. It is imperative to focus on young adults as the onset of CVDs is a long-term exposure due to various risk factors [11]. University students are likely to engage in risky health behaviours during this period of life [12].

Most of these CVD risks are preventable by simple and affordable ways (i.e., lifestyle changes), especially at young age. However, many studies have shown that young adults are physically inactive [13–16], having unhealthy dietary habits [16–18], and smoking cigarettes [19, 20]. Unhealthy diet, physical inactivity, tobacco use, and harmful use of alcohol are the behavioural risk factors that lead to CVDs [21]. Therefore, early detection of one of these unhealthy behaviours from schooling years until early adulthood is still possible for behaviour change [22, 23].

Many existing studies address physical activity, health responsibility, and nutrition behaviour separately. However, we believe that the mentioned activities are all elements encompassed in health-promoting behaviours for CVD prevention. Therefore, based on the structure of latent variables [24, 25], all above-mentioned elements are combined in one latent variable hereby called health-promoting behaviours. Health-promoting behaviours play a major role in many lifestyle related diseases (i.e., diabetes, obesity, and hypertension) prevention. Therefore, understanding young adults’ health-promoting behaviours is crucial for developing tailored health promotion interventions and thereby improve their health.

There are relatively few applications of psychological theory to explain cardiovascular health-promoting behaviours, especially among young adults. Theory-based research is important as it provides a hypothesis testing framework for understanding the pathway leading to healthy behaviours using the theoretical constructs [26]. Gautam [27] and Tovar and Clark [28] found that knowledge significantly correlates with perceived benefit and severity related to CVD. Previous studies also showed that knowledge or awareness of CVD risk factors and demographic factors had significant relationships with health behaviour and health beliefs [29–34]. Therefore, we included the knowledge and demographic characteristics in this study to investigate its influence in the hypothesised model for adopting health-promoting behaviours related to CVD.

A review of specific behaviours for reducing CVD risks (i.e., exercise, nutrition, and smoking) had demonstrated significant relationships of perceptions of benefits, barriers, susceptibility, severity, and self-efficacy with health behaviour [31, 32, 35–37]. Other studies also identified the association between health beliefs and behavioural intention [38, 39], and between cues to action and health behaviour [33, 40]. Behavioural intention (i.e., screening intention) is included in the behaviour implementation process to understand the tendency of young adults to get health check-up toward adoption of healthy behaviour.

To date, how these factors are related to the health-promoting behaviours (i.e., nutrition, physical activity, and health responsibility) for young adults has not been fully explored. Furthermore, there is little information available regarding how strong the influence of these factors is on health-promoting behaviours in adjusted models. This study attempts to link with other important determinants of health-promoting behaviours (i.e., demographic variables, knowledge, screening intention, and self-efficacy) to understand the inter-relationships between the constructs.

**Fig 1:**
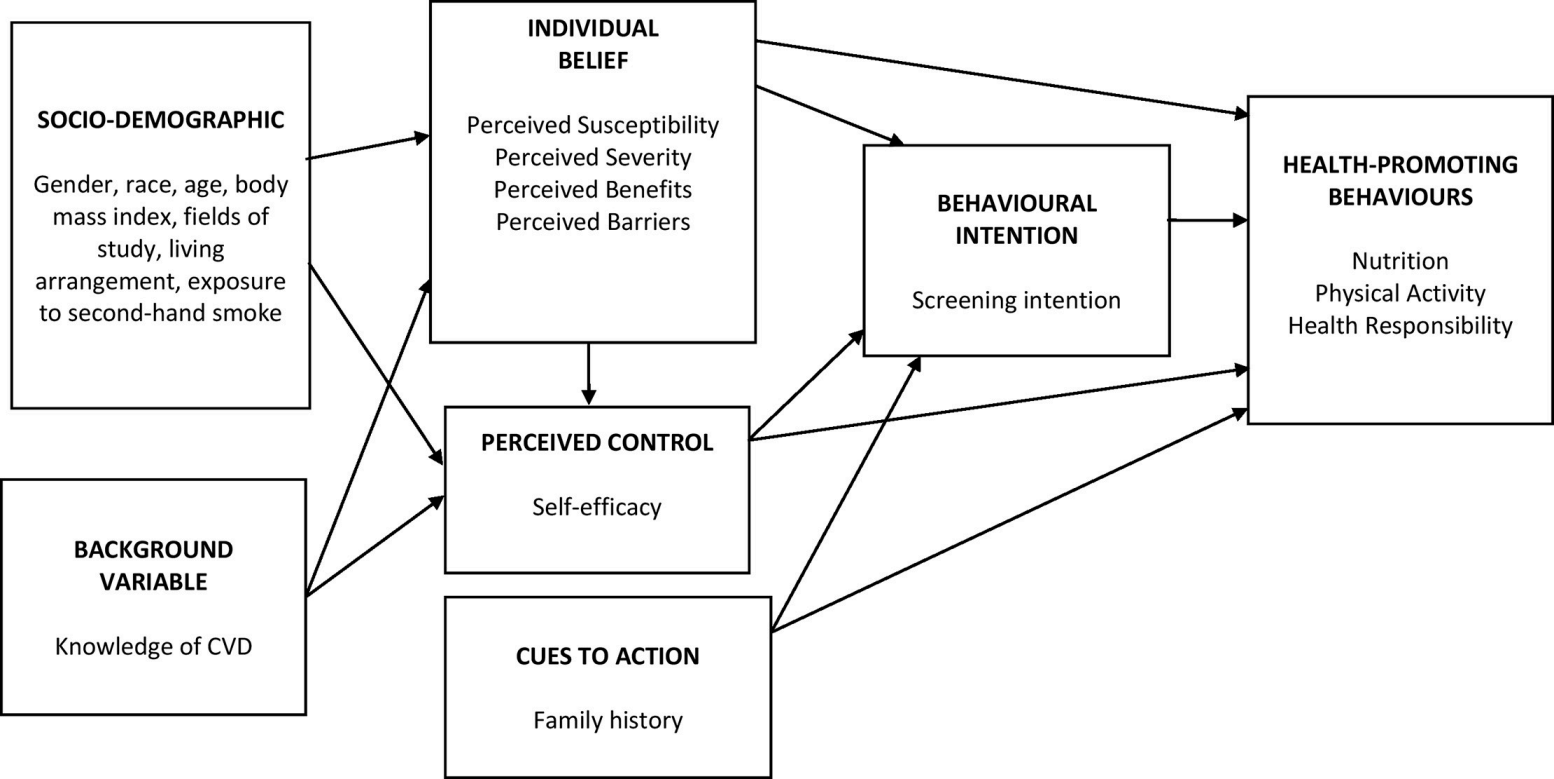
Proposed hypothesised structural model: Initial model/model 1.

We proposed a series of hypotheses that reflect the research framework, based upon demographic variables, knowledge of CVD, the health beliefs constructs, self-efficacy, and additional of screening intention concerning CVDs preventive behaviour (Fig 1). This study aimed to present a model that describes the direct and indirect relationships between the variables and the observed findings suggest an influence on the variables.

## Methods

### Study design

A cross-sectional design was adopted to explore the relationships among variables (i.e., knowledge, selected socio-demographic factors, health beliefs, self-efficacy, cues to action, and screening intention towards health-promoting behaviours) for cardiovascular disease prevention among Malaysian undergraduate university students.

### Participants and setting

This study involved 788 young adults studying in the three campuses (i.e., Main, Health, and Engineering) of Universiti Sains Malaysia (USM) from November 2014 to January 2015. Purposive sampling was adopted for the selection of eligible students in the three campuses of USM. Several faculties were selected by using a purposive sampling method to provide sufficient students. The survey questionnaire was distributed to the undergraduate students before their class started based on the programme offered by their faculties. All eligible Malaysian undergraduate students enrolled in USM. Only students who were available during the time of data collection and volunteer to participate, and capable of completing the questionnaire were recruited as participants.

### Ethical considerations

This study was approved by the Human Research Ethics Committee, USM [USM/JEPeM/14070253] on 29 October 2014 and the permission was obtained from the relevant authorities to access to the schools. Participation in the survey was voluntary and based on implied consent. Implied consent refers to consent, which is not expressly granted by a person, but rather, is implicitly granted by a person’s actions. The participants were briefed on the study purpose and the procedures before answering the questionnaire. Implied consent was obtained by the act of participants agreeing to answer the distributed questionnaire with honest responses and returned the completed questionnaire to the researcher.

### Measures

The questionnaire included a demographic form and five measurement instruments were used as data collection procedure (i.e., heart disease knowledge, health beliefs, self-efficacy, screening intention, and health-promoting behaviours).

#### Demographics and health-related information

Self-reported measures that assessed the respondent’s gender, age, weight, height, race, current living arrangement, programs of study, and current year of study. Besides, we also collected self-reported measure on family health-related information of CVD and exposure to second-hand smoke.

#### Knowledge of heart disease

The knowledge of heart disease was adopted from the 30 (true/false/don’t know) items scale [41]. We assess the validity of the scale using confirmatory factor analysis (CFA) in previous study and demonstrated a 23-item, one-factor model: weighted least squares mean and variance adjusted scaled chi-square difference = 1.22, degrees of freedom (df) = 2, *p* value *=* .544, the root mean square error of approximation (RMSEA) = .030 (90% CI: .030, .040; *p* > .950). Participant’s item answers were coded either “0” for an incorrect answer or “don’t know” and “1” for the correct answer. A higher score reflects a higher level of knowledge about heart disease. The total scores are calculated and converted to percentages [42].

#### Health beliefs related to CVD

Health belief was adopted using the Health Beliefs related to CVD (HBCVD) 25-item test [43]. The construct validity of the scale was examined using CFA in the previous study and has shown good psychometric values. The measurement model showed a 19-item and four-factor model with acceptable model fit indices: RMSEA = .040 (90% CI: .034, .046; *p* = .998); standardised root mean square residual (SRMR) = .053; Tucker-Lewis index (TLI) = .941; comparative fit index (CFI) = .950 [44]. HBCVD consists of four subscales: Each subscale has four response options from 1 = “strongly disagree” to 4 = “strongly agree”. A total subscale score is calculated for each perceived subscale. The composite reliability and the average variance extracted values for the final measurement model ranged from .611 to .882 and .254 to .592, respectively.

#### Health Promoting Lifestyle Profiles (HPLP)-II

The HPLP-II is a 52-item scale focuses on self-initiated actions and perceptions that serve to maintain or enhance the level of wellness, self-actualisation and fulfilment of the individual [45]. In the previous study, the researchers decided to stream it down to three subscales consisting of 26 items. The chosen subscales are elements encompassed in health-promoting behaviours and have a direct effect on CVD prevention: nutrition (9 items), physical activity (8 items), and health responsibility (9 items).

To ascertain these elements encompassed the health-promoting behaviours and the items are culturally appropriate, we conducted CFA in this study. The scale produces a 21-item, three-factor model, and yielded adequate goodness-of-fit values: CFI = .921; TLI = .904; SRMR = .055; RMSEA = .045 (90% CI: .040, .050; *p* = .935). The HPLP-II measurement model also showed good convergent and discriminant validity [24]. Total scores were calculated for each subscale and converted to percentages for comparison across subscales. Higher mean total scores indicate the higher tendencies to engage in health-promoting behaviours for healthier lifestyles.

#### Self-efficacy and screening intention

The researcher adopted both measures from previous studies, consisting of one-item each. Self-efficacy was evaluated with the statement “There’s nothing I can do to help prevent myself from getting heart disease” which is a 4-point Likert scale response with possible answers from 1 = “strongly disagree” to 4 = “strongly agree” [23]. Screening intention was measure using the statement “If given the opportunity free of cost, how likely would you be to make an appointment sometime soon to get screened for coronary heart disease risk-factors?”. A 9-point Likert scale response was used with possible answers from 1 = “extremely unlikely” to 9 = “extremely likely” [46].

### Data analyses

Descriptive statistics and Pearson correlation coefficients were computed for all demographic characteristics and relationship between study variables using IBM SPSS Statistics version 22.0. Any missing information on the item of interests, < 5% on a single variable was treated as non-valid case and deleted through Listwise deletion. The proposed structural model outlined in Fig 1 was evaluated with Mplus version 7.3 [47] using Structural Equation Modelling (SEM) analysis.

Item parcelling was used to produce a more parsimonious model along with a stable parameter estimate [48, 49]. All of the items in the three scales (knowledge, health beliefs, and health-promoting behaviours) were parcelled into one parcel for knowledge, four parcels for health beliefs, and three parcels for health-promoting behaviours. If the items parcelled were unidimensional, then the parcelled items were more likely to conform to the multivariate normality assumptions than the original individual items [50].

Multivariate normality assessment was performed for the initial path modelling. The normality test was based on the two-sided skewness test of fit and two-sided kurtosis test of fit with a *p* ≥ .05, which is multivariate normal [49]. In this study, the assumption of multivariate normality was not met, thus Robust Maximum Likelihood (MLR) estimator was used for SEM analysis. To evaluate model fitness for SEM, several fit indices were taken into comprehensive consideration. The fit indices used in this study included the CFI and TLI values, of which a value of .90 or greater indicate a good fit. The SRMR value less than or equal to .08 and the RMSEA value of .08 or less indicate a reasonable fit [50–53].

Subsequent path models were re-examined and modified to obtain a better fitting model if modification indices (MI) were larger than 10 [48, 49]. Significant standardised path coefficients (β) with 95% CI, standard error (SE), and statistical significance value were reported for the finalised structural model. An α of .05 was used to determine statistical significance.

### Sample size

SEM, in general, requires a large sample size but it depends on the complexity of the model tested in the analysis. A sample size of 200 or more is necessary for a complicated SEM model [52], and it also provides a sound basis for estimation [50]. Therefore, the sample for the present study of 788 participants is considered adequate.

## Results

### Preliminary analysis of data

Of the 845 questionnaires distributed, 837 were returned (99.1% response rate). Preliminary analysis of the missing information in the survey was conducted and resulting in a sample of 788 complete and usable surveys (94.1% complete and usable response rate). Missing data on demographic section was considered as unusable survey thus the cases were deleted from the initial surveys received. A total of 15 participants (out of 837) had missing data on demographic variables, while 34 participants had either one or two unanswered items which were deleted via Listwise as an incomplete survey.

### Participants characteristics

Demographic of the participants are presented in Table 1. Of the 788 participants, 557 were female (70.7%). The participants were between 19–27 years of age. Most of the participants were Malay and lived in university accommodation. About half (50.9%) of the participants were from the field of science.

**Table 1:** Socio-demographic characteristics of the sample and descriptive statistics of measured variables, (n = 788).

Variable	Frequency (n)	Percentage (%)	Mean (SD)
Gender			
Female	557	70.7	
Male	231	29.3	
Ethnicity			
Malay	375	47.6	
Chinese	338	42.9	
Indian	48	6.1	
Others	27	3.4	
Living arrangement			
On campus	707	89.7	
Off campus	81	10.3	
Fields of study			
Arts	165	20.9	
Sciences	401	50.9	
Technical	222	28.2	
Year of study			
First	421	53.4	
Second	234	29.7	
Third	133	16.9	
Age (years)			20.2 (1.02)
BMI (kg/m^2^)			21.2 (4.03)
Total knowledge			15.0 (3.79)
Dietary^a^		61.8	
Epidemiology^a^		47.7	
Risk factor^a^		52.9	
Medical^a^		41.0	
Symptom^a^		43.8	
Perceived susceptibility			10.5 (2.71)
Perceived severity			12.7 (2.61)
Perceived benefits			20.2 (3.59)
Perceived barriers			20.9 (3.49)
Nutrition			21.8 (3.98)
Physical activity			17.6 (3.87)
Health responsibility			17.7 (4.67)

^a^Percentage of the correct score, SD = standard deviation.

Overall, the participants scored just above the average for total knowledge of heart disease score (mean = 15.0, SD = 3.79) with the highest percentage of correct score noted in dietary knowledge. Participants experience the barriers to engage in diet and exercise for cardiovascular disease prevention; however, they scored higher in terms of nutrition behaviour compared to exercise and health responsibility (Table 1).

### Structural model

The hypothesised model was tested based on the empirical findings from previous research and theoretical framework (Fig 1). Model 1 was tested and revealed that some path relationships were not significant. Fit indices result for model 1 were not within the acceptable range of acceptable fitness except for SRMR. Non-significant paths between variables (standardised path coefficient) that did not explain much of the model were removed iteratively. The model was then re-tested and evaluated for fitness repeatedly.

The non-significant paths, which were removed, were pathways linking gender, exposure, and race to individual beliefs (susceptibility, severity, benefits, and barriers); gender, exposure, and race to self-efficacy. Further, the pathway that links self-efficacy and intention; individual beliefs (susceptibility and severity) to health-promoting behaviours) were also excluded. This resulted in the revised model (i.e., model 2). The path relationships were reduced to 13 significant paths and the model was examined for the goodness of fit test.

**Fig 2:**
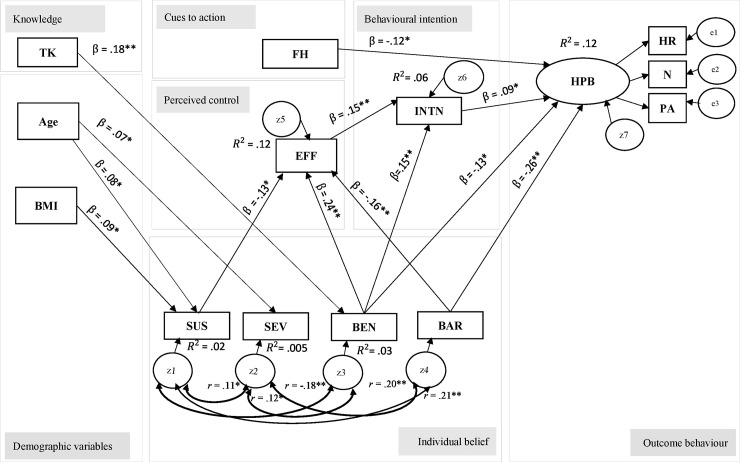
Hypothesised structural model: Final model/model 3. *P ≤ .05, **P ≤ .001, z1-z7 and e1-e3 = disturbances (error in measurement), *r* = correlation coefficient, TK = total knowledge, BMI = body mass index, SUS = Susceptibility, SEV = Severity, BEN = Benefits, BAR = Barriers, EFF Self-efficacy, INTN = screening intention, HPB = health-promoting behaviours, FH = family history of CVD, R^2^ = final coefficient of determination, H = hypothesis.

As reported in Table 2, the fitness for model 2 was still not within the acceptable range of the recommended threshold values. Further re-specification of the model was required through the suggestion from MI and also based on adequate theoretical support. Additional paths or correlations were added in model 3 or final structural model iteratively (Fig 2).

**Table 2:** Summary of goodness-of-fit indices: Hypothesised structural model.

Model	CFI	TLI	SRMR	RMSEA (90% CI)	*P* *
**Initial/Model 1**	.773	.551	.047	.079 (.071, .088)	< .001
**Model 2**	.820	.760	.055	.066 (.570, .075)	.001
**Final/Model 3**	.921	.892	.037	.044 (.032, .054)	.829

CFI = comparative fit index, TLI = Tucker-Lewis fit index, SRMR = standardisation of root mean square residual, RMSEA = root mean square error of approximation

*RMSEA Probability, CI = confidence interval.

Modification index suggested that additional path relationships should be added to improve the model fitness. Adequate theoretical support was carried out to investigate the path relationships suggested through MI. The first modification was the correlation between individual belief variables (susceptibility, severity, benefits, and barriers). The paths were added into the model one at the time and the model was evaluated each time new paths were added in Fig 2.

The final structural model was obtained after the addition of five correlations (double head arrow) between individual belief variables and significant variables in the path relationships. No further additional path was suggested from MI after addition of the correlation between individual belief variables. The parsimonious model achieved with additional of the correlations between the individual beliefs variables. From the hypothesised model, only 13 hypotheses were supported.

Fig 2 and Table 3 depict the final structural model and the standardised path estimation with its 95% CI for the significant path relationships. The model fit indicators demonstrated a good fit of data: CFI = .921, SRMR = .037, and RMSEA = .044 (90% CI: .032, .054; *P* = .829), except for TLI = .892 (Table 2). Furthermore, the final model also showed that the hypothesised model explained a statistically significant amount of variance for each latent variable except for perceived severity, R^2^ = .005, *P* = .227. Overall, the final model explained only 12% of the variance in health-promoting behaviours and explained only 2% of the variance in perceived susceptible, 3% of the variance in perceived benefits, 12% of the variance in self-efficacy, and 6% in screening intention (Fig 2).

**Table 3:** Path relationships of the final model.

Relationships			β (95%CI)	*P*
SUS	←	Age	.08 (.02, .15)	.014
SEV	←	Age	.07 (.01, .13)	.015
SUS	←	BMI	.09 (.02, .16)	.009
BEN	←	TK	.17 (.11, .24)	< .001
EFF	←	SUS	- .13 (- .20,—.05)	.001
EFF	←	BEN	.24 (.16, .32)	< .001
EFF	←	BAR	- .16 (- .23,—.08)	< .001
INTN	←	BEN	.15 (.07, .23)	< .001
HPB	←	BEN	-.13 (- .21,—.04)	.005
HPB	←	BAR	-.27 (- .36,—.18)	< .001
INTN	←	EFF	.15 (.07, .23)	< .001
HPB	←	FH	-.12 (- .20,—.05)	.003
HPB	←	INTN	.09 (.01, .17)	.026

β = standardized regression weights of pathways, SE = standard errors, CI = confidence interval, SUS = perceived susceptibility, SEV = perceived severity, BEN = perceived benefits, BAR = perceived barriers, TK = total knowledge, INTN = screening intention, EFF = self-efficacy, BMI = body mass index, HPB = health-promoting behaviours, FH = family history of CVD.

### Structural model testing for indirect pathways

In this study, we found that total knowledge had a significant indirect relationship on health-promoting behaviours through perceived benefits (*P* = .012) as reported in Table 4.

**Table 4:** Standardised direct, total indirect, and total effect.

Predictor variables	Through	Causal effect		
		Direct	Indirect	Total
**Self-efficacy → HPB**				
Self-efficacy	INTN	.016 (*P* = .754)	.013 (*P* = .056)	.029 (*P* = .565)
**Total knowledge → HPB**				
Total knowledge	BEN	.022 (*P* = .602)	- .023 (*P* = .012)*	.002 (*P* = .964)
	INTN, BEN		.002 (*P* = .074)	
	INTN, BEN, Self-efficacy		.001 (*P* = .086)	

* significant results at *P* < .05 highlighted in bold.

HPB = health-promoting behaviours, INTN = screening intention, BEN = perceived benefits

## Discussion

This study aimed to test a hypothesised model of associations between selected demographic variables, knowledge of heart disease, health belief related to CVD, self-efficacy, cues to action, and screening intention towards health-promoting behaviours among Malaysian university students. Following some modifications, the final hypothesised model fit the data well (Fig 2). Besides, the results support the previous studies and enhance our understanding of how selected demographic variables, knowledge of heart disease, health belief related to CVD, self-efficacy, cues to action, and screening intention influence health-promoting behaviours based on Health Behaviour Model (HBM). In this study, we identified 13 significant direct structural paths coefficients and able to explain the determinants of health-promoting behaviours.

The present study found that demographic variables (i.e., age) exerted significant effects on health belief (i.e., perceived susceptibility and severity) which is consistent with the results of some previous studies [27, 34]. Gautam [27] found that age was correlated and had a significant direct effect with perceived severity among university students. Ammouri and Neuberger [54] found a significant relationship between age and perceived risk towards heart disease among adults. There is some dissimilarity among the current research and study by Ammouri et al. [34], where adults’ sample was used and the scale for risk perception is measuring an individual’s perception of his/her risk of getting heart disease, which could be a scale that covers both susceptibility and severity constructs in HBM. The results indicated that the greater the age, the higher the perceived susceptibility and severity towards CVD among young adults.

The significant path from BMI to perceive susceptibility indicates that those with greater BMI had a higher level of perceived susceptibility on CVD. The relationship was not consistent with past studies [27, 36, 38]. Saghafi-Asl, Aliasgharzadeh [38] were comparing the reverse relationship between HBM constructs and BMI. Further research is, therefore, necessary to investigate on how to target those with high BMI group for disseminating the risk of CVD and how to reduce the risk through the engagement of healthy behaviours. The magnitude of the regression weight for the pathways (i.e., age → perceived susceptibility and age → perceived severity) in Fig 2 were less than 10%. It may be due to the lack of awareness among young adults about their susceptibility and severity towards CVD in this study.

Our findings are consistent with previous studies [27, 35], who found that there were no significant relationships between race and individual beliefs constructs. Similarly, we also found that there was no significant relationship between other background variables (i.e., gender, living arrangement, the field of study, and exposure to second-hand smoke) to HBM constructs. However, these findings should not be interpreted as a lack of support for the importance of understanding health beliefs of CVD. For example, there could be a possibility that the perception between the ethnics group might be different, as shown in the Malaysian National Cardiovascular Disease database from 2006–2010 on ethnic differences concerning heart disease [55]. These paths need to be further examined to understand how difference socio-demographic variables among young adults perceived the CVD risk and engagement in health-promoting behaviours.

Moreover, we also found that total knowledge had a significant relationship with perceived benefits. Previous studies did not determine the relationships between knowledge to individual beliefs and treated the knowledge as endogenous variables [27, 36, 56, 57]. Knowledge of CVD was compared between the selected demographic factors in past studies. In this study, it was noted that those who are knowledgeable would perceive higher the benefits to reduce CVD risks. The significant result was supported by the Health Belief Model (HBM) theoretical framework [58, 59]. The present study showed that health educators in university should continue to disseminate information on CVD. By increasing their awareness of CVD, the students may learn more and thus, change their attitude or beliefs.

The direct path from individual beliefs constructs to self-efficacy and screening intention can be interpreted in light of HBM. Individuals who perceived the benefits of diet and exercise for reducing the risk of heart disease would be expected to have more confidence to engage in a particular activity to prevent themselves of getting heart disease (i.e., self-efficacy). In this study, we found that all except perceived severity influenced self-efficacy. The level of barrier to perform the exercise and eat healthily could lead to low self-efficacy among young adults’ ability to prevent themselves from CVD. Winham and Jones [23] compared the reverse path, from self-efficacy to perceived susceptibility to heart disease and found not significant. The present results from perceived susceptibility and perceived barriers to self-efficacy yielded an interesting finding that the higher the student’s perceived the level of susceptibility of contracting CVD and perceived barriers of engaging in healthy diet and exercise, the lower the student’s self-efficacy. There was a tendency that when the participants had been contracting with CVD, they felt hopeless and would not do anything to reduce the risk because they did not have prior knowledge on CVD.

The path from perceived benefits to screening intention was found to be significant (β = .15, *p* < .001). It was consistent with Kim, Ahn [31], who also found a significant path from perceived benefits and behavioural intention for eating healthily and performing the exercise. This path indicates that those who perceived a higher level of benefits would be more likely to participate in coronary heart disease (CHD) risk screening. The majority of the students in the current study had a positive attitude on screening for CHD risk with a mean percentage of 80%. Perceived susceptibility and severity are related to self-efficacy. However, there were lack of studies that examined the respective relationships. According to Champion and Skinner [60], the relationships between the HBM constructs are not defined. Further study is, therefore, necessary to ascertain the relationships between individual beliefs and self-efficacy among young adults.

Furthermore, screening of CHD risk was important among young adults as it helped to detect risk factors at an early age. The earliest age recommended by American Heart Association for cardiovascular risk screening (i.e., blood pressure, cholesterol, weight, waist circumference, dietary habit, smoking habit, and physical activity habit) starting at age 20 [61]. Malaysian clinical guideline did not specifically mention the earliest age for CHD risk screening. Malcom [46] did propose the inclusion of screening intention for their regression model because there was a lack of participation on actual screening intention.

Of the individual beliefs constructs, perceived barriers was found to be the most significant contributing factor of health-promoting behaviours, β = .26, but a negative direction followed by perceived benefits. Previous studies found that perceived barriers and benefits were associated with health behaviours [32, 33]. The concept of perceived barriers is an important predictor of health-promoting behaviour [59]. Findings of this study also suggest health educators should expend energy and resources toward early participation in health-promoting behaviours and reduction of barriers among young adults.

Young adults’ belief that exercise and a good diet can help them to prevent CVD but there may have other psychological factors such as emotional, or time factor to engage in healthy behaviours and they may have concerned more on their study rather than exercise or getting healthy food. There are inconsistencies between our study and the study by Patel [36]. The possibility could be that the population in Patel [36]’s study was working women which may have other expectation such as carrier, family or childcare to be taken care of compared to undergraduate students which only concern on their studies.

The final model also found that there was a significant relationship between self-efficacy and screening intention. The reason could be the sample in this study who have a high level self-efficacy also had a higher intention in getting screened for CHD risk. The direct path from self-efficacy to health-promoting behaviours was found to be insignificant which was inconsistent with the previous study [35, 38]. This may be due to the HPLP-II with six dimensions and health self-efficacy scales used in Jackson, Tucker [35] study was different from the present study. Saghafi-Asl, Aliasgharzadeh [38] found that self-efficacy in exercise and self-efficacy in dieting scales was significantly related to the greater behavioural intention of weight management.

The current study raises the interesting possibility that family history has a negative effect on health-promoting behaviours (β = −.12, *P* = .003). Those with a family history of CVD will be less likely to engage in health-promoting behaviours compared to those without a family history of CVD. The negative relationship could be due to fear of the outcome or and other factors such as time factor and they would not want to participate in any health-protective behaviour. These factors were uncovered in the present study but needed to be further explored. Little research has been focused on the impact of the cue to action on health-promoting behaviour. However, Andersson, Sjöberg [62] reported that there is no effect of family history of CVD on BMI level, smoking, and exercise habits conducted among the adult population. Rahmati-Najarkolaei, Tavafian [32] reported that there is no significant relationship between cues to action on physical activity behaviour and nutrition behaviour among university students.

Finally, the indirect pathway from knowledge to health-promoting behaviours was tested and it raises interesting finding that total knowledge had a significant indirect relationship with health-promoting behaviours through perceived benefits. It may be small mediation effects of β = —.023, *P* < .012 but still significant. Those who are knowledgeable does not necessary to engage in healthy behaviours alone but through perceived benefits of doing exercise or having good dietary habits can influence the health-promoting behaviours. However, no previous research has explored the indirect path from knowledge toward health-promoting behaviour. Previous studies demonstrated the significant direct path from knowledge to health behaviour [30, 56, 57]. The role of health knowledge plays in health behaviour change needs to be further examined [56].

### Strengths, limitations, and future directions

The present research provides useful information on a theory-based investigation of how the relationships from starting background variables (total knowledge and selected demographic variables) might affect the health-promoting behaviours (i.e., physical activity, nutrition, and health responsibility) among young adults in a single model. Important influences (direct and indirect effect) of health-promoting behaviours also obtained using the SEM approach. The current study makes an essential contribution to understanding university students’ psychological behaviours to prevent CVD. First, it identifies some of the important determinants of health promoting behaviours. The findings demonstrated that health promoting behaviours was determined in order of importance by perceived benefits, family history of CVD, screening intention, and perceived barriers. It is suggested that understanding of the health belief and health promoting behaviours among young adults helps to eliminate health disparities related to CVD.

Secondly, another contribution of this research was the identification of meaningful indirect predictors of health promoting behaviours from total knowledge. SEM analysis allowed us to test multiple relationships simultaneously within a conceptual model. We believe that this is the first study to examine perceived benefits as potential mediators between total knowledge and health promoting behaviours. A better understanding of the complex relationship between sociodemographic factors, total knowledge, health beliefs, and health promoting behaviours is critical as we continue to reduce the burden of CVD, especially among young adults.

Finally, as the population were young adults, the health care providers could not directly screen them for CHD risk factors. Meanwhile, we presented novel results regarding the university student’s screening intention of CHD risk factors, aiming to build an insight into future public health programs that target individuals with a high risk of getting CHD. This could support bringing positive health promotion behaviours among them.

There are several limitations to this study. First, the use of self-report BMI, smoking status, and exposure to second-hand smoke measures might result in the inaccuracy of interpreting the data. Secondly, the participants are also subject to socially desirable response (i.e., the desire to answer the question in a socially acceptable manner) [63]. To overcome these, the researchers put in efforts to emphasise more on the study procedure during the briefing session for completing the survey. Thirdly, the response of the study consisted of mainly female, about 70.7% of the sample. This disparity reflects the proportion of students from the public universities in Malaysia over the last two decades, which female comprises about two-thirds of the student population [64]. Previous research at Malaysian public universities also found a high proportion of female respondents, varying from 62 to 80% [65–67]. Future research should include a stratified sampling to obtain the representation of university student. Finally, due to the cross-sectional design employed, the causality of the observed relationships was not demonstrated.

The model was specified in accord with the research framework. Still, future work using longitudinal (i.e., cross-sequential design) or experimental designs would provide greater insight into the causal relationship of these hypothesised associations. Future research is warranted to investigate whether the present findings can be usefully translated into practice. As a first step, it would be informative to test whether it is more productive in practice to focus on inclusion of other variables (i.e., health motivation, health value, stress management, and actual behavioural patterns) in the model. Additional research should also include a multi-group comparison of the structural model based on the field of study (e.g. science/ arts/ technical) and gender (e.g. male/female).

## Conclusion

The theoretical model was verified in the study. Our findings are consistent with both the theoretical tenets of the health behaviour model and other empirical research on young adult’s physical activity, weight management, and nutrition behaviour [31, 32, 38, 39]. The findings suggested that health-promoting behaviours can be explained by perceived barriers, perceived benefits, cues to action, and screening intention. Moreover, this study could be beneficial for improving health responsibility, nutrition, and exercise behaviour among young adults.

## Supporting information

S1 Data (PDF)
