# Supplementary material for: Modelling knowledge, health beliefs, and health-promoting behaviours related to cardiovascular disease prevention among Malaysian university students
Source: PLoS One. 2021 Apr 28;16(4):e0250627. doi: 10.1371/journal.pone.0250627 (PMC8081192; doi:10.1371/journal.pone.0250627)
Supplement: S1 Data — (PDF) [file pone.0250627.s001.pdf]

newID	gender	religion	age	race	recrace	ht	wt	BMI	living
1	1	3	19	3	2	1.62	50.7	19.3187	1
2	1	1	19	1	1	1.58	56	22.4323	1
3	2	1	19	1	1	1.69	47	16.45601	1
4	1	1	20	1	1	1.63	52	19.57168	1
5	1	4	20	4	2	1.53	60	25.63117	1
6	2	1	20	1	1	1.7	55	19.03114	1
7	2	1	20	1	1	1.73	68	22.72044	1
8	2	1	20	1	1	1.55	65	27.05515	1
9	2	1	20	1	1	1.64	54	20.07733	1
10	2	1	20	1	1	1.63	80	30.11028	1
11	1	1	19	1	1	1.6	61	23.82813	1
12	1	1	19	1	1	1.5	46	20.44444	1
13	1	1	19	1	1	1.62	50	19.05197	1
14	1	1	19	1	1	1.64	43	15.98751	1
15	1	1	19	1	1	1.58	45	18.02596	1
16	1	1	19	1	1	1.56	46	18.90204	1
17	1	1	19	1	1	1.52	87	37.65582	1
18	1	1	19	1	1	1.53	53	22.64086	1
19	1	1	19	1	1	1.73	72	24.05693	1
20	1	1	19	1	1	1.6	47	18.35938	1
21	1	5	21	2	2	1.53	46	19.65056	1
22	1	1	19	1	1	1.52	42	18.17867	1
23	1	1	19	1	1	1.56	47	19.31295	1
24	1	1	19	1	1	1.54	50	21.08281	1
25	1	1	19	1	1	1.6	50	19.53125	1
26	1	2	20	2	2	1.58	51	20.42942	1
27	1	1	19	4	2	1.53	65	27.7671	1
28	1	1	20	1	1	1.53	50	21.35931	1
29	1	2	20	2	2	1.66	52	18.87066	1
30	1	1	19	1	1	1.52	48	20.77562	1
31	1	4	19	4	2	1.65	59	21.67126	1
32	1	1	19	1	1	1.44	40	19.29012	1
33	1	1	21	1	1	1.59	44	17.40437	1
34	1	1	20	1	1	1.58	62	24.83576	1
35	1	1	19	1	1	1.52	53	22.93975	1
36	1	1	19	1	1	1.56	56	23.01118	1
37	1	1	19	1	1	1.59	50	19.7777	1
38	1	1	19	1	1	1.56	58	23.833	1
39	1	2	20	2	2	1.55	50	20.81165	1
40	1	2	20	2	2	1.66	53	19.23356	1
41	2	2	19	2	2	1.75	60	19.59184	1
42	2	2	20	2	2	1.72	55	18.59113	1
43	2	2	20	2	2	1.74	65	21.46915	1
44	2	2	20	2	2	1.78	55	17.35892	1
45	1	1	20	1	1	1.5	41	18.22222	1
46	2	1	25	1	1	1.65	72	26.44628	1
47	1	1	19	1	1	1.5	72	32	1
48	1	1	19	1	1	1.63	60	22.58271	1
49	1	2	20	2	2	1.68	55	19.48696	1

50	1	4	19	4	2	1.59	47	18.59104	1
51	1	1	19	1	1	1.45	40	19.02497	1
52	1	1	19	1	1	1.59	44	17.40437	1
53	1	1	20	1	1	1.55	47	19.56296	1
54	1	2	20	2	2	1.65	51	18.73278	1
55	1	2	20	2	2	1.53	51	21.78649	1
56	1	2	19	2	2	1.67	56	20.0796	1
57	2	1	20	1	1	1.75	69	22.53061	1
58	2	1	20	1	1	1.72	70	23.66144	1
59	2	1	20	1	1	1.73	68	22.72044	1
60	2	1	20	1	1	1.82	60	18.11375	1
61	1	1	19	1	1	1.56	51	20.95661	1
62	1	1	19	1	1	1.46	34	15.95046	1
63	1	3	20	3	2	1.78	85	26.82742	1
64	1	1	19	1	1	1.7	56	19.37716	1
65	1	1	19	1	1	1.58	54	21.63115	1
66	1	1	19	1	1	1.57	47	19.06771	1
67	2	1	20	1	1	1.65	79	29.01745	1
68	1	1	20	1	1	1.49	50	22.52151	1
69	1	4	20	2	2	1.62	45	17.14678	1
70	1	1	20	1	1	1.57	55.5	22.51613	1
71	1	1	21	1	1	1.57	40	16.22784	1
72	1	1	19	1	1	1.49	49	22.07108	1
73	1	1	19	1	1	1.55	45	18.73049	1
74	1	4	19	4	2	1.51	44	19.2974	1
75	1	4	19	4	2	1.52	41	17.74584	1
76	1	1	27	1	1	1.57	52	21.09619	1
77	1	1	19	1	1	1.58	50	20.02884	1
78	1	1	19	1	1	1.51	46	20.17455	1
79	1	3	19	3	2	1.64	51	18.96193	1
80	1	3	19	3	2	1.63	45	16.93703	1
81	1	3	20	3	2	1.62	46	17.52782	1
82	2	2	21	2	2	1.73	60	20.04745	1
83	1	4	22	2	2	1.56	45	18.49112	1
84	1	1	21	1	1	1.61	52	20.06095	1
85	1	1	19	1	1	1.71	62	21.20311	1
86	1	1	19	1	1	1.57	45	18.25632	1
87	1	1	19	1	1	1.54	75	31.62422	1
88	1	1	20	1	1	1.65	45	16.52893	1
89	2	1	19	1	1	1.67	76	27.25089	1
90	1	1	20	1	1	1.58	75	30.04326	1
91	1	1	20	1	1	1.55	80	33.29865	1
92	1	1	20	1	1	1.59	47	18.59104	1
93	1	1	20	1	1	1.56	55	22.60026	1
94	1	1	21	1	1	1.53	50	21.35931	1
95	1	1	21	1	1	1.4	40	20.40816	1
96	1	1	20	1	1	1.52	52	22.50693	1
97	1	1	20	1	1	1.59	47	18.59104	1
98	1	1	20	1	1	1.62	57	21.71925	1
99	1	1	19	1	1	1.54	47.7	20.113	1

100	1	1	19	1	1	1.56	68	27.94214	1
101	1	1	19	1	1	1.54	55	23.19109	1
102	1	1	19	1	1	1.59	50	19.7777	1
103	1	1	19	1	1	1.56	70	28.76397	1
104	1	1	19	1	1	1.55	46	19.14672	1
105	1	1	19	1	1	1.61	52	20.06095	1
106	1	1	19	1	1	1.54	50	21.08281	1
107	1	1	19	1	1	1.55	44	18.31426	1
108	1	2	20	2	2	1.53	43	18.369	1
109	1	1	20	1	1	1.57	47	19.06771	1
110	1	1	20	4	2	1.56	45	18.49112	1
111	1	1	20	1	1	1.57	58	23.53037	1
112	1	1	19	1	1	1.6	53	20.70313	1
113	1	1	19	1	1	1.52	52	22.50693	1
114	1	1	19	1	1	1.5	59	26.22222	1
115	1	1	19	1	1	1.55	43	17.89802	1
116	1	1	19	1	1	1.62	63	24.00549	1
117	1	1	19	1	1	1.48	39	17.80497	1
118	1	1	19	1	1	1.52	56	24.23823	1
119	2	1	20	1	1	1.69	53	18.55677	1
120	1	1	19	1	1	1.56	43	17.6693	1
121	2	1	19	1	1	1.7	55	19.03114	1
122	1	1	21	1	1	1.62	68	25.91068	1
123	1	1	22	1	1	1.62	73	27.81588	1
124	1	1	19	1	1	1.54	68	28.67263	1
125	1	1	19	1	1	1.53	37	15.80589	1
126	1	1	21	1	1	1.59	55	21.75547	1
127	1	1	19	1	1	1.56	55	22.60026	1
128	1	2	20	2	2	1.59	45	17.79993	1
129	1	2	19	4	2	1.54	45	18.97453	1
130	1	2	19	4	2	1.65	53	19.4674	1
131	1	1	19	1	1	1.6	40	15.625	1
132	1	1	19	1	1	1.58	63	25.23634	1
133	1	1	19	1	1	1.6	65	25.39063	1
134	2	2	20	2	2	1.7	60	20.76125	1
135	2	2	20	2	2	1.76	62	20.0155	1
136	1	1	20	1	1	1.51	72	31.57756	1
137	1	1	19	1	1	1.58	50	20.02884	1
138	1	2	20	2	2	1.64	46	17.10291	1
139	2	2	21	2	2	1.79	74	23.09541	1
140	1	1	20	1	1	1.6	51	19.92188	1
141	1	1	20	1	1	1.63	57	21.45357	1
142	1	1	19	1	1	1.57	65	26.37024	1
143	1	1	20	1	1	1.63	57	21.45357	1
144	1	1	20	1	1	1.65	43	15.79431	1
145	1	4	20	2	2	1.68	52	18.42404	1
146	1	2	20	2	2	1.58	47	18.82711	1
147	1	3	20	3	2	1.58	45	18.02596	1
148	1	1	20	1	1	1.52	64	27.70083	1
149	1	1	19	1	1	1.55	50	20.81165	1

150	1	3	20	3	2	1.6	63	24.60938	1
151	1	2	20	2	2	1.57	39	15.82214	1
152	1	1	20	1	1	1.7	65	22.49135	1
153	1	1	19	1	1	1.58	50	20.02884	1
154	1	1	19	1	1	1.65	62	22.77319	1
155	1	1	20	1	1	1.63	60	22.58271	1
156	1	2	20	2	2	1.63	45	16.93703	1
157	1	3	20	3	2	1.6	60	23.4375	1
158	1	3	20	3	2	1.55	53	22.06035	1
159	1	1	19	1	1	1.56	47	19.31295	1
160	1	1	19	1	1	1.67	54	19.36247	1
161	1	4	20	3	2	1.48	37	16.89189	1
162	1	4	20	2	2	1.68	55	19.48696	1
163	1	4	20	2	2	1.66	60	21.77384	1
164	1	4	20	3	2	1.65	67	24.60973	1
165	1	2	19	2	2	1.64	62	23.05175	1
166	1	2	20	2	2	1.54	53	22.34778	1
167	1	4	20	4	2	1.53	56	23.92242	1
168	1	2	20	2	2	1.6	50	19.53125	1
169	1	1	20	1	1	1.51	48	21.05171	1
170	1	4	20	2	2	1.58	50	20.02884	1
171	2	2	19	2	2	1.67	82	29.40227	1
172	2	5	19	2	2	1.76	56	18.07851	1
173	1	1	19	1	1	1.58	48	19.22769	1
174	1	1	19	1	1	1.7	88	30.44983	1
175	1	1	20	1	1	1.6	45	17.57813	1
176	1	1	20	1	1	1.58	60	24.03461	1
177	1	1	20	1	1	1.54	58.2	24.54039	1
178	1	1	20	1	1	1.63	77	28.98114	1
179	1	3	21	3	2	1.57	43	17.44493	1
180	1	1	20	1	1	1.52	47	20.3428	1
181	2	3	20	3	2	1.61	45	17.36044	1
182	2	4	20	2	2	1.72	55	18.59113	1
183	1	1	20	1	1	1.53	63	26.91273	1
184	1	3	19	3	2	1.61	37	14.27414	1
185	1	3	20	3	2	1.47	46	21.28743	1
186	1	3	20	3	2	1.56	46	18.90204	1
187	2	2	20	2	2	1.69	57	19.95728	1
188	2	2	20	2	2	1.67	55	19.72104	1
189	1	2	20	2	2	1.59	56	22.15102	1
190	1	2	20	2	2	1.57	51	20.69049	1
191	1	2	20	2	2	1.67	52	18.64534	1
192	1	2	20	2	2	1.68	53	18.77834	1
193	1	1	20	1	1	1.61	55	21.21832	1
194	1	1	20	1	1	1.56	45	18.49112	1
195	1	1	20	1	1	1.61	52	20.06095	1
196	1	3	20	3	2	1.58	41	16.42365	1
197	1	3	20	3	2	1.67	55	19.72104	1
198	1	1	21	1	1	1.6	50	19.53125	1
199	1	1	21	1	1	1.58	70	28.04038	1

200	1	1	21	1	1	1.5	40	17.77778	1
201	1	2	20	2	2	1.65	63	23.1405	1
202	2	3	20	3	2	1.8	80	24.69136	1
203	1	2	20	2	2	1.53	40	17.08744	1
204	1	2	20	2	2	1.58	50	20.02884	1
205	1	1	20	1	1	1.59	50	19.7777	1
206	1	1	21	1	1	1.53	48	20.50493	1
207	1	1	19	1	1	1.62	62	23.62445	1
208	1	1	20	1	1	1.5	54	24	1
209	1	1	20	1	1	1.61	54	20.83253	1
210	1	2	20	2	2	1.58	49	19.62826	1
211	2	5	20	4	2	1.68	70	24.80159	1
212	2	4	21	2	2	1.74	76	25.10239	1
213	2	2	20	2	2	1.78	60	18.937	1
214	1	1	19	1	1	1.57	54	21.90758	1
215	1	1	19	1	1	1.51	50	21.92886	1
216	2	2	20	2	2	1.7	80	27.68166	1
217	1	4	20	2	2	1.63	48	18.06617	1
218	1	2	20	2	2	1.61	47	18.13202	1
219	1	1	19	1	1	1.57	43	17.44493	1
220	1	1	19	1	1	1.59	51	20.17325	1
221	1	2	19	2	2	1.52	45	19.47715	1
222	1	2	19	2	2	1.54	44	18.55288	1
223	1	2	20	2	2	1.64	52	19.33373	1
224	1	2	20	2	2	1.53	52	22.21368	1
225	1	2	19	2	2	1.66	50	18.14487	1
226	2	3	19	3	2	1.83	71	21.20099	1
227	2	2	19	2	2	1.7	55	19.03114	1
228	2	4	19	2	2	1.7	78	26.98962	1
229	1	1	19	1	1	1.6	53	20.70313	1
230	1	1	19	1	1	1.64	44	16.35931	1
231	1	2	19	2	2	1.59	45	17.79993	1
232	1	2	20	2	2	1.6	44	17.1875	1
233	1	2	20	2	2	1.72	51	17.23905	1
234	1	2	19	2	2	1.63	51	19.1953	1
235	1	2	19	2	2	1.53	48	20.50493	1
236	1	2	19	2	2	1.67	48	17.21109	1
237	1	4	19	2	2	1.64	51	18.96193	1
238	1	2	19	2	2	1.65	54	19.83471	1
239	1	2	19	2	2	1.7	55	19.03114	1
240	1	2	20	2	2	1.51	43	18.85882	1
241	1	2	20	2	2	1.62	40	15.24158	1
242	2	2	19	2	2	1.73	63	21.04982	1
243	2	2	20	2	2	1.67	59	21.15529	1
244	1	2	19	2	2	1.56	55	22.60026	1
245	2	2	20	2	2	1.69	65	22.75831	1
246	2	1	19	1	1	1.77	72	22.9819	1
247	2	1	19	1	1	1.6	64	25	1
248	2	1	19	1	1	1.63	61	22.95909	1
249	1	2	19	2	2	1.62	40	15.24158	1

250	1	2	19	2	2	1.53	46	19.65056	1
251	1	4	19	2	2	1.7	76	26.29758	1
252	1	2	19	2	2	1.58	50	20.02884	1
253	1	1	19	1	1	1.55	50	20.81165	1
254	1	1	19	1	1	1.51	44	19.2974	1
255	1	1	19	1	1	1.62	69	26.29172	1
256	1	1	19	1	1	1.51	50	21.92886	1
257	1	1	19	1	1	1.64	52	19.33373	1
258	1	1	19	1	1	1.54	45	18.97453	1
259	1	1	19	1	1	1.53	46	19.65056	1
260	1	1	19	1	1	1.6	49	19.14063	1
261	1	1	19	1	1	1.57	60	24.34176	1
262	2	1	20	1	1	1.72	72	24.33748	1
263	1	3	19	3	2	1.58	43	17.2248	1
264	2	4	19	4	2	1.76	86	27.76343	1
265	1	4	19	4	2	1.56	50	20.54569	1
266	1	1	19	1	1	1.53	55	23.49524	1
267	1	1	19	1	1	1.61	63	24.30462	1
268	1	2	19	2	2	1.61	44	16.97465	1
269	1	2	19	2	2	1.64	38	14.12849	1
270	1	2	19	2	2	1.55	45	18.73049	1
271	1	4	19	2	2	1.6	47	18.35938	1
272	2	1	19	1	1	1.74	63.5	20.97371	1
273	1	2	20	2	2	1.56	44	18.08021	1
274	1	4	19	2	2	1.61	63	24.30462	1
275	1	4	19	2	2	1.57	50	20.2848	1
276	1	1	19	1	1	1.55	46	19.14672	1
277	1	1	19	1	1	1.61	52	20.06095	1
278	1	3	19	3	2	1.67	47	16.85252	1
279	1	3	19	3	2	1.64	67	24.91077	1
280	1	3	19	3	2	1.67	49	17.56965	1
281	1	3	19	3	2	1.57	55	22.31328	1
282	2	5	19	2	2	1.71	50	17.09928	1
283	1	2	20	2	2	1.7	60	20.76125	1
284	2	2	19	2	2	1.68	60	21.2585	1
285	1	2	20	2	2	1.68	51	18.06973	1
286	1	2	19	2	2	1.54	45	18.97453	1
287	2	2	20	2	2	1.73	46	15.36971	1
288	1	2	20	2	2	1.65	45	16.52893	1
289	1	1	20	1	1	1.56	49	20.13478	2
290	1	2	21	2	2	1.52	46	19.90997	1
291	1	1	20	1	1	1.62	47	17.90886	1
292	1	1	20	1	1	1.57	50	20.2848	2
293	1	1	20	1	1	1.49	40	18.01721	2
294	1	3	20	3	2	1.57	43	17.44493	1
295	1	4	20	4	2	1.49	43	19.3685	1
296	1	2	20	4	2	1.63	56	21.0772	2
297	2	1	20	1	1	1.48	46	21.00073	2
298	1	1	20	3	2	1.69	57	19.95728	2
299	2	4	20	4	2	1.71	90	30.7787	2

300	1	1	20	1	1	1.56	62.5	25.68212	1
301	1	3	20	3	2	1.61	48	18.5178	1
302	1	3	20	3	2	1.6	65	25.39063	1
303	1	4	20	3	2	1.52	52	22.50693	1
304	1	3	20	3	2	1.68	78	27.63605	1
305	1	2	20	4	2	1.63	51	19.1953	1
306	1	3	20	3	2	1.71	50	17.09928	1
307	1	1	20	1	1	1.54	46	19.39619	1
308	1	1	20	1	1	1.56	46	18.90204	1
309	2	3	20	3	2	1.75	75	24.4898	1
310	1	5	20	4	2	1.59	75	29.66655	1
311	1	3	20	3	2	1.6	49.8	19.45313	1
312	1	4	20	4	2	1.49	55	24.77366	1
313	2	2	20	4	2	1.68	74	26.21882	1
314	1	1	20	1	1	1.63	46	17.31341	1
315	1	1	20	1	1	1.56	62	25.47666	1
316	1	1	20	1	1	1.53	52	22.21368	2
317	1	1	20	1	1	1.58	58	23.23346	1
318	1	1	20	1	1	1.5	80	35.55556	1
319	1	1	20	1	1	1.57	45.4	18.4186	1
320	1	1	20	1	1	1.5	45	20	1
321	1	1	20	1	1	1.56	43	17.6693	1
322	1	1	20	1	1	1.62	73	27.81588	1
323	1	1	21	1	1	1.5	48	21.33333	1
324	1	1	20	1	1	1.53	40	17.08744	1
325	1	1	20	1	1	1.7	75	25.95156	1
326	1	2	20	2	2	1.58	53	21.23057	1
327	1	2	20	2	2	1.56	48	19.72387	1
328	1	2	20	2	2	1.57	43	17.44493	1
329	1	2	20	2	2	1.6	48	18.75	1
330	1	2	20	2	2	1.63	50	18.81892	1
331	2	1	20	1	1	1.7	62	21.45329	1
332	2	1	20	1	1	1.65	53	19.4674	1
333	1	2	20	2	2	1.68	68	24.09297	1
334	1	2	21	2	2	1.6	57	22.26563	1
335	1	2	21	2	2	1.61	50	19.28938	1
336	1	2	21	2	2	1.55	41	17.06556	1
337	2	2	20	2	2	1.77	50	15.95965	1
338	1	2	21	2	2	1.5	49	21.77778	1
339	1	2	21	2	2	1.62	61	23.24341	1
340	2	2	20	2	2	1.67	50	17.92822	1
341	1	4	20	2	2	1.59	48	18.98659	1
342	1	2	20	2	2	1.6	50	19.53125	1
343	1	4	21	2	2	1.62	44	16.76574	1
344	1	2	21	2	2	1.6	41	16.01563	1
345	1	1	21	1	1	1.64	49	18.21832	1
346	1	1	22	1	1	1.5	42	18.66667	2
347	1	1	21	1	1	1.65	48	17.63085	2
348	1	1	21	1	1	1.5	43	19.11111	2
349	1	1	21	1	1	1.45	45	21.40309	2

350	1	1	21	1	1	1.6	65	25.39063	2
351	1	1	21	1	1	1.5	50	22.22222	2
352	1	1	21	1	1	1.48	44	20.08766	1
353	1	2	21	2	2	1.6	52.6	20.54688	1
354	1	4	21	2	2	1.55	50	20.81165	1
355	1	4	22	2	2	1.6	62	24.21875	1
356	1	2	21	2	2	1.5	45	20	1
357	2	1	21	1	1	1.74	80	26.42357	1
358	1	2	21	2	2	1.67	63	22.58955	1
359	1	3	21	3	2	1.63	60.8	22.88381	1
360	1	1	21	1	1	1.57	57	23.12467	1
361	1	1	21	1	1	1.58	67	26.83865	1
362	1	4	21	2	2	1.58	48	19.22769	2
363	1	4	21	2	2	1.56	51	20.95661	1
364	1	1	21	1	1	1.54	48	20.2395	1
365	1	1	21	1	1	1.53	51	21.78649	2
366	1	1	21	1	1	1.44	54	26.04167	1
367	1	1	21	1	1	1.59	50	19.7777	1
368	1	1	22	1	1	1.61	85	32.79194	1
369	1	1	21	1	1	1.53	39	16.66026	2
370	1	1	21	1	1	1.51	64	28.06894	2
371	1	1	21	1	1	1.63	50	18.81892	1
372	1	1	21	1	1	1.69	57	19.95728	1
373	1	1	21	1	1	1.64	52	19.33373	2
374	1	2	21	2	2	1.67	53	19.00391	2
375	1	1	21	1	1	1.57	63	25.55885	2
376	1	1	21	1	1	1.5	44	19.55556	2
377	2	1	21	1	1	1.72	63	21.29529	2
378	2	1	20	1	1	1.8	60	18.51852	1
379	2	1	21	1	1	1.59	63	24.9199	1
380	1	1	21	1	1	1.58	49	19.62826	2
381	1	4	21	2	2	1.61	48	18.5178	2
382	2	2	22	2	2	1.86	73	21.10071	1
383	1	2	21	2	2	1.6	60	23.4375	1
384	1	1	21	1	1	1.5	41	18.22222	2
385	1	1	21	1	1	1.52	50	21.64127	2
386	1	1	21	1	1	1.55	45	18.73049	1
387	2	2	21	2	2	1.69	56	19.60716	1
388	2	2	21	2	2	1.8	74	22.83951	1
389	1	1	21	1	1	1.68	90	31.88776	2
390	1	1	21	1	1	1.51	47	20.61313	2
391	1	1	21	1	1	1.63	55	20.70082	1
392	1	1	21	1	1	1.54	48	20.2395	1
393	1	2	21	2	2	1.67	52	18.64534	1
394	1	2	21	2	2	1.68	55	19.48696	1
395	1	1	21	1	1	1.51	39	17.10451	1
396	1	2	21	2	2	1.65	48	17.63085	1
397	1	2	22	2	2	1.67	47	16.85252	1
398	2	2	21	2	2	1.86	60	17.34305	1
399	2	2	21	2	2	1.69	55	19.25703	1

400	2	2	20	2	2	1.62	50	19.05197	1
401	2	2	21	2	2	1.77	57	18.19401	1
402	2	1	20	1	1	1.69	55	19.25703	1
403	2	1	21	1	1	1.64	63	23.42356	1
404	2	5	21	2	2	1.67	65	23.30668	2
405	2	1	21	1	1	1.83	129	38.52011	1
406	2	1	20	1	1	1.6	60	23.4375	1
407	2	2	21	2	2	1.72	55	18.59113	1
408	2	2	21	2	2	1.7	49	16.95502	1
409	2	2	21	2	2	1.68	58	20.54989	1
410	1	2	19	2	2	1.64	55	20.44914	1
411	1	2	20	2	2	1.52	40	17.31302	1
412	2	1	19	1	1	1.6	89	34.76563	1
413	2	2	20	2	2	1.65	64	23.50781	1
414	1	2	20	2	2	1.65	46	16.89624	1
415	2	1	19	1	1	1.64	52	19.33373	1
416	2	1	19	1	1	1.64	60	22.30815	1
417	2	1	19	1	1	1.71	57	19.49318	1
418	2	1	19	1	1	1.65	55	20.20202	1
419	2	1	19	1	1	1.64	48	17.84652	1
420	2	1	20	1	1	1.63	50	18.81892	1
421	1	1	19	1	1	1.6	52	20.3125	1
422	1	1	19	1	1	1.6	48	18.75	1
423	2	2	20	2	2	1.73	70	23.38869	1
424	1	1	19	1	1	1.49	43	19.3685	1
425	2	2	20	2	2	1.8	90	27.77778	1
426	2	2	20	2	2	1.71	74	25.30693	1
427	2	2	19	2	2	1.78	54	17.0433	1
428	2	2	20	2	2	1.6	45	17.57813	1
429	2	1	19	1	1	1.7	55	19.03114	1
430	1	5	21	2	2	1.57	50	20.2848	1
431	1	1	20	1	1	1.62	59	22.48133	1
432	1	1	20	1	1	1.62	48.5	18.48041	1
433	2	1	20	1	1	1.67	50	17.92822	1
434	2	1	19	1	1	1.65	66	24.24242	1
435	1	1	20	1	1	1.69	75	26.25958	2
436	2	1	20	1	1	1.8	118	36.41975	2
437	2	1	20	1	1	1.7	66	22.83737	1
438	2	1	20	1	1	1.58	60	24.03461	2
439	2	1	20	1	1	1.8	60	18.51852	2
440	2	2	21	2	2	1.68	59	20.9042	1
441	1	2	21	2	2	1.5	43	19.11111	1
442	2	3	20	3	2	1.75	69	22.53061	1
443	2	2	20	2	2	1.61	52	20.06095	2
444	2	2	20	2	2	1.71	68	23.25502	1
445	1	2	21	2	2	1.72	52	17.57707	1
446	2	2	21	2	2	1.7	50	17.30104	1
447	2	2	21	2	2	1.75	51	16.65306	1
448	1	1	21	1	1	1.5	42	18.66667	2
449	1	1	20	1	1	1.62	48	18.28989	1

450	1	1	20	1	1	1.5	55	24.44444	1
451	2	2	21	2	2	1.73	66	22.05219	1
452	1	1	20	1	1	1.64	50	18.59012	1
453	2	1	20	1	1	1.68	80	28.34467	1
454	1	1	20	1	1	1.58	44	17.62538	2
455	2	2	21	2	2	1.64	55	20.44914	2
456	2	2	21	2	2	1.73	78	26.06168	2
457	2	2	21	2	2	1.71	75	25.64892	2
458	2	2	21	2	2	1.68	59	20.9042	1
459	2	2	21	2	2	1.7	60	20.76125	1
460	1	1	21	1	1	1.57	40	16.22784	2
461	2	2	22	2	2	1.76	96	30.99174	2
462	2	2	21	2	2	1.7	63	21.79931	1
463	1	1	21	1	1	1.6	86	33.59375	1
464	2	2	21	2	2	1.7	68	23.52941	1
465	2	2	21	2	2	1.66	57	20.68515	1
466	2	2	21	2	2	1.8	70	21.60494	1
467	2	2	21	2	2	1.76	72	23.2438	1
468	2	2	21	2	2	1.8	60	18.51852	1
469	2	2	21	2	2	1.65	65	23.87511	1
470	2	2	21	2	2	1.85	70	20.45289	2
471	2	1	20	1	1	1.8	50	15.4321	1
472	1	1	20	1	1	1.57	65	26.37024	1
473	2	1	20	1	1	1.8	70	21.60494	1
474	2	1	20	1	1	1.53	51	21.78649	1
475	2	1	20	1	1	1.7	64	22.14533	1
476	2	2	21	2	2	1.83	59	17.61773	1
477	2	2	21	2	2	1.65	53	19.4674	1
478	2	2	21	2	2	1.69	65	22.75831	1
479	2	2	21	2	2	1.69	58	20.30741	2
480	2	2	20	2	2	1.75	52	16.97959	1
481	2	1	20	1	1	1.65	85	31.2213	1
482	2	1	21	1	1	1.66	73	26.49151	1
483	1	1	21	1	1	1.53	60	25.63117	2
484	1	2	21	2	2	1.55	40	16.64932	1
485	2	2	21	2	2	1.667	80	28.78848	1
486	1	2	21	2	2	1.64	49	18.21832	1
487	1	1	20	1	1	1.48	60	27.39226	2
488	1	1	20	1	1	1.54	40	16.86625	1
489	1	1	20	1	1	1.56	65	26.7094	2
490	1	1	20	1	1	1.56	53	21.77844	2
491	1	2	21	2	2	1.62	55	20.95717	1
492	2	2	21	2	2	1.76	80	25.82645	1
493	1	1	21	1	1	1.62	52	19.81405	1
494	2	1	22	1	1	1.65	70	25.71166	1
495	1	1	21	1	1	1.62	42	16.00366	1
496	2	2	22	2	2	1.83	80	23.88844	1
497	2	2	21	2	2	1.74	65	21.46915	2
498	2	2	22	2	2	1.7	65	22.49135	1
499	2	2	22	2	2	1.79	61	19.03811	1

500	2	2	23	2	2	1.73	64	21.38394	2
501	2	1	21	4	2	1.65	60.5	22.22222	1
502	2	1	21	1	1	1.65	69	25.34435	1
503	1	1	22	1	1	1.62	68	25.91068	2
504	2	1	21	1	1	1.65	68	24.97704	1
505	1	2	22	2	2	1.58	45	18.02596	1
506	2	2	22	2	2	1.65	58	21.30395	1
507	1	5	22	2	2	1.54	60	25.29938	1
508	1	1	19	1	1	1.57	42	17.03923	1
509	2	2	19	2	2	1.69	45	15.75575	1
510	2	4	20	2	2	1.74	75	24.7721	1
511	1	2	20	2	2	1.59	53	20.96436	1
512	1	1	19	1	1	1.6	82	32.03125	1
513	1	1	24	1	1	1.52	95	41.11842	1
514	1	1	19	1	1	1.54	55	23.19109	1
515	1	4	10	4	2	1.55	55	22.89282	1
516	1	1	19	1	1	1.54	45	18.97453	1
517	1	1	19	1	1	1.54	44	18.55288	1
518	1	1	19	1	1	1.68	70	24.80159	1
519	1	1	19	1	1	1.57	94	38.13542	1
520	1	1	20	1	1	1.48	38	17.34843	1
521	2	4	20	2	2	1.73	78	26.06168	1
522	1	2	20	2	2	1.61	72	27.77671	1
523	1	4	19	3	2	1.57	56	22.71897	1
524	1	2	22	2	2	1.61	57	21.98989	1
525	1	1	19	1	1	1.58	50	20.02884	1
526	1	2	20	2	2	1.63	51	19.1953	1
527	1	2	20	2	2	1.64	50	18.59012	1
528	1	1	19	1	1	1.59	40	15.82216	1
529	2	2	20	2	2	1.65	75	27.54821	1
530	1	5	20	2	2	1.58	48	19.22769	1
531	1	2	20	2	2	1.62	52	19.81405	1
532	1	2	20	2	2	1.54	37	15.60128	1
533	2	2	20	2	2	1.78	70	22.09317	1
534	2	2	20	2	2	1.7	58	20.0692	1
535	1	2	20	2	2	1.54	53	22.34778	1
536	2	2	20	2	2	1.75	65	21.22449	1
537	2	1	22	1	1	1.67	48.5	17.39037	1
538	2	1	19	1	1	1.75	71	23.18367	1
539	2	1	19	1	1	1.62	58	22.10029	1
540	2	2	20	2	2	1.82	63	19.01944	1
541	2	2	20	2	2	1.71	47	16.07332	1
542	2	2	20	2	2	1.6	45	17.57813	1
543	2	2	20	2	2	1.75	85	27.7551	1
544	1	1	19	1	1	1.65	53	19.4674	1
545	1	1	19	1	1	1.51	39	17.10451	1
546	2	2	20	2	2	1.68	65	23.03005	1
547	1	4	20	2	2	1.58	51	20.42942	1
548	1	2	20	2	2	1.58	37	14.82134	1
549	2	2	20	2	2	1.65	50	18.36547	1

550	2	2	20	2	2	1.66	59	21.41094	1
551	2	2	20	2	2	1.65	45	16.52893	1
552	2	3	22	3	2	1.69	104	36.41329	2
553	2	1	19	1	1	1.63	46	17.31341	1
554	2	1	19	1	1	1.65	59	21.67126	1
555	1	1	20	1	1	1.59	48	18.98659	1
556	1	1	21	1	1	1.61	37	14.27414	1
557	2	2	21	2	2	1.8	70	21.60494	2
558	2	1	22	1	1	1.6	52	20.3125	1
559	1	1	21	1	1	1.56	43	17.6693	1
560	1	1	20	1	1	1.56	52	21.36752	1
561	1	1	21	1	1	1.57	40	16.22784	2
562	2	2	21	2	2	1.56	49	20.13478	1
563	2	4	21	2	2	1.7	55	19.03114	1
564	1	2	21	2	2	1.58	48	19.22769	1
565	2	2	21	2	2	1.72	70	23.66144	1
566	2	1	20	1	1	1.75	65	21.22449	1
567	2	2	21	2	2	1.83	76	22.69402	2
568	1	2	21	2	2	1.62	51	19.43301	1
569	2	2	21	2	2	1.75	51	16.65306	1
570	1	1	20	1	1	1.62	65	24.76757	1
571	2	4	21	2	2	1.79	72	22.47121	1
572	1	1	20	1	1	1.53	42	17.94182	1
573	1	1	20	1	1	1.56	45	18.49112	1
574	2	1	20	1	1	1.68	70	24.80159	1
575	1	1	20	1	1	1.64	63	23.42356	1
576	1	4	20	2	2	1.6	50	19.53125	2
577	2	1	21	1	1	1.66	45	16.33038	1
578	2	1	24	1	1	1.75	67	21.87755	1
579	1	2	23	2	2	1.7	56	19.37716	1
580	2	2	22	2	2	1.75	62	20.2449	1
581	1	2	22	2	2	1.53	46	19.65056	1
582	2	2	22	2	2	1.63	46.7	17.57688	1
583	2	2	22	2	2	1.75	58	18.93878	1
584	2	4	22	2	2	1.79	55	17.16551	1
585	2	3	21	3	2	1.77	85	27.13141	1
586	1	3	24	3	2	1.54	45	18.97453	1
587	2	2	22	2	2	1.93	82	22.01401	1
588	1	2	22	2	2	1.51	50	21.92886	1
589	2	2	23	2	2	1.76	75	24.21229	2
590	1	1	21	1	1	1.58	45	18.02596	1
591	1	1	21	1	1	1.6	43	16.79688	2
592	1	1	21	1	1	1.54	50	21.08281	2
593	1	1	22	1	1	1.57	70	28.39872	2
594	1	1	21	1	1	1.65	49	17.99816	1
595	2	2	24	2	2	1.8	54	16.66667	1
596	2	2	22	2	2	1.78	53	16.72769	1
597	2	5	22	2	2	1.58	80	32.04615	1
598	1	1	21	1	1	1.53	45	19.22338	2
599	1	1	21	1	1	1.55	60	24.97399	2

600	1	1	22	1	1	1.63	54	20.32444	1
601	1	1	21	1	1	1.63	78	29.35752	1
602	1	2	20	2	2	1.58	42	16.82423	1
603	1	2	20	2	2	1.5	42	18.66667	1
604	1	2	20	2	2	1.59	49	19.38214	1
605	2	4	20	2	2	1.6	62.3	24.33594	1
606	1	4	20	2	2	1.65	55	20.20202	1
607	1	2	19	2	2	1.63	51	19.1953	1
608	2	2	19	2	2	1.67	72	25.81663	1
609	1	1	19	1	1	1.56	47	19.31295	1
610	1	2	20	4	2	1.65	55	20.20202	1
611	1	1	19	1	1	1.45	39	18.54935	1
612	1	2	19	2	2	1.67	50	17.92822	1
613	1	2	20	2	2	1.6	46	17.96875	1
614	1	1	19	1	1	1.62	56	21.33821	1
615	1	1	19	1	1	1.62	47	17.90886	1
616	1	2	20	2	2	1.66	55	19.95936	1
617	1	2	19	4	2	1.62	80	30.48316	1
618	1	2	20	2	2	1.57	49	19.8791	1
619	1	2	20	2	2	1.58	46	18.42653	1
620	2	2	20	2	2	1.69	58	20.30741	1
621	2	2	20	2	2	1.8	89	27.46914	1
622	1	1	20	1	1	1.61	46	17.74623	1
623	1	1	20	1	1	1.6	50	19.53125	1
624	1	1	19	1	1	1.52	35	15.14889	1
625	1	1	19	1	1	1.52	50	21.64127	1
626	1	1	19	1	1	1.68	50	17.71542	1
627	1	1	20	1	1	1.55	43	17.89802	1
628	2	4	19	2	2	1.62	55	20.95717	1
629	1	2	20	2	2	1.62	52	19.81405	1
630	1	1	19	1	1	1.59	44	17.40437	1
631	1	2	20	2	2	1.64	65	24.16716	1
632	2	2	22	2	2	1.73	60	20.04745	1
633	1	2	19	2	2	1.6	52	20.3125	1
634	1	2	19	2	2	1.51	62	27.19179	1
635	2	1	19	1	1	1.81	78	23.8088	1
636	2	4	20	2	2	1.73	70	23.38869	1
637	2	2	20	2	2	1.83	66	19.70796	1
638	2	2	20	2	2	1.73	50	16.7062	1
639	2	1	19	1	1	1.63	43	16.18427	1
640	1	2	20	4	2	1.55	57	23.72529	1
641	2	2	19	2	2	1.74	75	24.7721	1
642	1	2	20	2	2	1.65	59	21.67126	1
643	1	2	20	2	2	1.58	47	18.82711	1
644	2	1	19	1	1	1.7	55	19.03114	1
645	1	1	22	1	1	1.56	48	19.72387	1
646	1	1	20	1	1	1.53	54	23.06805	1
647	1	2	20	2	2	1.63	48	18.06617	1
648	1	2	21	2	2	1.57	45	18.25632	1
649	1	2	20	2	2	1.6	48	18.75	1

650	2	2	20	2	2	1.61	48	18.5178	1
651	2	2	20	2	2	1.65	118	43.34252	1
652	2	2	20	2	2	1.76	58	18.72417	1
653	1	1	19	1	1	1.53	50	21.35931	1
654	1	1	19	1	1	1.54	35	14.75797	1
655	2	1	20	1	1	1.71	93	31.80466	1
656	2	4	19	3	2	1.63	85	31.99217	1
657	2	3	19	3	2	1.75	63	20.57143	1
658	1	4	19	2	2	1.57	40	16.22784	1
659	1	2	20	2	2	1.65	55	20.20202	1
660	1	2	20	2	2	1.52	38	16.44737	1
661	1	2	20	2	2	1.63	53	19.94806	1
662	1	2	20	2	2	1.7	53	18.3391	2
663	1	2	20	2	2	1.67	40	14.34257	1
664	1	4	20	2	2	1.61	50	19.28938	1
665	1	2	20	2	2	1.63	45	16.93703	1
666	1	2	20	2	2	1.65	43	15.79431	1
667	1	2	20	2	2	1.68	61	21.61281	1
668	1	2	19	2	2	1.54	52	21.92613	1
669	1	1	19	1	1	1.52	56	24.23823	1
670	1	1	19	1	1	1.46	40	18.76525	1
671	1	2	20	4	2	1.68	46	16.29819	1
672	1	3	20	3	2	1.61	50	19.28938	1
673	1	2	20	2	2	1.58	40	16.02307	1
674	1	2	20	2	2	1.53	44	18.79619	1
675	1	2	20	2	2	1.55	42	17.48179	1
676	1	2	21	2	2	1.63	53	19.94806	1
677	1	2	20	2	2	1.52	47	20.3428	1
678	2	2	20	2	2	1.69	72	25.2092	1
679	2	4	21	2	2	1.78	79	24.93372	1
680	1	1	22	1	1	1.54	55	23.19109	1
681	1	2	21	2	2	1.68	53	18.77834	1
682	1	2	21	2	2	1.66	60	21.77384	1
683	1	2	21	2	2	1.58	46	18.42653	1
684	1	2	21	2	2	1.6	52	20.3125	1
685	1	1	21	1	1	1.56	63	25.88757	1
686	1	1	21	1	1	1.61	49	18.90359	1
687	1	1	21	2	2	1.62	44	16.76574	1
688	1	1	21	1	1	1.54	57	24.03441	1
689	1	1	21	1	1	1.46	37.5	17.59242	1
690	1	2	21	2	2	1.72	52	17.57707	1
691	1	2	21	2	2	1.65	45	16.52893	1
692	1	2	20	2	2	1.58	78	31.24499	2
693	1	2	21	2	2	1.55	48	19.97919	1
694	1	2	21	2	2	1.69	47	16.45601	1
695	1	1	21	1	1	1.58	90	36.05191	1
696	1	1	21	1	1	1.53	49	20.93212	1
697	1	2	21	2	2	1.62	46	17.52782	1
698	2	2	21	2	2	1.69	63	22.05805	1
699	1	1	20	3	2	1.53	42	17.94182	2

700	2	1	20	1	1	1.52	60	25.96953	1
701	1	5	21	4	2	1.71	65	22.22906	1
702	1	2	21	2	2	1.6	46	17.96875	1
703	1	3	21	3	2	1.49	40	18.01721	1
704	1	2	21	2	2	1.6	50	19.53125	1
705	1	4	21	2	2	1.6	58	22.65625	1
706	1	2	21	2	2	1.59	50	19.7777	1
707	1	2	21	2	2	1.53	60	25.63117	1
708	1	2	21	2	2	1.58	40	16.02307	1
709	1	1	23	1	1	1.55	47	19.56296	1
710	1	2	21	2	2	1.5	43	19.11111	1
711	2	1	25	1	1	1.75	90	29.38776	1
712	2	1	20	1	1	1.5	68	30.22222	1
713	2	1	21	1	1	1.67	73	26.17519	1
714	2	2	21	2	2	1.77	67	21.38594	2
715	1	5	21	2	2	1.6	47	18.35938	2
716	1	1	21	1	1	1.58	62	24.83576	1
717	1	1	21	1	1	1.5	55	24.44444	1
718	1	1	21	1	1	1.5	68	30.22222	1
719	1	1	22	1	1	1.5	50	22.22222	1
720	1	1	20	1	1	1.63	60	22.58271	2
721	1	2	21	2	2	1.65	56	20.56933	1
722	1	1	20	1	1	1.58	47	18.82711	1
723	1	2	20	2	2	1.59	40	15.82216	2
724	1	2	20	2	2	1.56	50	20.54569	1
725	1	2	20	2	2	1.65	46	16.89624	1
726	1	1	20	1	1	1.67	61	21.87242	1
727	1	2	20	2	2	1.64	50	18.59012	1
728	1	2	19	2	2	1.52	42	18.17867	1
729	1	1	19	1	1	1.53	53	22.64086	1
730	1	2	20	2	2	1.59	56	22.15102	1
731	1	2	20	2	2	1.55	52	21.64412	1
732	1	1	19	1	1	1.58	55	22.03173	1
733	1	1	19	1	1	1.58	60	24.03461	1
734	1	2	20	2	2	1.54	40	16.86625	1
735	2	4	21	2	2	1.78	70	22.09317	2
736	1	2	22	2	2	1.63	45	16.93703	1
737	1	2	21	2	2	1.58	53	21.23057	1
738	1	2	21	2	2	1.6	48	18.75	2
739	1	2	21	2	2	1.55	55	22.89282	1
740	2	2	21	2	2	1.65	53	19.4674	1
741	1	2	22	2	2	1.63	58	21.82995	1
742	1	2	22	2	2	1.62	48	18.28989	1
743	1	2	21	2	2	1.63	47	17.68979	1
744	1	2	21	2	2	1.66	46	16.69328	1
745	1	1	21	3	2	1.57	53	21.50189	2
746	2	1	22	1	1	1.7	68	23.52941	1
747	1	3	21	3	2	1.66	53	19.23356	1
748	2	2	21	2	2	1.69	57	19.95728	1
749	1	1	21	1	1	1.54	71	29.93759	1

750	1	1	21	1	1	1.6	57	22.26563	1
751	1	1	24	1	1	1.59	60	23.73324	1
752	1	1	21	1	1	1.48	57	26.02264	1
753	1	1	21	1	1	1.52	38	16.44737	2
754	1	1	21	1	1	1.63	55	20.70082	2
755	2	1	22	1	1	1.63	50	18.81892	1
756	2	2	21	2	2	1.67	50	17.92822	2
757	2	2	21	2	2	1.67	52	18.64534	2
758	1	2	22	2	2	1.53	43	18.369	1
759	1	2	21	2	2	1.56	50	20.54569	1
760	2	2	21	2	2	1.69	53	18.55677	1
761	1	2	21	2	2	1.6	58	22.65625	1
762	1	2	21	2	2	1.65	54	19.83471	2
763	1	3	20	3	2	1.53	44	18.79619	1
764	1	2	20	2	2	1.63	50	18.81892	1
765	1	1	19	1	1	1.56	48	19.72387	1
766	1	1	19	1	1	1.56	43	17.6693	1
767	1	1	19	1	1	1.61	47	18.13202	1
768	1	1	19	1	1	1.57	70	28.39872	1
769	1	1	19	1	1	1.57	47	19.06771	1
770	1	1	23	1	1	1.61	57	21.98989	1
771	2	1	24	1	1	1.73	77	25.72756	2
772	2	1	20	1	1	1.65	55	20.20202	1
773	2	5	21	2	2	1.7	70	24.22145	1
774	2	2	21	2	2	1.74	65.6	21.66733	1
775	2	2	21	2	2	1.61	75	28.93407	2
776	2	2	20	2	2	1.65	65	23.87511	1
777	2	2	22	2	2	1.76	90	29.05475	1
778	2	1	19	1	1	1.76	63	20.33833	1
779	1	1	19	1	1	1.59	53	20.96436	1
780	1	1	21	1	1	1.5	50	22.22222	1
781	1	1	21	1	1	1.6	52	20.3125	1
782	1	1	21	1	1	1.47	44	20.36189	2
783	2	1	20	1	1	1.86	80	23.12406	1
784	1	1	22	1	1	1.6	64	25	2
785	2	4	21	2	2	1.8	83	25.61728	1
786	2	2	22	2	2	1.85	57	16.65449	1
787	2	2	22	2	2	1.74	88	29.06593	1
788	1	1	20	1	1	1.51	61	26.75321	1

fh	personal	smoking	exposure	recexpose	eff	effirec	intent	RF	Diet
2	2	2	1	1	1	4	9	2	4
1	2	2	3	2	1	4	5	2	4
2	2	2	1	1	2	3	9	4	4
1	2	2	1	1	2	3	9	3	4
2	2	2	1	1	1	4	4	4	3
1	2	1	1	1	2	3	7	2	3
2	2	2	1	1	1	4	6	3	5
3	2	2	1	1	1	4	7	3	4
2	2	2	2	2	4	1	9	1	2
3	2	1	1	1	2	3	9	2	2
1	2	2	1	1	1	4	8	3	6
2	2	2	1	1	1	4	8	3	2
2	2	2	1	1	1	4	8	2	4
2	2	2	1	1	1	4	7	2	3
2	2	2	1	1	3	2	6	4	3
2	1	2	1	1	1	4	9	2	2
1	2	2	2	2	1	4	9	4	6
2	2	2	1	1	1	4	9	3	3
1	2	2	1	1	1	4	7	3	6
2	2	2	1	1	3	2	5	1	1
2	2	2	2	2	1	4	9	3	4
2	2	2	1	1	2	3	7	2	2
2	1	2	1	1	1	4	6	4	3
2	2	2	1	1	1	4	5	5	3
3	2	2	1	1	2	3	5	3	4
2	2	2	1	1	2	3	9	4	6
2	2	2	1	1	2	3	8	3	3
3	2	2	1	1	1	4	7	2	5
3	2	2	1	1	2	3	9	3	3
2	2	2	1	1	1	4	7	1	2
2	2	2	1	1	2	3	9	3	2
2	2	2	2	2	1	4	9	4	3
2	2	2	1	1	1	4	9	3	5
1	1	2	1	1	1	4	9	5	6
2	2	2	1	1	1	4	7	3	4
2	2	2	2	2	1	4	9	4	4
3	2	2	1	1	1	4	7	2	5
2	2	2	1	1	1	4	9	2	6
2	2	2	1	1	1	4	9	3	5
2	2	2	2	2	2	3	8	4	5
2	2	2	1	1	2	3	7	5	6
2	2	2	1	1	1	4	9	3	5
2	2	2	1	1	2	3	9	5	6
2	2	2	1	1	1	4	8	4	4
2	2	2	2	2	1	4	7	3	1
2	2	2	1	1	1	4	9	4	3
2	2	2	1	1	1	4	9	5	6
2	2	2	1	1	1	4	9	2	2
2	2	2	2	2	1	4	5	5	5

2	2	2	1	1	1	4	9	3	5
2	2	2	1	1	2	3	8	3	3
2	2	2	1	1	3	2	9	4	3
1	2	2	1	1	1	4	8	2	4
2	2	2	1	1	2	3	6	3	5
2	2	2	1	1	1	4	7	4	6
3	2	2	1	1	3	2	7	3	4
3	2	2	1	1	1	4	8	3	6
2	2	2	1	1	2	3	7	4	3
2	2	2	1	1	1	4	9	2	4
2	2	2	1	1	1	4	7	3	5
2	2	2	1	1	2	3	5	5	6
2	2	2	1	1	1	4	7	2	3
1	2	2	1	1	1	4	9	2	3
2	2	2	1	1	2	3	9	2	4
2	2	2	1	1	2	3	8	3	1
2	2	2	1	1	2	3	4	2	3
2	2	2	3	2	1	4	8	3	4
2	2	2	1	1	2	3	8	0	5
2	3	2	3	2	2	3	9	4	2
2	2	2	2	2	2	3	7	2	3
2	2	2	1	1	2	3	7	3	3
1	2	2	1	1	1	4	7	5	5
2	1	2	1	1	1	4	7	2	4
2	2	2	3	2	1	4	9	2	5
2	2	2	2	2	2	3	5	2	2
2	2	2	1	1	2	3	9	4	4
2	2	2	3	2	2	3	6	1	5
2	2	2	3	2	1	4	9	3	3
2	2	2	2	2	1	4	9	4	3
2	2	2	3	2	1	4	9	4	3
1	2	2	3	2	1	4	8	3	6
2	2	2	2	2	1	4	6	3	5
1	2	2	2	2	1	4	6	3	5
2	2	2	1	1	2	3	9	4	4
2	2	2	1	1	3	2	9	2	4
2	2	2	1	1	1	4	7	2	3
3	2	2	2	2	1	4	9	3	3
2	2	2	2	2	1	4	9	3	4
2	1	2	1	1	1	4	6	2	5
2	1	2	1	1	2	3	7	4	4
1	3	2	1	1	4	1	5	3	6
2	2	2	1	1	2	3	8	4	4
3	2	2	1	1	1	4	9	2	4
2	2	2	2	2	2	3	9	1	4
3	2	2	2	2	2	3	9	3	3
3	2	2	1	1	1	4	9	2	3
1	2	2	1	1	1	4	7	1	2
2	2	2	1	1	1	4	9	2	3
2	2	2	1	1	1	4	7	2	1

2	2	2	1	1	1	4	8	2	4
3	2	2	1	1	2	3	9	1	3
3	1	2	1	1	1	4	9	2	4
2	2	2	2	2	1	4	9	3	5
2	2	2	1	1	1	4	7	4	2
2	2	2	1	1	1	4	9	3	6
2	2	2	1	1	1	4	9	2	0
2	2	2	1	1	1	4	9	2	1
2	2	2	2	2	1	4	5	4	4
2	2	2	1	1	1	4	9	4	6
1	1	2	3	2	1	4	8	3	6
2	2	2	1	1	2	3	9	2	5
1	2	2	1	1	1	4	2	3	3
2	2	2	2	2	1	4	9	4	2
2	2	2	1	1	1	4	7	3	3
3	3	2	2	2	1	4	7	2	4
1	2	2	1	1	1	4	8	5	4
2	2	2	1	1	1	4	8	2	2
2	2	2	1	1	2	3	8	1	1
2	1	2	2	2	1	4	8	3	4
2	2	2	2	2	1	4	9	2	4
2	2	2	2	2	2	3	7	2	3
2	2	2	2	2	1	4	8	2	1
2	2	2	1	1	1	4	8	4	3
2	2	2	3	2	2	3	7	4	2
2	2	2	3	2	1	4	8	0	4
3	3	2	1	1	1	4	8	1	1
2	2	2	1	1	2	3	7	4	3
2	2	2	3	2	1	4	9	4	6
3	2	2	1	1	1	4	9	3	5
3	2	2	1	1	1	4	9	3	4
2	2	2	1	1	1	4	5	3	0
2	2	2	2	2	1	4	8	0	1
2	2	2	1	1	2	3	7	3	4
2	2	2	2	2	1	4	9	5	5
2	2	2	1	1	1	4	7	5	5
1	3	2	1	1	1	4	8	4	5
2	2	2	1	1	2	3	9	2	6
2	2	2	3	2	2	3	6	4	4
2	2	2	1	1	1	4	7	3	5
2	2	2	3	2	1	4	8	2	5
1	2	2	1	1	1	4	9	2	3
2	2	2	2	2	1	4	7	2	4
2	2	2	1	1	1	4	8	4	4
2	2	2	1	1	1	4	9	4	4
2	2	2	1	1	2	3	7	3	4
2	2	2	1	1	1	4	9	5	3
2	2	2	1	1	1	4	7	4	4
2	2	2	1	1	1	4	7	1	2
1	2	1	1	1	3	2	8	1	2

2	1	2	1	1	3	2	6	2	3
2	2	2	1	1	1	4	9	3	3
2	2	2	1	1	1	4	9	2	6
2	2	2	1	1	1	4	9	2	6
2	2	2	1	1	1	4	9	2	6
2	2	2	2	2	1	4	9	2	4
2	2	2	2	2	1	4	8	4	5
2	2	2	1	1	1	4	9	4	5
1	2	2	1	1	1	4	9	4	5
1	2	2	1	1	1	4	5	4	4
2	2	2	1	1	2	3	5	1	3
2	2	1	2	2	1	4	7	4	5
2	2	2	2	2	2	3	7	5	4
2	2	2	1	1	1	4	8	3	4
3	2	2	3	2	2	3	9	5	5
2	2	2	2	2	2	3	8	2	4
3	2	2	1	1	1	4	6	3	5
2	2	2	1	1	1	4	7	3	6
2	2	2	2	2	1	4	9	3	4
2	2	2	2	2	1	4	8	3	3
3	2	2	1	1	1	4	9	3	3
2	2	2	2	2	2	3	9	2	5
2	2	2	3	2	2	3	7	5	6
2	2	2	1	1	1	4	7	3	3
3	2	2	2	2	2	3	7	4	5
2	2	2	1	1	1	4	8	4	3
2	2	2	2	2	1	4	9	0	2
1	2	2	1	1	1	4	9	3	4
2	2	2	1	1	1	4	9	3	4
2	2	2	1	1	1	4	9	4	4
1	2	2	3	2	1	4	9	3	5
2	2	2	1	1	1	4	9	3	3
2	2	2	1	1	2	3	8	5	6
2	2	2	2	2	1	4	8	2	4
3	2	2	1	1	1	4	9	4	4
2	2	2	1	1	1	4	9	3	4
2	2	2	1	1	1	4	8	5	4
2	2	2	1	1	1	4	8	4	4
2	2	2	1	1	1	4	7	4	4
2	2	2	1	1	1	4	9	3	5
2	2	2	1	1	1	4	8	4	4
2	2	2	1	1	1	4	9	3	3
2	2	2	3	2	2	3	7	3	5
1	2	2	1	1	2	3	8	0	2
2	2	2	1	1	1	4	9	3	4
2	2	2	1	1	1	4	7	2	5
2	3	2	1	1	1	4	7	4	3
2	2	2	2	2	1	4	9	5	4
2	2	2	1	1	1	4	7	1	0
2	2	2	1	1	2	3	6	2	3

1	2	2	1	1	1	4	6	0	1
3	2	2	2	2	2	3	7	3	5
2	2	2	2	2	1	4	9	4	5
3	2	2	1	1	1	4	8	5	2
1	2	2	1	1	1	4	9	3	5
2	2	2	1	1	2	3	9	2	4
3	2	2	1	1	2	3	5	4	4
2	2	2	1	1	1	4	9	3	4
2	2	2	1	1	2	3	5	4	3
3	2	2	1	1	1	4	8	4	4
2	2	2	2	2	1	4	9	4	5
3	2	2	1	1	1	4	9	4	5
2	2	2	3	2	2	3	7	3	5
2	2	2	1	1	1	4	5	3	3
2	2	2	1	1	1	4	8	3	5
2	2	2	1	1	1	4	9	4	5
3	2	2	1	1	2	3	8	4	6
2	2	2	2	2	1	4	6	2	6
2	2	1	3	2	2	3	8	3	4
3	2	2	1	1	2	3	9	0	6
1	2	2	1	1	2	3	7	2	1
1	2	2	1	1	2	3	7	3	5
2	2	2	1	1	2	3	8	2	4
1	2	2	2	2	1	4	5	4	5
2	2	2	1	1	1	4	9	3	4
1	2	2	1	1	2	3	6	3	3
2	2	2	3	2	1	4	7	3	2
2	2	2	1	1	3	2	4	3	5
3	2	2	1	1	2	3	6	4	6
1	2	2	1	1	2	3	8	3	3
1	2	2	1	1	1	4	9	2	4
2	2	2	1	1	1	4	6	4	4
2	2	2	3	2	2	3	5	5	4
3	2	2	1	1	1	4	6	3	3
2	2	2	2	2	2	3	7	4	4
3	2	2	1	1	1	4	9	4	4
2	2	2	1	1	3	2	9	4	5
2	2	2	1	1	4	1	9	4	5
2	2	2	2	2	2	3	6	4	5
2	2	2	1	1	1	4	9	3	3
2	2	2	2	2	2	3	7	4	4
2	2	2	1	1	1	4	7	3	3
2	2	2	1	1	2	3	9	3	4
1	2	2	1	1	2	3	6	2	4
1	2	2	1	1	1	4	6	3	4
2	2	2	1	1	1	4	9	4	5
2	2	2	1	1	1	4	8	3	3
2	2	2	1	1	4	1	9	2	4
1	2	1	1	1	1	4	9	1	1
2	2	2	2	2	2	3	5	5	3

2	2	2	1	1	2	3	5	4	3
2	2	2	2	2	2	3	5	5	3
2	2	2	2	2	2	3	6	5	4
2	2	2	2	2	2	3	9	4	3
2	2	2	1	1	1	4	9	4	1
2	2	2	1	1	1	4	9	4	2
2	2	2	1	1	1	4	9	1	4
3	2	2	1	1	2	3	9	4	4
2	2	2	1	1	1	4	6	2	5
1	2	2	1	1	1	4	4	2	4
2	2	2	2	2	1	4	8	0	2
2	2	1	1	1	3	2	9	2	3
2	2	2	1	1	1	4	7	0	4
2	2	2	2	2	2	3	5	2	3
2	2	2	3	2	4	1	7	4	4
1	2	1	1	1	1	4	5	3	4
2	2	2	1	1	2	3	9	4	3
2	2	2	2	2	2	3	9	3	5
2	2	2	1	1	2	3	7	3	4
2	2	2	1	1	1	4	9	2	5
1	2	2	1	1	1	4	7	4	5
2	2	2	1	1	2	3	7	3	5
2	2	1	1	1	2	3	8	2	3
2	2	2	1	1	1	4	9	3	6
2	2	2	1	1	2	3	8	3	5
2	2	2	1	1	2	3	8	4	4
1	2	2	1	1	1	4	3	4	3
2	2	2	2	2	1	4	3	2	6
2	2	2	1	1	1	4	9	3	2
2	2	2	1	1	1	4	9	3	4
2	2	2	1	1	1	4	5	3	4
1	2	2	2	2	1	4	5	3	2
3	2	2	1	1	1	4	7	3	2
2	2	2	1	1	1	4	9	3	3
2	2	2	1	1	4	1	7	4	4
2	2	2	1	1	1	4	8	0	4
2	2	2	1	1	2	3	6	1	4
2	2	2	1	1	2	3	9	4	5
2	2	2	2	2	2	3	9	3	2
1	2	2	1	1	2	3	8	3	4
1	2	2	1	1	1	4	9	3	4
2	2	2	1	1	1	4	9	2	5
2	2	2	1	1	3	2	9	2	6
2	2	2	1	1	1	4	7	1	5
3	2	2	1	1	1	4	4	2	6
3	2	2	1	1	1	4	8	4	3
2	2	2	1	1	1	4	9	3	6
2	1	2	2	2	1	4	9	4	4
2	2	2	1	1	2	3	5	3	5
1	2	2	1	1	1	4	7	3	6

1	2	2	1	1	1	4	9	4	6
2	2	2	1	1	2	3	9	4	2
2	2	2	1	1	1	4	8	3	4
2	2	2	1	1	2	3	7	4	5
2	2	2	3	2	1	4	5	3	3
2	2	1	1	1	1	4	8	5	3
2	2	2	1	1	2	3	8	3	4
1	2	2	1	1	1	4	9	4	3
2	2	2	1	1	1	4	8	3	5
2	2	2	1	1	2	3	9	3	5
1	2	2	1	1	1	4	8	3	4
2	2	2	1	1	1	4	9	2	6
2	2	2	1	1	2	3	9	5	5
1	2	2	1	1	1	4	9	2	6
2	2	2	1	1	2	3	9	3	5
1	2	2	1	1	1	4	8	4	5
1	2	2	1	1	2	3	8	2	5
2	2	2	1	1	2	3	9	4	5
2	2	2	1	1	1	4	7	2	4
3	2	2	2	2	1	4	9	1	4
2	2	2	1	1	1	4	9	2	3
2	2	2	1	1	2	3	8	4	5
2	2	2	1	1	2	3	9	3	5
2	2	2	1	1	1	4	9	2	4
1	2	2	1	1	1	4	5	2	4
2	2	2	1	1	1	4	9	3	6
2	2	2	1	1	1	4	8	5	5
2	2	2	1	1	2	3	9	5	5
1	2	2	1	1	1	4	7	4	4
3	2	2	2	2	1	4	8	5	4
2	2	2	1	1	1	4	8	2	5
2	2	2	1	1	2	3	9	4	4
1	2	2	1	1	1	4	9	3	5
2	2	2	1	1	1	4	5	3	3
1	2	2	1	1	2	3	9	3	5
1	2	2	1	1	1	4	5	3	5
2	2	2	1	1	1	4	7	4	4
2	2	2	1	1	3	2	7	4	6
2	2	2	1	1	2	3	6	3	4
2	2	2	1	1	1	4	9	4	5
2	2	2	1	1	1	4	9	4	5
2	2	2	1	1	2	3	6	4	6
2	2	2	1	1	3	2	7	4	5
1	3	2	1	1	3	2	7	2	5
1	2	2	1	1	2	3	9	4	5
3	2	2	1	1	1	4	8	3	5
2	2	2	2	2	1	4	7	4	4
2	2	2	2	2	1	4	9	5	6
2	2	1	1	1	1	4	9	3	3
2	2	2	1	1	1	4	7	3	3

1	2	2	1	1	1	4	9	3	4
2	2	2	2	2	1	4	9	3	6
1	2	2	1	1	1	4	8	4	6
3	2	2	1	1	2	3	6	5	6
2	2	2	2	2	2	3	6	2	5
1	2	2	1	1	1	4	8	3	5
2	2	2	1	1	1	4	8	5	3
1	2	2	1	1	1	4	7	5	5
2	2	2	1	1	2	3	9	3	6
1	2	2	1	1	1	4	9	4	4
1	2	1	1	1	1	4	9	4	4
2	2	2	1	1	1	4	9	4	4
2	2	2	2	2	2	3	7	3	2
2	2	2	2	2	1	4	9	2	4
2	2	1	1	1	1	4	9	4	5
2	2	2	1	1	1	4	9	2	5
3	2	2	2	2	1	4	8	3	6
2	2	2	1	1	1	4	9	3	4
2	2	2	1	1	1	4	9	3	6
2	2	2	1	1	1	4	9	3	5
1	2	2	1	1	1	4	9	4	5
3	2	2	1	1	1	4	9	3	3
3	2	2	1	1	1	4	9	4	3
2	2	2	2	2	1	4	9	3	2
2	2	2	1	1	2	3	9	3	2
1	2	2	1	1	1	4	9	3	5
2	2	2	1	1	1	4	7	2	4
2	2	2	1	1	1	4	9	5	1
1	2	1	3	2	1	4	9	4	0
1	2	2	2	2	2	3	7	2	5
2	2	2	1	1	2	3	8	4	6
1	2	1	2	2	1	4	8	5	5
2	1	1	2	2	3	2	5	3	3
2	2	2	1	1	1	4	9	3	5
1	2	2	1	1	2	3	4	2	4
2	2	2	2	2	1	4	9	4	4
2	2	2	1	1	1	4	8	5	4
2	2	2	1	1	1	4	9	5	5
2	2	2	2	2	1	4	9	3	5
2	2	2	1	1	3	2	5	5	6
2	2	2	2	2	2	3	9	3	3
2	2	2	2	2	1	4	9	4	4
2	2	2	1	1	1	4	9	4	4
2	2	2	1	1	2	3	7	5	5
2	2	2	2	2	1	4	9	4	5
1	2	2	1	1	1	4	7	3	4
2	2	2	1	1	1	4	9	4	4
2	2	2	2	2	1	4	9	4	6
2	2	2	1	1	1	4	9	4	6
2	2	2	1	1	2	3	9	4	5

2	2	2	1	1	3	2	8	2	3
2	2	2	2	2	2	3	5	3	5
3	2	2	1	1	2	3	6	4	4
2	2	2	2	2	3	2	6	1	5
2	2	2	2	2	3	2	5	1	3
2	2	1	1	1	2	3	7	2	3
2	2	1	1	1	2	3	9	1	5
2	2	2	2	2	2	3	9	3	2
2	2	2	3	2	1	4	7	3	2
2	2	2	1	1	2	3	6	3	2
3	2	2	1	1	2	3	8	4	6
2	2	2	1	1	1	4	6	3	6
3	2	2	3	2	1	4	8	3	4
1	2	2	1	1	1	4	8	3	5
1	2	2	1	1	2	3	5	4	4
2	2	1	2	2	1	4	7	1	5
2	2	2	1	1	1	4	8	3	3
2	2	1	1	1	1	4	1	0	1
2	2	2	1	1	1	4	9	2	2
2	2	2	2	2	1	4	9	3	4
2	2	2	1	1	2	3	9	2	2
2	2	2	1	1	2	3	9	3	5
3	2	2	2	2	2	3	8	4	5
2	2	1	1	1	4	1	9	3	4
3	2	2	2	2	4	1	9	2	3
3	3	2	1	1	1	4	9	3	3
2	1	2	2	2	1	4	6	5	4
3	2	2	1	1	1	4	3	5	6
2	2	2	1	1	1	4	9	5	4
2	1	1	1	1	1	4	7	3	5
2	2	2	1	1	1	4	7	4	5
2	2	2	2	2	2	3	6	5	5
2	2	2	2	2	1	4	5	3	4
2	2	2	1	1	3	2	7	1	2
1	2	2	1	1	3	2	5	3	4
2	2	2	1	1	1	4	8	2	2
2	2	1	1	1	3	2	7	4	5
2	2	2	3	2	2	3	9	2	4
2	2	1	2	2	3	2	7	2	2
2	2	2	1	1	2	3	7	2	3
2	2	2	1	1	1	4	5	4	4
2	2	2	1	1	3	2	5	3	4
2	2	2	2	2	1	4	9	4	4
2	2	2	1	1	3	2	6	2	5
2	2	2	1	1	1	4	9	4	5
2	2	2	1	1	1	4	9	3	4
2	2	2	2	2	3	2	6	2	1
2	2	2	2	2	3	2	2	2	4
2	2	1	3	2	1	4	9	2	3
2	2	2	2	2	1	4	9	2	4

3	2	2	1	1	2	3	8	4	3
2	2	2	1	1	2	3	7	2	4
2	2	2	2	2	1	4	8	5	6
2	2	1	1	1	1	4	9	5	5
1	2	2	3	2	1	4	7	3	5
2	2	2	1	1	2	3	6	3	6
2	2	2	2	2	1	4	7	1	4
2	2	2	1	1	2	3	7	4	5
2	2	2	1	1	2	3	6	3	5
2	2	2	2	2	2	3	8	4	3
2	2	2	1	1	2	3	4	4	3
2	2	2	1	1	4	1	6	3	6
2	2	2	2	2	1	4	7	4	6
1	2	2	1	1	1	4	9	4	3
2	2	2	1	1	2	3	7	3	5
2	2	2	1	1	1	4	5	5	3
2	2	2	2	2	1	4	8	4	4
1	2	2	3	2	2	3	5	3	5
2	2	2	1	1	2	3	8	5	4
1	2	2	1	1	2	3	9	4	5
2	2	2	1	1	1	4	8	4	5
3	3	2	1	1	1	4	9	3	3
3	2	2	1	1	3	2	9	3	2
2	2	2	1	1	1	4	9	0	3
3	3	1	1	1	2	3	5	1	4
2	1	2	1	1	1	4	8	3	6
1	2	2	1	1	2	3	8	5	4
2	2	2	1	1	1	4	7	5	5
1	3	2	1	1	1	4	9	4	6
2	2	2	1	1	1	4	6	3	5
2	2	2	1	1	2	3	8	2	4
2	2	1	1	1	2	3	9	2	3
2	2	2	1	1	1	4	7	3	2
3	2	1	1	1	1	4	9	3	2
2	2	2	1	1	1	4	7	3	2
2	2	2	1	1	2	3	5	2	3
2	2	2	2	2	2	3	9	4	6
2	2	2	1	1	2	3	9	1	2
2	2	2	1	1	2	3	9	2	3
1	2	2	1	1	1	4	8	2	4
2	2	2	1	1	2	3	9	3	6
2	2	2	1	1	1	4	7	2	6
3	2	2	1	1	1	4	9	4	4
2	2	2	1	1	2	3	8	1	2
2	3	2	1	1	1	4	8	2	5
2	2	2	1	1	2	3	7	3	2
2	2	2	2	2	4	1	6	2	2
2	2	2	3	2	2	3	7	1	4
3	2	1	1	1	1	4	5	2	1
2	2	2	1	1	3	2	9	4	4

1	3	2	2	2	2	3	9	5	5
2	2	2	2	2	1	4	6	3	6
2	1	2	1	1	1	4	8	3	4
2	2	2	2	2	3	2	9	1	4
1	1	1	1	1	3	2	6	1	2
1	1	2	1	1	2	3	8	5	4
2	2	2	1	1	2	3	5	1	2
2	2	2	1	1	2	3	6	4	2
2	2	2	1	1	1	4	9	2	3
2	2	2	1	1	1	4	9	5	4
1	2	2	2	2	3	2	8	1	5
2	2	2	2	2	1	4	7	3	4
2	2	2	1	1	1	4	9	4	4
1	2	2	2	2	1	4	8	1	3
2	2	2	2	2	1	4	7	3	5
3	2	2	3	2	1	4	7	3	5
2	2	2	2	2	3	2	5	2	1
1	2	2	2	2	1	4	9	2	5
2	2	2	2	2	1	4	9	3	3
1	2	2	1	1	1	4	9	1	5
2	2	2	1	1	2	3	8	3	4
2	2	2	2	2	2	3	7	4	5
1	2	2	2	2	2	3	7	2	5
2	2	1	2	2	1	4	9	3	1
2	2	2	1	1	1	4	7	5	3
2	1	2	2	2	2	3	9	2	3
3	2	2	1	1	2	3	4	5	5
3	2	2	1	1	1	4	5	4	3
2	2	2	1	1	3	2	9	2	3
3	3	1	1	1	2	3	6	1	1
1	2	2	1	1	2	3	8	5	6
1	2	2	1	1	2	3	5	3	0
2	2	2	2	2	2	3	6	2	5
2	2	2	1	1	2	3	7	3	4
2	2	2	1	1	1	4	7	5	3
2	2	2	1	1	2	3	5	5	4
1	2	2	2	2	2	3	5	2	2
1	2	2	1	1	2	3	8	1	6
2	3	1	1	1	2	3	5	2	3
2	1	2	3	2	1	4	6	2	3
2	2	2	1	1	2	3	7	3	4
2	2	2	1	1	2	3	6	4	4
2	2	2	1	1	2	3	5	4	4
3	2	2	1	1	3	2	5	2	5
2	2	2	1	1	2	3	9	0	1
2	2	2	2	2	3	2	9	2	3
2	2	2	1	1	2	3	5	4	6
2	2	2	1	1	1	4	9	3	2
2	2	2	1	1	2	3	8	4	4
2	2	2	1	1	2	3	5	1	1

2	2	2	1	1	1	4	7	3	1
2	2	2	2	2	2	3	7	2	1
1	2	1	1	1	1	4	5	3	4
2	2	2	3	2	3	2	6	2	0
2	2	2	2	2	2	3	6	3	1
3	2	2	1	1	3	2	9	3	2
2	2	2	2	2	2	3	9	3	3
2	2	2	1	1	1	4	8	3	4
2	2	2	3	2	1	4	7	2	4
2	2	2	2	2	1	4	7	1	1
1	2	2	1	1	3	2	5	4	5
3	2	2	1	1	1	4	5	3	4
2	2	2	2	2	2	3	5	1	4
2	2	2	1	1	3	2	5	4	4
2	2	2	1	1	1	4	8	2	2
2	2	2	1	1	3	2	6	4	3
2	2	1	1	1	2	3	9	4	4
2	2	2	1	1	3	2	5	0	2
2	2	2	2	2	2	3	8	2	3
2	2	2	3	2	1	4	7	3	6
2	2	2	1	1	1	4	9	3	4
2	2	2	1	1	1	4	9	4	6
2	2	2	2	2	1	4	9	3	3
2	2	2	1	1	2	3	4	3	2
2	3	1	1	1	1	4	8	3	3
2	2	2	1	1	2	3	6	4	3
3	2	1	1	1	2	3	9	3	2
2	2	2	1	1	3	2	5	2	3
2	2	2	1	1	1	4	9	4	5
2	2	1	1	1	4	1	9	3	4
2	2	2	1	1	1	4	7	3	5
2	2	2	1	1	2	3	8	5	3
1	2	1	1	1	4	1	9	4	5
2	2	2	1	1	1	4	7	4	5
2	2	2	1	1	2	3	7	4	6
2	2	1	1	1	1	4	9	3	5
1	1	2	1	1	1	4	5	3	3
2	2	2	1	1	1	4	9	3	5
2	2	2	1	1	1	4	7	3	6
2	2	2	1	1	4	1	7	4	3
1	2	2	1	1	1	4	9	4	4
2	2	2	2	2	2	3	8	4	4
2	2	2	1	1	1	4	9	3	2
3	3	2	2	2	2	3	5	1	3
2	2	2	1	1	1	4	4	1	3
2	2	2	1	1	1	4	6	2	2
2	2	2	1	1	2	3	9	3	6
2	3	1	1	1	2	3	8	2	4
3	2	2	2	2	2	3	5	4	1
3	2	2	1	1	1	4	7	1	4

3	2	2	3	2	1	4	9	3	3
1	2	2	1	1	2	3	8	3	5
2	2	2	3	2	2	3	5	5	1
1	3	2	2	2	1	4	8	1	3
2	2	2	2	2	1	4	7	4	1
2	2	2	1	1	1	4	9	2	2
2	2	2	2	2	2	3	5	3	6
3	2	2	2	2	1	4	7	3	3
1	2	2	3	2	2	3	7	2	4
1	2	2	1	1	1	4	9	3	5
2	2	2	1	1	2	3	5	5	3
3	3	2	1	1	1	4	6	4	2
2	2	2	2	2	2	3	6	3	2
2	2	2	2	2	2	3	7	3	1
2	2	1	1	1	1	4	9	2	2
3	2	2	2	2	1	4	8	3	2
2	2	2	1	1	1	4	9	4	4
1	2	2	2	2	4	1	5	1	3
2	2	2	1	1	1	4	9	3	4
2	2	2	1	1	1	4	6	3	6
2	2	2	2	2	1	4	9	4	1
2	2	2	1	1	1	4	5	0	4
2	2	2	1	1	1	4	5	3	1
2	1	2	2	2	1	4	8	3	2
3	2	2	1	1	1	4	9	1	3
2	2	2	2	2	2	3	9	1	3
2	2	2	2	2	1	4	8	1	3
2	2	2	2	2	2	3	9	1	0
2	2	2	1	1	1	4	9	2	3
2	2	2	2	2	2	3	6	3	5
2	2	2	1	1	1	4	7	3	2
3	2	2	1	1	1	4	5	4	1
2	2	1	1	1	1	4	9	1	5
2	2	2	1	1	3	2	5	3	5
1	2	2	3	2	1	4	5	2	3
2	2	1	3	2	4	1	9	1	2
2	2	2	2	2	2	3	7	4	0
3	2	2	1	1	1	4	6	2	2
2	2	2	2	2	2	3	6	2	1
1	3	2	2	2	2	3	7	3	0
2	2	2	2	2	1	4	7	2	2
2	2	2	2	2	2	3	8	1	4
2	2	2	2	2	1	4	7	2	1
2	2	2	1	1	2	3	7	3	3
2	2	2	2	2	2	3	7	2	3
2	2	2	1	1	1	4	8	3	1
2	1	2	2	2	2	3	5	3	2
2	2	2	2	2	1	4	2	4	2
2	2	2	1	1	2	3	8	3	3
2	2	2	2	2	1	4	5	5	3

2	2	2	1	1	2	3	7	5	6
3	2	2	1	1	3	2	7	3	2
2	2	2	3	2	2	3	5	2	3
2	2	2	1	1	1	4	8	4	5
2	2	2	2	2	1	4	9	2	3
1	2	2	2	2	1	4	6	2	2
2	1	2	1	1	1	4	7	4	5
1	2	2	1	1	1	4	8	4	5
2	2	2	1	1	2	3	5	4	3
1	2	2	2	2	1	4	6	2	3
2	2	2	1	1	2	3	5	3	2
2	2	2	1	1	2	3	5	4	4
3	2	2	1	1	2	3	7	4	3
2	2	1	1	1	1	4	6	1	4
2	2	2	2	2	1	4	7	5	2
2	2	2	1	1	1	4	6	2	3
2	2	2	1	1	2	3	8	2	3
2	2	2	2	2	2	3	6	4	3
1	2	2	1	1	2	3	8	3	5
1	2	2	1	1	2	3	7	3	5
3	2	2	2	2	1	4	8	2	2
2	2	2	1	1	2	3	6	3	0
2	2	2	2	2	1	4	8	1	3
2	2	2	1	1	2	3	9	4	3
3	2	2	1	1	2	3	5	1	3
2	2	2	1	1	2	3	5	3	3
2	2	2	1	1	2	3	6	4	4
2	2	2	2	2	2	3	5	3	1
1	2	1	1	1	1	4	7	4	4
3	2	1	1	1	3	2	7	3	3
3	2	2	1	1	3	2	7	2	2
1	2	2	1	1	2	3	9	3	5
2	2	2	1	1	2	3	8	4	3
2	2	2	2	2	2	3	9	2	2
2	2	2	1	1	1	4	7	5	3
3	2	2	1	1	2	3	8	4	5
2	2	2	2	2	2	3	5	3	5
3	3	2	1	1	3	2	5	1	0
1	2	1	1	1	2	3	5	3	3
2	2	2	1	1	2	3	5	1	4
2	2	2	1	1	2	3	9	3	2
2	3	2	1	1	2	3	6	2	1
2	2	2	3	2	2	3	7	4	3
1	2	2	1	1	1	4	7	5	5
3	2	2	1	1	2	3	7	4	5
2	2	2	2	2	2	3	7	2	6
2	2	2	2	2	1	4	8	2	4
1	2	2	2	2	1	4	9	2	3
2	2	2	1	1	1	4	6	5	6
2	2	2	2	2	1	4	5	2	2

2	2	2	2	2	2	3	7	2	4
2	2	2	2	2	1	4	7	1	2
2	2	2	1	1	2	3	9	2	5
2	1	2	2	2	1	4	9	4	2
2	2	2	3	2	1	4	8	3	2
2	2	2	2	2	2	3	7	5	4
2	2	2	1	1	2	3	8	2	2
2	2	2	1	1	1	4	8	3	5
2	2	2	1	1	2	3	6	4	3
2	2	2	2	2	1	4	9	2	3
1	3	2	1	1	2	3	7	3	2
2	2	1	1	1	2	3	5	3	3
2	2	2	1	1	1	4	7	3	3
2	2	1	1	1	1	4	7	1	4
2	2	2	2	2	2	3	5	3	0
2	2	2	1	1	2	3	8	5	3
2	2	2	1	1	2	3	5	2	2
2	2	2	1	1	2	3	5	3	2
1	3	2	1	1	2	3	9	4	3
2	2	2	1	1	1	4	7	4	4
1	2	2	1	1	2	3	9	1	4
2	2	2	1	1	2	3	8	2	2
2	2	2	3	2	2	3	5	4	2
2	2	2	1	1	2	3	6	3	3
2	2	2	1	1	2	3	3	1	3
2	2	2	1	1	1	4	8	3	3
2	2	2	1	1	1	4	8	3	3
2	2	2	3	2	2	3	7	3	2
2	2	2	1	1	1	4	7	3	3
3	2	2	1	1	2	3	6	2	3
2	2	2	1	1	1	4	7	2	4
2	2	2	2	2	1	4	7	1	0
2	2	2	2	2	1	4	7	3	3
2	2	2	2	2	3	2	7	2	2
2	2	2	2	2	1	4	9	3	3
2	2	1	2	2	2	3	4	3	4
2	2	2	2	2	1	4	7	1	2
3	2	2	1	1	2	3	7	4	4
2	2	2	1	1	2	3	8	2	3
2	2	2	1	1	1	4	7	2	3
2	2	2	1	1	2	3	5	2	5
2	2	2	1	1	2	3	9	2	0
2	2	2	1	1	1	4	7	0	4
3	2	2	1	1	2	3	8	2	3
2	2	2	1	1	2	3	7	3	3
2	2	2	2	2	1	4	6	2	3
2	1	2	2	2	1	4	9	2	1
3	2	2	1	1	1	4	8	2	2
2	2	2	2	2	2	3	9	4	1
2	2	2	1	1	1	4	7	3	4

2	2	2	3	2	1	4	1	4	5
2	2	2	2	2	3	2	5	3	4
2	2	2	1	1	1	4	8	2	4
2	2	2	1	1	1	4	9	3	3
2	2	2	1	1	1	4	9	2	2
3	2	2	1	1	1	4	9	3	4
2	2	2	2	2	1	4	7	3	2
2	2	2	2	2	1	4	7	3	2
2	2	2	1	1	1	4	6	3	3
1	2	2	1	1	2	3	6	2	3
2	2	2	1	1	2	3	5	2	3
2	2	2	1	1	2	3	9	4	4
2	2	2	3	2	2	3	9	2	3
2	2	2	2	2	2	3	5	3	3
1	2	2	1	1	3	2	5	2	4
3	3	2	1	1	3	2	5	3	3
3	2	2	3	2	2	3	6	3	3
2	2	2	2	2	2	3	9	2	6
2	2	2	1	1	2	3	5	2	4
2	2	2	3	2	1	4	9	2	3
2	2	2	2	2	2	3	9	3	2
2	2	2	2	2	2	3	9	3	4
2	2	2	1	1	2	3	5	4	3
2	2	2	2	2	2	3	5	3	4
2	2	2	1	1	2	3	8	2	4
2	2	2	3	2	2	3	7	4	5
2	2	2	2	2	1	4	7	5	3
1	2	2	1	1	2	3	7	2	4
2	2	2	1	1	1	4	9	2	5
2	2	2	2	2	2	3	6	3	4
2	2	2	1	1	2	3	9	3	5
2	2	2	1	1	1	4	9	4	3
2	2	2	1	1	2	3	7	2	4
2	2	2	1	1	1	4	1	2	2
2	2	2	1	1	2	3	7	2	4
2	2	2	1	1	1	4	9	5	5
1	2	1	1	1	2	3	7	4	4
1	3	2	1	1	3	2	6	3	4
2	2	2	1	1	1	4	4	4	4

Epid	Med	Sym	Knowl	Knowlper	Suscep	Sev	Ben	Bar	TotalHB
2	1	2	11	47.82609	4	6	24	12	46
2	2	1	11	47.82609	7	10	24	18	59
0	4	1	13	56.52174	8	11	24	15	58
1	1	2	11	47.82609	10	12	17	16	55
2	1	2	12	52.17391	7	11	23	15	56
1	0	2	8	34.78261	12	12	18	18	60
2	2	3	15	65.21739	9	11	18	14	52
2	1	3	13	56.52174	4	9	24	9	46
0	0	0	3	13.04348	8	8	12	18	46
0	2	2	8	34.78261	6	10	18	11	45
2	2	2	15	65.21739	4	8	23	12	47
3	2	2	12	52.17391	10	9	18	13	50
2	3	1	12	52.17391	7	9	13	12	41
1	3	0	9	39.13043	8	10	23	11	52
1	1	1	10	43.47826	7	12	15	17	51
1	2	1	8	34.78261	8	10	20	15	53
1	1	2	14	60.86957	4	9	24	14	51
1	2	1	10	43.47826	4	9	24	14	51
2	2	1	14	60.86957	9	10	18	17	54
1	1	1	5	21.73913	8	11	19	16	54
0	1	2	10	43.47826	8	10	24	14	56
0	2	1	7	30.43478	12	8	21	15	56
2	3	1	13	56.52174	7	11	20	14	52
3	1	1	13	56.52174	7	10	24	17	58
0	3	3	13	56.52174	12	11	18	16	57
2	2	1	15	65.21739	5	10	23	10	48
0	2	1	9	39.13043	10	10	22	14	56
2	2	1	12	52.17391	8	11	17	14	50
2	2	2	12	52.17391	10	9	20	16	55
2	2	1	8	34.78261	7	10	19	15	51
3	2	0	10	43.47826	7	9	23	14	53
1	3	1	12	52.17391	7	4	24	14	49
1	1	1	11	47.82609	4	8	23	23	58
2	3	1	17	73.91304	6	7	21	15	49
3	4	0	14	60.86957	7	8	22	13	50
2	3	2	15	65.21739	8	7	24	9	48
2	1	2	12	52.17391	9	12	23	9	53
2	2	1	13	56.52174	11	12	24	12	59
1	3	0	12	52.17391	10	15	24	14	63
0	1	1	11	47.82609	6	10	23	13	52
2	4	2	19	82.6087	10	11	19	16	56
3	4	0	15	65.21739	6	12	20	16	54
3	3	1	18	78.26087	10	13	24	16	63
3	3	2	16	69.56522	5	11	21	15	52
1	3	2	10	43.47826	7	12	22	19	60
2	3	3	15	65.21739	10	9	24	16	59
0	2	1	14	60.86957	4	12	24	16	56
0	1	2	7	30.43478	4	9	23	18	54
3	2	1	16	69.56522	4	9	24	17	54

0	2	1	11	47.82609	8	11	24	15	58
1	1	2	10	43.47826	8	7	22	13	50
1	1	2	11	47.82609	4	12	24	19	59
1	2	2	11	47.82609	4	13	18	12	47
3	3	3	17	73.91304	11	9	18	16	54
2	4	1	17	73.91304	5	10	20	14	49
1	0	1	9	39.13043	8	12	23	12	55
2	2	2	15	65.21739	8	12	24	13	57
3	3	1	14	60.86957	10	12	23	11	56
1	3	2	12	52.17391	7	10	23	12	52
3	2	1	14	60.86957	10	10	20	11	51
1	4	2	18	78.26087	8	12	18	14	52
2	2	1	10	43.47826	6	11	24	11	52
0	1	0	6	26.08696	4	11	24	13	52
1	2	1	10	43.47826	9	8	22	13	52
2	1	2	9	39.13043	8	9	18	15	50
2	0	1	8	34.78261	8	8	21	12	49
1	3	2	13	56.52174	4	5	20	12	41
1	3	1	10	43.47826	8	9	19	16	52
1	1	1	9	39.13043	6	11	24	19	60
2	2	3	12	52.17391	9	8	18	16	51
1	1	0	8	34.78261	7	10	21	12	50
1	1	0	12	52.17391	11	10	24	17	62
1	3	2	12	52.17391	10	9	21	18	58
2	3	2	14	60.86957	9	11	19	14	53
1	3	1	9	39.13043	6	4	24	21	55
1	2	1	12	52.17391	5	10	20	10	45
2	1	2	11	47.82609	10	10	21	15	56
2	4	2	14	60.86957	8	16	24	14	62
1	3	2	13	56.52174	8	9	23	14	54
2	4	2	15	65.21739	4	6	20	15	45
2	2	2	15	65.21739	5	7	24	9	45
3	4	3	18	78.26087	4	14	24	9	51
3	3	2	16	69.56522	10	10	18	12	50
1	2	2	13	56.52174	9	5	23	19	56
1	0	0	7	30.43478	10	12	23	15	60
1	2	1	9	39.13043	9	9	23	13	54
2	3	1	12	52.17391	9	11	21	13	54
2	3	2	14	60.86957	11	14	23	15	63
1	2	2	12	52.17391	8	14	24	15	61
2	3	2	15	65.21739	5	9	21	14	49
1	3	2	15	65.21739	11	11	21	13	56
1	2	2	13	56.52174	5	9	24	12	50
3	3	2	14	60.86957	11	11	19	16	57
2	1	2	10	43.47826	8	13	22	14	57
3	2	2	13	56.52174	8	9	24	15	56
2	3	2	12	52.17391	7	8	24	16	55
2	2	3	10	43.47826	7	12	18	12	49
2	1	0	8	34.78261	7	8	24	13	52
1	1	3	8	34.78261	4	11	24	13	52

1	2	1	10	43.47826	4	11	24	15	54
1	2	2	9	39.13043	12	11	22	20	65
2	3	3	14	60.86957	6	11	24	16	57
2	3	1	14	60.86957	4	14	24	15	57
1	1	3	11	47.82609	5	10	24	11	50
2	1	2	14	60.86957	9	13	24	18	64
1	2	2	7	30.43478	4	10	24	14	52
2	0	3	8	34.78261	8	10	21	12	51
2	2	1	13	56.52174	4	9	24	12	49
3	2	2	17	73.91304	5	12	6	14	37
2	3	1	15	65.21739	7	12	23	13	55
3	4	3	17	73.91304	7	9	24	12	52
2	2	2	12	52.17391	6	8	19	18	51
3	4	1	14	60.86957	12	11	12	16	51
1	1	0	8	34.78261	7	6	23	13	49
2	1	2	11	47.82609	4	13	23	12	52
2	3	2	16	69.56522	7	11	24	10	52
1	2	0	7	30.43478	7	11	24	16	58
1	1	2	6	26.08696	10	8	19	17	54
1	2	0	10	43.47826	5	7	20	13	45
3	2	2	13	56.52174	6	10	20	8	44
1	4	2	12	52.17391	7	5	21	15	48
2	4	0	9	39.13043	9	11	24	16	60
2	6	1	16	69.56522	8	10	24	17	59
0	1	1	8	34.78261	8	9	22	17	56
2	1	2	9	39.13043	7	10	8	13	38
0	0	2	4	17.3913	9	8	24	13	54
3	2	0	12	52.17391	10	10	23	9	52
3	4	2	19	82.6087	4	10	22	11	47
1	1	1	11	47.82609	6	8	24	14	52
0	4	2	13	56.52174	11	11	23	13	58
1	0	0	4	17.3913	8	11	18	14	51
1	1	1	4	17.3913	10	9	13	12	44
2	3	2	14	60.86957	8	10	18	13	49
3	3	2	18	78.26087	9	6	24	15	54
1	2	1	14	60.86957	8	9	18	13	48
2	4	3	18	78.26087	12	10	24	13	59
2	4	2	16	69.56522	9	7	24	12	52
3	3	0	14	60.86957	4	10	18	12	44
3	4	2	17	73.91304	9	8	18	13	48
1	4	1	13	56.52174	8	11	21	14	54
2	4	1	12	52.17391	8	13	24	14	59
1	5	2	14	60.86957	12	11	24	16	63
2	3	0	13	56.52174	10	12	24	10	56
2	3	1	14	60.86957	11	12	19	18	60
2	3	3	15	65.21739	7	10	17	15	49
2	5	3	18	78.26087	6	10	22	14	52
3	4	1	16	69.56522	4	8	23	13	48
1	1	1	6	26.08696	8	8	18	13	47
1	1	2	7	30.43478	12	12	18	15	57

1	0	0	6	26.08696	9	8	14	13	44
3	3	2	14	60.86957	6	8	19	17	50
2	4	2	16	69.56522	10	7	18	15	50
2	4	2	16	69.56522	10	7	21	13	51
2	4	2	16	69.56522	10	7	21	14	52
1	4	0	11	47.82609	4	4	24	12	44
2	3	2	16	69.56522	9	11	23	11	54
2	2	0	13	56.52174	4	6	24	8	42
3	3	3	18	78.26087	10	12	24	18	64
3	3	3	17	73.91304	4	10	24	10	48
1	2	2	9	39.13043	10	9	23	12	54
2	5	0	16	69.56522	8	10	18	14	50
2	4	3	18	78.26087	8	10	23	14	55
2	5	1	15	65.21739	5	8	23	17	53
3	5	1	19	82.6087	5	9	18	12	44
2	2	3	13	56.52174	9	8	17	14	48
2	4	3	17	73.91304	9	12	24	15	60
3	4	3	19	82.6087	8	10	23	18	59
2	5	2	16	69.56522	9	4	20	15	48
2	3	3	14	60.86957	9	8	18	14	49
2	2	1	11	47.82609	8	9	16	15	48
1	3	2	13	56.52174	11	7	23	11	52
2	5	2	20	86.95652	8	9	18	12	47
2	4	1	13	56.52174	4	5	12	10	31
2	3	2	16	69.56522	8	7	21	16	52
2	2	3	14	60.86957	7	11	18	10	46
0	1	0	3	13.04348	5	4	18	14	41
1	2	2	12	52.17391	8	9	24	13	54
2	4	1	14	60.86957	11	4	24	14	53
3	6	1	18	78.26087	6	12	18	12	48
3	2	1	14	60.86957	6	4	24	11	45
2	0	3	11	47.82609	4	14	24	17	59
2	6	3	22	95.65217	8	16	24	20	68
2	4	3	15	65.21739	8	10	24	14	56
2	2	2	14	60.86957	8	6	24	15	53
2	5	1	15	65.21739	10	9	18	19	56
3	4	3	19	82.6087	4	8	18	16	46
2	4	1	15	65.21739	6	6	24	8	44
0	3	2	13	56.52174	6	9	24	14	53
3	3	2	16	69.56522	12	9	21	15	57
3	5	3	19	82.6087	6	7	24	8	45
2	3	2	13	56.52174	10	11	24	11	56
3	3	2	16	69.56522	9	9	18	15	51
0	1	1	4	17.3913	13	13	23	15	64
2	2	2	13	56.52174	5	11	24	16	56
2	4	2	15	65.21739	4	10	24	15	53
3	4	3	17	73.91304	11	11	24	15	61
1	2	3	15	65.21739	4	12	24	14	54
1	1	0	3	13.04348	12	10	18	13	53
1	3	1	10	43.47826	6	9	18	16	49

1	1	0	3	13.04348	10	11	20	16	57
1	4	3	16	69.56522	8	12	22	17	59
1	4	2	16	69.56522	6	8	24	12	50
3	4	2	16	69.56522	11	5	21	14	51
1	5	1	15	65.21739	8	8	22	15	53
1	3	2	12	52.17391	9	11	24	16	60
1	2	3	14	60.86957	4	9	18	13	44
1	3	3	14	60.86957	8	11	20	17	56
1	2	1	11	47.82609	11	9	17	14	51
3	4	1	16	69.56522	9	9	18	15	51
2	4	3	18	78.26087	8	14	24	19	65
2	3	2	16	69.56522	4	10	24	12	50
2	4	3	17	73.91304	9	11	18	12	50
1	2	1	10	43.47826	10	14	23	14	61
2	4	3	17	73.91304	6	11	24	17	58
2	3	2	16	69.56522	6	12	23	21	62
2	4	2	18	78.26087	12	11	23	17	63
3	6	2	19	82.6087	7	11	19	14	51
1	2	2	12	52.17391	7	15	16	15	53
2	4	1	13	56.52174	11	11	18	14	54
3	0	2	8	34.78261	8	7	12	14	41
2	3	0	13	56.52174	10	9	23	14	56
3	4	1	14	60.86957	11	10	21	19	61
3	4	1	17	73.91304	5	10	24	15	54
2	5	1	15	65.21739	8	11	23	13	55
1	5	2	14	60.86957	9	11	16	13	49
1	3	0	9	39.13043	4	10	23	14	51
2	5	2	17	73.91304	7	10	17	15	49
2	4	3	19	82.6087	11	8	18	12	49
1	1	1	9	39.13043	12	9	17	18	56
2	3	2	13	56.52174	10	12	12	23	57
2	1	2	13	56.52174	5	14	23	17	59
1	4	2	16	69.56522	10	8	17	16	51
3	2	1	12	52.17391	9	12	22	17	60
2	4	3	17	73.91304	8	11	18	11	48
3	3	3	17	73.91304	9	11	18	15	53
3	4	3	19	82.6087	16	16	18	18	68
2	5	2	18	78.26087	8	9	24	15	56
3	2	2	16	69.56522	6	12	18	16	52
2	3	1	12	52.17391	8	11	18	15	52
2	2	2	14	60.86957	8	11	24	17	60
3	2	2	13	56.52174	8	9	21	13	51
2	2	1	12	52.17391	10	5	17	20	52
3	5	3	17	73.91304	7	11	21	14	53
3	3	3	16	69.56522	4	11	23	15	53
2	4	1	16	69.56522	7	5	24	9	45
2	2	1	11	47.82609	7	6	6	8	27
2	5	2	15	65.21739	6	10	13	12	41
1	2	1	6	26.08696	8	8	13	10	39
1	2	1	12	52.17391	4	14	24	9	51

2	3	2	14	60.86957	8	8	23	13	52
2	5	2	17	73.91304	8	8	23	13	52
2	5	2	18	78.26087	6	8	18	15	47
2	2	1	12	52.17391	8	9	20	15	52
1	1	2	9	39.13043	9	8	21	16	54
0	4	2	12	52.17391	4	12	22	16	54
2	3	1	11	47.82609	5	6	14	16	41
2	1	2	13	56.52174	8	11	20	13	52
3	4	1	15	65.21739	8	9	18	18	53
3	3	2	14	60.86957	11	16	23	23	73
1	3	1	7	30.43478	8	10	18	13	49
3	0	1	9	39.13043	6	11	12	22	51
3	3	1	11	47.82609	5	6	14	8	33
1	3	2	11	47.82609	8	11	23	16	58
3	4	2	17	73.91304	5	11	23	15	54
2	4	1	14	60.86957	9	8	23	16	56
3	3	0	13	56.52174	8	10	20	15	53
2	3	1	14	60.86957	8	12	24	13	57
1	2	3	13	56.52174	9	12	18	15	54
3	3	2	15	65.21739	6	11	23	14	54
3	4	3	19	82.6087	6	8	21	17	52
3	3	1	15	65.21739	8	13	18	21	60
1	3	2	11	47.82609	11	8	22	14	55
3	4	1	17	73.91304	4	10	23	14	51
2	5	3	18	78.26087	8	11	18	15	52
2	5	3	18	78.26087	7	11	24	18	60
1	2	3	13	56.52174	8	8	15	16	47
2	3	1	14	60.86957	8	8	15	17	48
2	2	3	12	52.17391	5	11	24	15	55
2	3	1	13	56.52174	8	11	24	16	59
3	5	2	17	73.91304	7	8	23	13	51
2	4	3	14	60.86957	7	8	23	13	51
2	3	1	11	47.82609	11	15	23	16	65
1	3	1	11	47.82609	11	15	23	17	66
2	4	1	15	65.21739	4	13	24	18	59
2	3	0	9	39.13043	6	12	19	20	57
2	3	0	10	43.47826	8	13	24	18	63
3	2	0	14	60.86957	8	9	22	15	54
2	5	3	15	65.21739	10	11	18	18	57
2	0	1	10	43.47826	11	11	18	16	56
3	5	0	15	65.21739	6	11	21	12	50
2	3	3	15	65.21739	7	7	24	16	54
2	4	1	15	65.21739	9	12	6	17	44
1	1	0	8	34.78261	8	11	24	14	57
2	3	3	16	69.56522	8	16	24	12	60
3	4	3	17	73.91304	8	10	21	14	53
2	5	0	16	69.56522	9	12	23	12	56
2	2	2	14	60.86957	10	13	24	20	67
2	4	3	17	73.91304	9	12	18	17	56
2	2	1	14	60.86957	12	10	24	15	61

2	2	3	17	73.91304	10	10	20	9	49
2	3	1	12	52.17391	8	11	20	14	53
3	1	1	12	52.17391	4	9	24	16	53
2	5	2	18	78.26087	8	7	17	16	48
2	1	1	10	43.47826	8	11	24	16	59
3	2	0	13	56.52174	8	9	23	15	55
2	3	2	14	60.86957	5	4	18	6	33
2	3	3	15	65.21739	11	8	24	18	61
3	4	2	17	73.91304	11	9	23	10	53
2	4	2	16	69.56522	4	9	24	15	52
1	1	1	10	43.47826	9	9	20	15	53
0	3	2	13	56.52174	7	11	18	16	52
2	2	1	15	65.21739	6	10	23	13	52
3	3	2	16	69.56522	9	9	24	16	58
2	3	2	15	65.21739	8	8	17	16	49
2	4	2	17	73.91304	8	11	20	15	54
2	3	2	14	60.86957	11	13	18	14	56
3	3	3	18	78.26087	10	9	19	11	49
3	3	0	12	52.17391	6	11	23	17	57
1	2	2	10	43.47826	8	14	24	19	65
3	2	2	12	52.17391	8	9	17	14	48
3	3	1	16	69.56522	10	11	24	17	62
0	0	2	10	43.47826	8	9	24	11	52
2	4	2	14	60.86957	8	9	20	19	56
2	3	3	14	60.86957	8	9	21	12	50
3	3	3	18	78.26087	8	12	18	13	51
2	6	1	19	82.6087	8	11	24	15	58
3	2	2	17	73.91304	6	11	18	16	51
3	2	3	16	69.56522	10	14	24	16	64
3	2	2	16	69.56522	10	10	18	15	53
1	2	0	10	43.47826	8	11	22	16	57
2	5	2	17	73.91304	8	12	21	13	54
1	2	3	14	60.86957	4	11	24	16	55
1	2	1	10	43.47826	9	8	18	17	52
3	6	1	18	78.26087	10	8	22	15	55
1	5	2	16	69.56522	9	10	23	11	53
2	3	3	16	69.56522	5	11	20	13	49
1	4	3	18	78.26087	7	14	24	21	66
3	3	3	16	69.56522	10	12	18	17	57
3	3	2	17	73.91304	8	7	24	14	53
3	4	3	19	82.6087	8	13	21	19	61
3	2	2	17	73.91304	6	8	17	13	44
3	4	0	16	69.56522	9	9	18	15	51
2	3	0	12	52.17391	9	9	18	15	51
1	3	2	15	65.21739	7	10	24	17	58
3	4	0	15	65.21739	8	8	24	16	56
2	1	3	14	60.86957	8	12	18	13	51
1	6	1	19	82.6087	7	11	24	13	55
1	5	1	13	56.52174	8	11	20	15	54
2	2	3	13	56.52174	9	8	23	12	52

2	2	0	11	47.82609	9	11	18	17	55
3	2	3	17	73.91304	12	11	24	16	63
1	5	3	19	82.6087	9	7	20	11	47
2	6	2	21	91.30435	8	10	17	15	50
3	3	3	16	69.56522	8	13	18	19	58
2	5	2	17	73.91304	8	10	24	10	52
2	4	0	14	60.86957	8	10	18	12	48
3	5	3	21	91.30435	11	12	17	14	54
3	6	3	21	91.30435	5	11	23	15	54
1	5	1	15	65.21739	12	8	22	15	57
3	5	2	18	78.26087	4	13	24	9	50
0	5	3	16	69.56522	5	11	24	14	54
3	3	2	13	56.52174	8	7	19	12	46
3	3	3	15	65.21739	11	16	6	16	49
1	5	3	18	78.26087	6	15	22	15	58
1	3	2	13	56.52174	8	8	18	15	49
3	5	1	18	78.26087	8	11	24	15	58
3	3	3	16	69.56522	8	11	18	14	51
3	4	3	19	82.6087	10	16	20	17	63
3	3	2	16	69.56522	4	4	24	14	46
2	3	3	17	73.91304	5	12	24	8	49
2	2	2	12	52.17391	8	14	24	16	62
1	2	2	12	52.17391	8	10	24	17	59
3	3	1	12	52.17391	8	11	24	9	52
3	4	1	13	56.52174	8	12	18	16	54
2	2	2	14	60.86957	7	8	24	15	54
3	5	1	15	65.21739	5	9	19	12	45
2	2	3	13	56.52174	9	12	23	11	55
2	4	0	10	43.47826	6	8	15	15	44
2	6	2	17	73.91304	10	8	18	18	54
3	4	1	18	78.26087	10	11	21	12	54
3	5	2	20	86.95652	7	7	24	11	49
2	1	2	11	47.82609	8	10	13	18	49
1	3	2	14	60.86957	7	15	20	19	61
2	3	1	12	52.17391	8	11	18	12	49
1	2	3	14	60.86957	6	11	21	13	51
2	3	3	17	73.91304	6	11	21	15	53
1	3	2	16	69.56522	8	11	18	17	54
3	3	3	17	73.91304	11	11	18	16	56
2	4	3	20	86.95652	8	12	18	14	52
2	4	2	14	60.86957	7	8	22	15	52
1	5	1	15	65.21739	6	15	22	16	59
1	5	1	15	65.21739	6	15	22	16	59
2	4	2	18	78.26087	8	12	18	12	50
2	4	2	17	73.91304	5	8	24	14	51
2	2	2	13	56.52174	8	10	24	18	60
1	5	1	15	65.21739	11	12	24	16	63
3	6	2	21	91.30435	4	8	23	12	47
3	5	1	19	82.6087	7	8	22	11	48
3	3	2	17	73.91304	7	13	23	17	60

2	3	1	11	47.82609	9	13	24	19	65
2	2	1	13	56.52174	7	8	18	18	51
1	2	2	13	56.52174	11	10	18	17	56
2	2	1	11	47.82609	7	12	21	20	60
1	1	0	6	26.08696	8	8	15	17	48
2	3	2	12	52.17391	10	10	16	13	49
1	2	2	11	47.82609	9	9	15	17	50
2	3	1	11	47.82609	9	12	18	14	53
1	3	2	11	47.82609	8	8	23	15	54
2	4	2	13	56.52174	9	9	18	15	51
2	1	1	14	60.86957	4	10	23	15	52
1	3	0	13	56.52174	8	11	22	19	60
1	2	2	12	52.17391	4	13	23	11	51
2	2	3	15	65.21739	8	11	24	15	58
2	4	1	15	65.21739	10	8	24	15	57
2	1	0	9	39.13043	8	10	18	11	47
3	3	1	13	56.52174	8	9	24	7	48
1	1	0	3	13.04348	7	4	12	12	35
1	2	1	8	34.78261	4	10	19	19	52
1	1	1	10	43.47826	4	14	19	19	56
2	1	2	9	39.13043	4	13	23	11	51
0	3	1	12	52.17391	9	11	21	10	51
1	3	2	15	65.21739	9	9	16	17	51
3	2	3	15	65.21739	4	10	24	18	56
1	2	2	10	43.47826	13	14	22	19	68
1	1	1	9	39.13043	11	9	24	17	61
2	3	1	15	65.21739	8	9	22	11	50
2	3	2	18	78.26087	8	13	23	19	63
2	1	2	14	60.86957	8	6	24	15	53
2	2	3	15	65.21739	5	11	23	6	45
3	4	1	17	73.91304	8	9	17	13	47
3	2	1	16	69.56522	7	11	23	10	51
1	2	1	11	47.82609	6	4	17	18	45
1	3	1	8	34.78261	8	8	19	14	49
2	2	3	14	60.86957	9	12	16	16	53
1	1	2	8	34.78261	11	15	23	16	65
2	2	3	16	69.56522	10	8	10	12	40
0	2	1	9	39.13043	4	10	18	16	48
2	1	1	8	34.78261	13	12	19	23	67
1	1	2	9	39.13043	10	9	18	15	52
3	2	3	16	69.56522	5	4	15	24	48
1	2	0	10	43.47826	10	8	17	19	54
1	3	2	14	60.86957	7	12	21	15	55
1	0	1	9	39.13043	10	10	18	17	55
1	5	2	17	73.91304	10	8	24	16	58
2	4	3	16	69.56522	7	6	22	14	49
2	3	1	9	39.13043	13	11	16	19	59
1	1	2	10	43.47826	8	11	18	16	53
1	1	0	7	30.43478	10	12	23	13	58
1	3	2	12	52.17391	8	8	17	12	45

2	1	2	12	52.17391	8	11	16	15	50
2	4	0	12	52.17391	8	13	18	15	54
2	2	1	16	69.56522	8	5	24	10	47
2	4	3	19	82.6087	10	10	24	16	60
2	4	1	15	65.21739	7	12	16	16	51
2	1	1	13	56.52174	10	8	18	14	50
2	0	3	10	43.47826	10	12	18	17	57
2	4	3	18	78.26087	12	12	21	14	59
1	2	1	12	52.17391	12	13	18	14	57
1	2	1	11	47.82609	13	14	21	19	67
1	1	1	10	43.47826	10	7	22	15	54
1	4	1	15	65.21739	7	13	18	17	55
1	3	3	17	73.91304	6	11	23	14	54
1	3	2	13	56.52174	9	9	21	13	52
2	2	2	14	60.86957	6	10	24	17	57
1	2	2	13	56.52174	8	11	15	14	48
2	5	2	17	73.91304	10	11	23	18	62
3	3	3	17	73.91304	10	15	24	18	67
2	4	3	18	78.26087	10	14	24	15	63
2	4	0	15	65.21739	9	11	18	16	54
1	2	0	12	52.17391	4	12	24	6	46
1	3	2	12	52.17391	7	15	24	10	56
2	2	1	10	43.47826	5	6	10	10	31
0	0	0	3	13.04348	4	11	24	13	52
1	2	2	10	43.47826	10	10	16	15	51
1	2	3	15	65.21739	8	11	24	16	59
2	3	3	17	73.91304	12	11	24	16	63
2	2	2	16	69.56522	10	11	18	12	51
2	0	3	15	65.21739	8	10	21	14	53
2	2	3	15	65.21739	6	9	20	15	50
1	3	2	12	52.17391	12	10	17	14	53
0	0	1	6	26.08696	8	10	24	17	59
0	2	1	8	34.78261	12	10	18	18	58
2	2	2	11	47.82609	8	12	14	18	52
2	2	3	12	52.17391	5	15	24	15	59
2	2	2	11	47.82609	13	16	23	20	72
2	3	2	17	73.91304	8	11	18	15	52
0	2	1	6	26.08696	8	8	18	16	50
2	0	3	10	43.47826	11	13	20	21	65
2	2	1	11	47.82609	12	9	15	15	51
3	2	2	16	69.56522	9	13	24	16	62
3	1	2	14	60.86957	8	10	18	15	51
0	0	1	9	39.13043	10	8	23	12	53
2	1	2	8	34.78261	10	11	22	13	56
2	2	1	12	52.17391	8	11	24	7	50
1	1	2	9	39.13043	9	11	21	13	54
2	4	0	10	43.47826	4	12	18	12	46
2	3	0	10	43.47826	12	9	11	15	47
2	2	1	8	34.78261	8	10	11	12	41
2	2	1	13	56.52174	10	10	18	14	52

3	3	2	18	78.26087	12	10	21	13	56
1	3	1	14	60.86957	8	9	20	15	52
3	2	2	14	60.86957	8	12	21	8	49
1	3	2	11	47.82609	10	10	14	13	47
2	3	1	9	39.13043	12	8	15	15	50
1	1	1	12	52.17391	10	10	22	15	57
2	0	1	6	26.08696	8	10	18	18	54
1	2	0	9	39.13043	9	9	18	16	52
1	2	2	10	43.47826	8	9	24	13	54
1	6	3	19	82.6087	8	10	18	11	47
1	2	1	10	43.47826	10	10	16	15	51
2	0	0	9	39.13043	8	12	15	15	50
1	2	2	13	56.52174	8	14	24	15	61
2	3	1	10	43.47826	10	14	18	16	58
1	4	1	14	60.86957	8	11	23	10	52
1	2	0	11	47.82609	8	9	15	16	48
1	1	1	6	26.08696	4	7	18	17	46
1	4	1	13	56.52174	8	8	19	14	49
2	2	2	12	52.17391	4	10	24	14	52
0	2	1	9	39.13043	6	10	24	9	49
1	2	2	12	52.17391	4	6	21	15	46
2	4	3	18	78.26087	9	14	23	14	60
2	2	1	12	52.17391	8	11	18	15	52
2	2	1	9	39.13043	10	8	14	15	47
3	0	0	11	47.82609	11	10	19	15	55
0	3	2	10	43.47826	6	8	18	14	46
1	4	0	15	65.21739	8	11	18	16	53
1	4	2	14	60.86957	8	12	18	13	51
1	1	2	9	39.13043	4	13	21	12	50
1	1	1	5	21.73913	10	11	13	14	48
2	1	1	15	65.21739	10	11	19	14	54
2	2	1	8	34.78261	9	8	18	12	47
2	3	3	15	65.21739	10	12	24	15	61
3	3	0	13	56.52174	12	13	18	16	59
2	3	0	13	56.52174	8	12	24	15	59
2	2	1	14	60.86957	8	9	18	16	51
1	1	0	6	26.08696	11	10	20	16	57
3	2	1	13	56.52174	8	11	19	14	52
1	3	2	11	47.82609	8	7	9	13	37
1	1	2	9	39.13043	8	7	16	15	46
2	3	3	15	65.21739	10	11	22	16	59
1	2	1	12	52.17391	8	8	14	16	46
1	4	1	14	60.86957	4	12	20	17	53
3	2	3	15	65.21739	6	12	19	16	53
0	2	2	5	21.73913	8	9	15	20	52
0	2	3	10	43.47826	5	11	17	13	46
2	3	2	17	73.91304	8	9	18	15	50
3	4	2	14	60.86957	8	14	23	13	58
1	4	1	14	60.86957	4	9	24	21	58
1	4	1	8	34.78261	8	10	12	14	44

1	1	1	7	30.43478	10	14	16	17	57
2	0	2	7	30.43478	10	8	20	16	54
1	2	0	10	43.47826	12	11	24	21	68
1	0	1	4	17.3913	8	9	21	19	57
2	2	0	8	34.78261	10	11	14	17	52
1	0	1	7	30.43478	8	13	18	17	56
1	2	3	12	52.17391	9	11	19	16	55
2	3	1	13	56.52174	10	12	18	13	53
1	1	2	10	43.47826	11	11	21	13	56
1	2	3	8	34.78261	6	4	16	10	36
0	2	1	12	52.17391	8	8	24	24	64
3	3	3	16	69.56522	6	12	24	14	56
1	1	0	7	30.43478	8	12	23	17	60
1	3	2	14	60.86957	9	8	20	14	51
2	2	1	9	39.13043	7	12	24	16	59
1	2	2	12	52.17391	11	9	19	24	63
0	2	2	12	52.17391	8	15	22	9	54
1	3	0	6	26.08696	8	11	18	17	54
1	3	1	10	43.47826	8	8	18	12	46
1	2	1	13	56.52174	10	13	24	15	62
1	1	1	10	43.47826	7	11	18	11	47
2	5	3	20	86.95652	8	9	24	15	56
2	0	2	10	43.47826	4	4	24	10	42
1	1	0	7	30.43478	6	10	22	12	50
2	1	0	9	39.13043	8	9	17	13	47
3	1	1	12	52.17391	9	11	9	14	43
1	2	1	9	39.13043	8	12	12	14	46
2	1	3	11	47.82609	11	9	17	18	55
0	4	1	14	60.86957	7	9	22	11	49
2	1	2	12	52.17391	12	16	24	24	76
1	2	0	11	47.82609	4	11	21	20	56
1	2	3	14	60.86957	4	9	18	16	47
2	2	3	16	69.56522	8	14	18	21	61
1	3	2	15	65.21739	11	11	22	13	57
3	3	1	17	73.91304	12	11	20	10	53
0	4	1	13	56.52174	8	10	19	13	50
1	2	1	10	43.47826	6	13	21	21	61
3	6	2	19	82.6087	8	9	18	13	48
2	4	2	17	73.91304	9	12	22	18	61
1	2	2	12	52.17391	9	9	16	13	47
2	3	2	15	65.21739	4	10	24	12	50
3	3	0	14	60.86957	9	7	18	17	51
1	2	1	9	39.13043	8	8	17	16	49
3	1	3	11	47.82609	8	12	18	15	53
2	0	1	7	30.43478	4	11	24	20	59
2	0	2	8	34.78261	7	8	18	16	49
2	4	3	18	78.26087	4	11	24	17	56
2	1	1	10	43.47826	11	12	18	16	57
2	1	3	11	47.82609	4	9	24	8	45
2	0	1	8	34.78261	10	5	21	13	49

1	2	2	11	47.82609	8	12	21	11	52
2	2	3	15	65.21739	9	4	23	17	53
2	1	0	9	39.13043	8	10	22	19	59
2	1	2	9	39.13043	4	9	24	10	47
2	2	2	11	47.82609	4	9	24	10	47
2	3	2	11	47.82609	4	10	21	14	49
3	3	3	18	78.26087	8	8	12	13	41
1	1	3	11	47.82609	5	8	24	18	55
1	3	2	12	52.17391	8	10	15	13	46
2	2	0	12	52.17391	4	7	20	15	46
3	0	1	12	52.17391	4	13	24	15	56
2	1	2	11	47.82609	7	7	23	16	53
1	1	0	7	30.43478	7	8	18	14	47
2	3	0	9	39.13043	8	9	15	12	44
1	3	2	10	43.47826	4	4	24	19	51
0	2	1	8	34.78261	7	12	21	13	53
1	1	1	11	47.82609	4	12	24	13	53
1	2	2	9	39.13043	9	5	10	11	35
0	1	1	9	39.13043	12	11	8	20	51
2	4	2	17	73.91304	7	11	24	17	59
1	3	2	11	47.82609	4	11	24	13	52
3	1	0	8	34.78261	11	11	18	16	56
2	1	3	10	43.47826	4	4	23	10	41
2	1	1	9	39.13043	7	14	23	12	56
0	2	2	8	34.78261	10	11	18	14	53
2	0	3	9	39.13043	5	10	22	15	52
1	2	0	7	30.43478	9	11	18	18	56
1	0	1	3	13.04348	4	12	17	18	51
1	1	2	9	39.13043	8	8	18	16	50
3	3	1	15	65.21739	6	16	18	15	55
3	1	1	10	43.47826	8	10	22	13	53
2	2	1	10	43.47826	8	10	24	14	56
1	1	2	10	43.47826	9	8	21	14	52
1	2	0	11	47.82609	7	9	10	15	41
1	1	1	8	34.78261	5	9	21	14	49
0	2	0	5	21.73913	10	10	20	19	59
3	2	2	11	47.82609	7	11	18	13	49
1	2	1	8	34.78261	5	14	15	10	44
1	2	2	8	34.78261	6	14	16	18	54
1	2	0	6	26.08696	10	11	18	16	55
1	2	1	8	34.78261	10	10	16	14	50
1	3	1	10	43.47826	4	12	20	15	51
2	2	2	9	39.13043	4	13	24	19	60
2	2	1	11	47.82609	10	10	20	18	58
2	3	1	11	47.82609	14	8	18	13	53
1	0	2	7	30.43478	9	8	18	12	47
1	0	1	7	30.43478	4	13	24	12	53
2	2	3	13	56.52174	8	11	20	13	52
2	1	1	10	43.47826	6	9	20	16	51
0	3	1	12	52.17391	5	10	24	13	52

2	3	1	17	73.91304	7	11	19	16	53
2	2	1	10	43.47826	11	12	18	21	62
1	1	2	9	39.13043	11	10	19	17	57
3	4	1	17	73.91304	4	8	18	14	44
1	1	2	9	39.13043	8	12	24	12	56
2	1	1	8	34.78261	8	10	19	13	50
2	0	1	12	52.17391	4	8	24	6	42
3	4	3	19	82.6087	4	11	19	12	46
2	2	1	12	52.17391	7	10	17	13	47
1	1	2	9	39.13043	8	6	24	11	49
3	0	2	10	43.47826	8	10	18	13	49
3	1	2	14	60.86957	8	7	18	13	46
1	2	1	11	47.82609	12	10	20	17	59
1	1	1	8	34.78261	6	12	13	16	47
1	2	1	11	47.82609	7	11	18	14	50
1	2	0	8	34.78261	8	15	24	21	68
1	0	3	9	39.13043	4	11	21	13	49
1	1	2	11	47.82609	4	10	23	12	49
1	1	2	12	52.17391	4	11	21	14	50
2	0	3	13	56.52174	9	12	18	16	55
2	1	0	7	30.43478	7	14	22	16	59
0	3	1	7	30.43478	8	9	18	18	53
1	2	2	9	39.13043	6	11	20	14	51
1	2	2	12	52.17391	9	12	24	15	60
2	0	2	8	34.78261	10	10	18	16	54
2	4	1	13	56.52174	5	8	18	14	45
3	1	2	14	60.86957	8	10	18	14	50
1	2	3	10	43.47826	9	9	16	13	47
3	2	2	15	65.21739	11	11	18	17	57
1	3	1	11	47.82609	14	13	19	17	63
3	3	0	10	43.47826	10	10	18	12	50
2	3	2	15	65.21739	8	12	18	14	52
2	3	2	14	60.86957	8	7	6	11	32
3	2	2	11	47.82609	9	10	18	12	49
2	1	0	11	47.82609	8	10	21	12	51
2	2	3	16	69.56522	8	11	18	15	52
1	1	1	11	47.82609	8	8	18	13	47
0	2	2	5	21.73913	12	13	15	7	47
2	2	1	11	47.82609	9	8	18	16	51
1	4	3	13	56.52174	8	10	18	12	48
3	1	1	10	43.47826	7	14	22	17	60
1	0	1	5	21.73913	6	10	23	14	53
1	1	2	11	47.82609	12	9	18	16	55
1	4	1	16	69.56522	6	11	22	13	52
3	4	3	19	82.6087	9	12	20	19	60
1	3	1	13	56.52174	8	12	18	14	52
1	2	3	12	52.17391	9	9	24	12	54
0	2	0	7	30.43478	10	13	21	20	64
2	5	1	19	82.6087	8	9	19	14	50
2	3	1	10	43.47826	4	6	10	13	33

1	4	3	14	60.86957	10	10	16	18	54
0	3	0	6	26.08696	13	10	15	14	52
2	3	2	14	60.86957	5	12	24	12	53
0	0	2	8	34.78261	4	16	9	16	45
1	5	0	11	47.82609	8	5	14	13	40
2	4	0	15	65.21739	9	11	18	13	51
2	2	0	8	34.78261	9	10	20	15	54
2	1	2	13	56.52174	7	11	18	15	51
2	1	2	12	52.17391	15	9	24	14	62
1	1	2	9	39.13043	4	8	24	16	52
0	2	1	8	34.78261	6	12	23	19	60
1	3	0	10	43.47826	7	11	21	14	53
1	3	3	13	56.52174	8	12	18	13	51
1	2	0	8	34.78261	9	12	13	12	46
2	0	1	6	26.08696	9	10	17	12	48
2	2	0	12	52.17391	5	9	21	16	51
1	3	2	10	43.47826	10	11	22	14	57
2	2	2	11	47.82609	11	4	18	10	43
3	3	2	15	65.21739	4	4	6	9	23
2	2	3	15	65.21739	8	11	24	12	55
2	1	1	9	39.13043	8	11	18	11	48
1	1	1	7	30.43478	12	8	12	15	47
0	1	0	7	30.43478	9	11	18	15	53
1	3	0	10	43.47826	9	9	18	14	50
1	0	0	5	21.73913	12	10	17	17	56
1	0	1	8	34.78261	6	10	20	15	51
3	0	2	11	47.82609	12	9	18	16	55
0	0	0	5	21.73913	8	11	18	14	51
0	1	0	7	30.43478	5	11	20	13	49
1	1	1	8	34.78261	8	11	18	14	51
0	2	0	8	34.78261	8	15	22	13	58
0	2	2	5	21.73913	8	11	23	18	60
2	1	0	9	39.13043	6	11	24	12	53
1	2	1	8	34.78261	4	13	18	15	50
2	1	3	12	52.17391	8	8	22	14	52
2	3	1	13	56.52174	6	9	18	10	43
1	0	2	6	26.08696	8	11	20	22	61
2	2	3	15	65.21739	9	11	22	16	58
2	0	0	7	30.43478	10	10	18	15	53
1	2	2	10	43.47826	4	9	24	16	53
1	4	2	14	60.86957	8	8	12	12	40
2	2	2	8	34.78261	12	12	24	18	66
1	2	0	7	30.43478	12	10	18	18	58
3	1	2	11	47.82609	9	8	17	13	47
0	2	1	9	39.13043	8	8	16	15	47
2	1	1	9	39.13043	8	12	20	12	52
2	1	1	7	30.43478	4	11	24	10	49
2	2	1	9	39.13043	4	8	18	13	43
2	1	2	10	43.47826	4	9	19	14	46
2	3	1	13	56.52174	9	13	24	16	62

1	1	2	13	56.52174	8	10	20	19	57
1	1	2	11	47.82609	11	8	18	14	51
2	1	0	9	39.13043	4	7	24	17	52
2	2	0	10	43.47826	9	13	18	15	55
2	2	0	8	34.78261	4	10	18	12	44
2	1	3	13	56.52174	4	13	24	18	59
2	3	1	11	47.82609	8	12	24	10	54
2	2	3	12	52.17391	7	7	24	10	48
0	2	2	10	43.47826	4	7	23	12	46
2	1	1	9	39.13043	11	10	14	16	51
2	2	0	9	39.13043	8	9	22	21	60
3	2	1	14	60.86957	12	12	18	17	59
1	2	1	9	39.13043	8	9	18	16	51
2	1	2	11	47.82609	10	10	18	13	51
2	3	0	11	47.82609	11	10	18	14	53
2	2	0	10	43.47826	10	10	18	15	53
2	3	2	13	56.52174	5	12	21	13	51
2	3	1	14	60.86957	8	8	23	17	56
1	0	1	8	34.78261	10	9	20	18	57
2	2	2	11	47.82609	6	14	24	10	54
2	1	2	10	43.47826	9	12	20	17	58
1	2	2	12	52.17391	9	12	18	15	54
1	2	2	12	52.17391	10	12	18	16	56
1	4	0	12	52.17391	9	13	18	15	55
1	3	2	12	52.17391	12	10	18	14	54
2	2	1	14	60.86957	11	12	22	14	59
2	3	0	13	56.52174	10	10	24	18	62
2	2	1	11	47.82609	8	9	21	19	57
2	2	1	12	52.17391	5	5	24	15	49
3	2	0	12	52.17391	13	9	14	11	47
2	3	3	16	69.56522	8	11	20	15	54
1	2	0	10	43.47826	6	4	24	17	51
2	3	0	11	47.82609	10	7	19	12	48
2	3	3	12	52.17391	5	9	14	11	39
3	2	0	11	47.82609	8	10	18	16	52
2	3	1	16	69.56522	6	11	23	17	57
2	3	2	15	65.21739	11	13	24	14	62
2	3	1	13	56.52174	11	12	20	13	56
2	1	1	12	52.17391	12	12	15	18	57

HBper	HR	Nutri	PA	HPB	HPBper	Threat	Expect	CenSus	CenSev
43.33333	12	24	19	55	46.37681	10	12	-3.89	-4.13
65	9	18	14	41	26.08696	17	6	-0.89	-0.13
63.33333	10	24	13	47	34.78261	19	9	0.11	0.87
58.33333	11	20	12	43	28.98551	22	1	2.11	1.87
60	14	16	17	47	34.78261	18	8	-0.89	0.87
66.66667	15	16	13	44	30.43478	24	0	4.11	1.87
53.33333	16	21	17	54	44.92754	20	4	1.11	0.87
43.33333	17	24	21	62	56.52174	13	15	-3.89	-1.13
43.33333	11	16	19	46	33.33333	16	-6	0.11	-2.13
41.66667	8	16	15	39	23.18841	16	7	-1.89	-0.13
45	13	25	17	55	46.37681	12	11	-3.89	-2.13
50	14	19	15	48	36.23188	19	5	2.11	-1.13
35	16	16	14	46	33.33333	16	1	-0.89	-1.13
53.33333	16	18	14	48	36.23188	18	12	0.11	-0.13
51.66667	17	16	17	50	39.13043	19	-2	-0.89	1.87
55	11	19	15	45	31.88406	18	5	0.11	-0.13
51.66667	12	23	15	50	39.13043	13	10	-3.89	-1.13
51.66667	17	18	15	50	39.13043	13	10	-3.89	-1.13
56.66667	8	19	11	38	21.73913	19	1	1.11	-0.13
56.66667	20	19	16	55	46.37681	19	3	0.11	0.87
60	16	20	18	54	44.92754	18	10	0.11	-0.13
60	11	13	14	38	21.73913	20	6	4.11	-2.13
53.33333	14	17	13	44	30.43478	18	6	-0.89	0.87
63.33333	15	19	16	50	39.13043	17	7	-0.89	-0.13
61.66667	11	17	11	39	23.18841	23	2	4.11	0.87
46.66667	12	19	20	51	40.57971	15	13	-2.89	-0.13
60	17	16	14	47	34.78261	20	8	2.11	-0.13
50	16	27	17	60	53.62319	19	3	0.11	0.87
58.33333	15	18	14	47	34.78261	19	4	2.11	-1.13
51.66667	17	18	15	50	39.13043	17	4	-0.89	-0.13
55	8	17	18	43	28.98551	16	9	-0.89	-1.13
48.33333	23	29	21	73	72.46377	11	10	-0.89	-6.13
63.33333	12	23	11	46	33.33333	12	0	-3.89	-2.13
48.33333	17	26	15	58	50.72464	13	6	-1.89	-3.13
50	12	31	18	61	55.07246	15	9	-0.89	-2.13
46.66667	16	17	22	55	46.37681	15	15	0.11	-3.13
55	14	27	21	62	56.52174	21	14	1.11	1.87
65	8	13	9	30	10.14493	23	12	3.11	1.87
71.66667	8	17	12	37	20.28986	25	10	2.11	4.87
53.33333	17	21	14	52	42.02899	16	10	-1.89	-0.13
60	9	13	12	34	15.94203	21	3	2.11	0.87
56.66667	11	18	13	42	27.53623	18	4	-1.89	1.87
71.66667	10	19	9	38	21.73913	23	8	2.11	2.87
53.33333	14	17	13	44	30.43478	16	6	-2.89	0.87
66.66667	16	22	14	52	42.02899	19	3	-0.89	1.87
65	12	11	11	34	15.94203	19	8	2.11	-1.13
60	8	20	14	42	27.53623	16	8	-3.89	1.87
56.66667	11	19	10	40	24.63768	13	5	-3.89	-1.13
56.66667	15	25	12	52	42.02899	13	7	-3.89	-1.13

63.33333	9	19	16	44	30.43478	19	9	0.11	0.87
50	15	22	15	52	42.02899	15	9	0.11	-3.13
65	8	18	15	41	26.08696	16	5	-3.89	1.87
45	22	22	19	63	57.97101	17	6	-3.89	2.87
56.66667	15	18	15	48	36.23188	20	2	3.11	-1.13
48.33333	13	16	11	40	24.63768	15	6	-2.89	-0.13
58.33333	21	15	17	53	43.47826	20	11	0.11	1.87
61.66667	18	19	21	58	50.72464	20	11	0.11	1.87
60	16	17	17	50	39.13043	22	12	2.11	1.87
53.33333	15	15	19	49	37.68116	17	11	-0.89	-0.13
51.66667	16	21	21	58	50.72464	20	9	2.11	-0.13
53.33333	17	20	15	52	42.02899	20	4	0.11	1.87
53.33333	16	18	19	53	43.47826	17	13	-1.89	0.87
53.33333	13	16	24	53	43.47826	15	11	-3.89	0.87
53.33333	12	17	21	50	39.13043	17	9	1.11	-2.13
50	17	16	18	51	40.57971	17	3	0.11	-1.13
48.33333	10	14	11	35	17.3913	16	9	0.11	-2.13
35	13	18	17	48	36.23188	9	8	-3.89	-5.13
53.33333	13	17	13	43	28.98551	17	3	0.11	-1.13
66.66667	8	20	11	39	23.18841	17	5	-1.89	0.87
51.66667	18	25	18	61	55.07246	17	2	1.11	-2.13
50	17	22	17	56	47.82609	17	9	-0.89	-0.13
70	13	18	12	43	28.98551	21	7	3.11	-0.13
63.33333	13	18	15	46	33.33333	19	3	2.11	-1.13
55	16	20	18	54	44.92754	20	5	1.11	0.87
58.33333	10	12	9	31	11.5942	10	3	-1.89	-6.13
41.66667	16	20	14	50	39.13043	15	10	-2.89	-0.13
60	10	12	9	31	11.5942	20	6	2.11	-0.13
70	19	20	16	55	46.37681	24	10	0.11	5.87
56.66667	19	21	13	53	43.47826	17	9	0.11	-1.13
41.66667	14	23	17	54	44.92754	10	5	-3.89	-4.13
41.66667	17	27	21	65	60.86957	12	15	-2.89	-3.13
51.66667	20	32	19	71	69.56522	18	15	-3.89	3.87
50	12	15	7	34	15.94203	20	6	2.11	-0.13
60	16	15	12	43	28.98551	14	4	1.11	-5.13
66.66667	12	11	9	32	13.04348	22	8	2.11	1.87
56.66667	15	19	14	48	36.23188	18	10	1.11	-1.13
56.66667	11	15	13	39	23.18841	20	8	1.11	0.87
71.66667	16	20	18	54	44.92754	25	8	3.11	3.87
68.33333	13	16	15	44	30.43478	22	9	0.11	3.87
48.33333	14	17	15	46	33.33333	14	7	-2.89	-1.13
60	20	22	19	61	55.07246	22	8	3.11	0.87
50	14	17	17	48	36.23188	14	12	-2.89	-1.13
61.66667	11	17	13	41	26.08696	22	3	3.11	0.87
61.66667	12	16	12	40	24.63768	21	8	0.11	2.87
60	11	14	11	36	18.84058	17	9	0.11	-1.13
58.33333	12	17	17	46	33.33333	15	8	-0.89	-2.13
48.33333	14	17	14	45	31.88406	19	6	-0.89	1.87
53.33333	11	19	11	41	26.08696	15	11	-0.89	-2.13
53.33333	17	24	15	56	47.82609	15	11	-3.89	0.87

56.66667	11	19	15	45	31.88406	15	9	-3.89	0.87
75	10	16	12	38	21.73913	23	2	4.11	0.87
61.66667	13	19	13	45	31.88406	17	8	-1.89	0.87
61.66667	9	17	12	38	21.73913	18	9	-3.89	3.87
50	18	23	18	59	52.17391	15	13	-2.89	-0.13
73.33333	12	19	11	42	27.53623	22	6	1.11	2.87
53.33333	12	17	17	46	33.33333	14	10	-3.89	-0.13
51.66667	17	16	17	50	39.13043	18	9	0.11	-0.13
48.33333	12	24	13	49	37.68116	13	12	-3.89	-1.13
28.33333	15	20	22	57	49.27536	17	-8	-2.89	1.87
58.33333	17	18	18	53	43.47826	19	10	-0.89	1.87
53.33333	13	17	16	46	33.33333	16	12	-0.89	-1.13
51.66667	21	17	20	58	50.72464	14	1	-1.89	-2.13
51.66667	10	14	17	41	26.08696	23	-4	4.11	0.87
48.33333	15	17	13	45	31.88406	13	10	-0.89	-4.13
53.33333	17	19	16	52	42.02899	17	11	-3.89	2.87
53.33333	15	25	14	54	44.92754	18	14	-0.89	0.87
63.33333	13	17	14	44	30.43478	18	8	-0.89	0.87
56.66667	18	22	16	56	47.82609	18	2	2.11	-2.13
41.66667	20	23	19	62	56.52174	12	7	-2.89	-3.13
40	13	19	15	47	34.78261	16	12	-1.89	-0.13
46.66667	30	29	26	85	89.85507	12	6	-0.89	-5.13
66.66667	10	15	12	37	20.28986	20	8	1.11	0.87
65	14	19	14	47	34.78261	18	7	0.11	-0.13
60	12	18	12	42	27.53623	17	5	0.11	-1.13
30	9	17	15	41	26.08696	17	-5	-0.89	-0.13
56.66667	13	19	16	48	36.23188	17	11	1.11	-2.13
53.33333	16	19	14	49	37.68116	20	14	2.11	-0.13
45	10	18	14	42	27.53623	14	11	-3.89	-0.13
53.33333	15	19	11	45	31.88406	14	10	-1.89	-2.13
63.33333	12	19	15	46	33.33333	22	10	3.11	0.87
51.66667	16	18	16	50	39.13043	19	4	0.11	0.87
40	23	24	17	64	59.42029	19	1	2.11	-1.13
48.33333	14	18	11	43	28.98551	18	5	0.11	-0.13
56.66667	16	15	15	46	33.33333	15	9	1.11	-4.13
46.66667	15	19	19	53	43.47826	17	5	0.11	-1.13
65	12	16	12	40	24.63768	22	11	4.11	-0.13
53.33333	19	20	21	60	53.62319	16	12	1.11	-3.13
40	9	14	15	38	21.73913	14	6	-3.89	-0.13
46.66667	16	17	16	49	37.68116	17	5	1.11	-2.13
56.66667	20	18	15	53	43.47826	19	7	0.11	0.87
65	16	17	14	47	34.78261	21	10	0.11	2.87
71.66667	12	18	11	41	26.08696	23	8	4.11	0.87
60	19	16	14	49	37.68116	22	14	2.11	1.87
66.66667	10	13	9	32	13.04348	23	1	3.11	1.87
48.33333	13	18	11	42	27.53623	17	2	-0.89	-0.13
53.33333	15	17	13	45	31.88406	16	8	-1.89	-0.13
46.66667	14	20	17	51	40.57971	12	10	-3.89	-2.13
45	14	19	19	52	42.02899	16	5	0.11	-2.13
61.66667	16	16	14	46	33.33333	24	3	4.11	1.87

40	19	19	18	56	47.82609	17	1	1.11	-2.13
50	14	10	15	39	23.18841	14	2	-1.89	-2.13
50	27	29	21	77	78.26087	17	3	2.11	-3.13
51.66667	22	28	18	68	65.21739	17	8	2.11	-3.13
53.33333	22	28	19	69	66.66667	17	7	2.11	-3.13
40	22	25	25	72	71.01449	8	12	-3.89	-6.13
56.66667	18	20	9	47	34.78261	20	12	1.11	0.87
36.66667	16	17	14	47	34.78261	10	16	-3.89	-4.13
73.33333	11	17	7	35	17.3913	22	6	2.11	1.87
46.66667	23	27	19	69	66.66667	14	14	-3.89	-0.13
56.66667	12	15	17	44	30.43478	19	11	2.11	-1.13
50	22	24	22	68	65.21739	18	4	0.11	-0.13
58.33333	24	24	21	69	66.66667	18	9	0.11	-0.13
55	13	20	12	45	31.88406	13	6	-2.89	-2.13
40	8	15	15	38	21.73913	14	6	-2.89	-1.13
46.66667	18	17	17	52	42.02899	17	3	1.11	-2.13
66.66667	8	8	21	37	20.28986	21	9	1.11	1.87
65	16	19	12	47	34.78261	18	5	0.11	-0.13
46.66667	21	22	17	60	53.62319	13	5	1.11	-6.13
48.33333	16	19	12	47	34.78261	17	4	1.11	-2.13
46.66667	15	13	15	43	28.98551	17	1	0.11	-1.13
53.33333	11	24	19	54	44.92754	18	12	3.11	-3.13
45	18	21	20	59	52.17391	17	6	0.11	-1.13
18.33333	17	17	13	47	34.78261	9	2	-3.89	-5.13
53.33333	8	9	7	24	1.449275	15	5	0.11	-3.13
43.33333	19	19	17	55	46.37681	18	8	-0.89	0.87
35	8	19	15	42	27.53623	9	4	-2.89	-6.13
56.66667	11	24	14	49	37.68116	17	11	0.11	-1.13
55	13	19	17	49	37.68116	15	10	3.11	-6.13
46.66667	21	15	17	53	43.47826	18	6	-1.89	1.87
41.66667	15	20	18	53	43.47826	10	13	-1.89	-6.13
65	21	23	15	59	52.17391	18	7	-3.89	3.87
80	16	16	13	45	31.88406	24	4	0.11	5.87
60	14	16	13	43	28.98551	18	10	0.11	-0.13
55	18	18	13	49	37.68116	14	9	0.11	-4.13
60	15	17	13	45	31.88406	19	-1	2.11	-1.13
43.33333	16	17	12	45	31.88406	12	2	-3.89	-2.13
40	15	22	19	56	47.82609	12	16	-1.89	-4.13
55	14	25	17	56	47.82609	15	10	-1.89	-1.13
61.66667	16	16	16	48	36.23188	21	6	4.11	-1.13
41.66667	19	24	19	62	56.52174	13	16	-1.89	-3.13
60	10	21	11	42	27.53623	21	13	2.11	0.87
51.66667	18	22	14	54	44.92754	18	3	1.11	-1.13
73.33333	30	20	14	64	59.42029	26	8	5.11	2.87
60	19	17	16	52	42.02899	16	8	-2.89	0.87
55	10	16	13	39	23.18841	14	9	-3.89	-0.13
68.33333	18	19	15	52	42.02899	22	9	3.11	0.87
56.66667	21	19	13	53	43.47826	16	10	-3.89	1.87
55	12	16	14	42	27.53623	22	5	4.11	-0.13
48.33333	16	21	15	52	42.02899	15	2	-1.89	-1.13

61.66667	11	13	13	37	20.28986	21	4	2.11	0.87
65	19	21	11	51	40.57971	20	5	0.11	1.87
50	10	15	17	42	27.53623	14	12	-1.89	-2.13
51.66667	16	18	21	55	46.37681	16	7	3.11	-5.13
55	16	17	18	51	40.57971	16	7	0.11	-2.13
66.66667	17	19	13	49	37.68116	20	8	1.11	0.87
40	15	16	14	45	31.88406	13	5	-3.89	-1.13
60	18	16	12	46	33.33333	19	3	0.11	0.87
51.66667	14	16	12	42	27.53623	20	3	3.11	-1.13
51.66667	16	16	15	47	34.78261	18	3	1.11	-1.13
75	11	22	23	56	47.82609	22	5	0.11	3.87
50	20	25	19	64	59.42029	14	12	-3.89	-0.13
50	17	19	15	51	40.57971	20	6	1.11	0.87
68.33333	10	18	15	43	28.98551	24	9	2.11	3.87
63.33333	15	17	14	46	33.33333	17	7	-1.89	0.87
70	16	19	11	46	33.33333	18	2	-1.89	1.87
71.66667	9	10	8	27	5.797101	23	6	4.11	0.87
51.66667	21	25	18	64	59.42029	18	5	-0.89	0.87
55	23	19	17	59	52.17391	22	1	-0.89	4.87
56.66667	10	16	14	40	24.63768	22	4	3.11	0.87
35	12	19	12	43	28.98551	15	-2	0.11	-3.13
60	17	19	16	52	42.02899	19	9	2.11	-1.13
68.33333	18	19	11	48	36.23188	21	2	3.11	-0.13
56.66667	24	21	15	60	53.62319	15	9	-2.89	-0.13
58.33333	17	28	16	61	55.07246	19	10	0.11	0.87
48.33333	20	24	16	60	53.62319	20	3	1.11	0.87
51.66667	15	19	11	45	31.88406	14	9	-3.89	-0.13
48.33333	21	20	18	59	52.17391	17	2	-0.89	-0.13
48.33333	18	22	21	61	55.07246	19	6	3.11	-2.13
60	18	19	15	52	42.02899	21	-1	4.11	-1.13
61.66667	27	27	20	74	73.91304	22	-11	2.11	1.87
65	9	19	19	47	34.78261	19	6	-2.89	3.87
51.66667	15	16	12	43	28.98551	18	1	2.11	-2.13
66.66667	11	22	8	41	26.08696	21	5	1.11	1.87
46.66667	17	22	14	53	43.47826	19	7	0.11	0.87
55	10	13	11	34	15.94203	20	3	1.11	0.87
80	11	16	10	37	20.28986	32	0	8.11	5.87
60	17	17	14	48	36.23188	17	9	0.11	-1.13
53.33333	10	16	10	36	18.84058	18	2	-1.89	1.87
53.33333	13	28	16	57	49.27536	19	3	0.11	0.87
66.66667	15	19	14	48	36.23188	19	7	0.11	0.87
51.66667	18	23	19	60	53.62319	17	8	0.11	-1.13
53.33333	18	20	19	57	49.27536	15	-3	2.11	-5.13
55	14	17	12	43	28.98551	18	7	-0.89	0.87
55	17	22	14	53	43.47826	15	8	-3.89	0.87
41.66667	20	22	13	55	46.37681	12	15	-0.89	-5.13
11.66667	13	14	13	40	24.63768	13	-2	-0.89	-4.13
35	18	18	15	51	40.57971	16	1	-1.89	-0.13
31.66667	25	26	21	72	71.01449	16	3	0.11	-2.13
51.66667	13	17	11	41	26.08696	18	15	-3.89	3.87

53.33333	10	15	15	40	24.63768	16	10	0.11	-2.13
53.33333	10	15	15	40	24.63768	16	10	0.11	-2.13
45	12	17	13	42	27.53623	14	3	-1.89	-2.13
53.33333	9	19	16	44	30.43478	17	5	0.11	-1.13
56.66667	16	17	12	45	31.88406	17	5	1.11	-2.13
56.66667	14	23	19	56	47.82609	16	6	-3.89	1.87
35	17	18	14	49	37.68116	11	-2	-2.89	-4.13
53.33333	15	24	18	57	49.27536	19	7	0.11	0.87
55	19	16	14	49	37.68116	17	0	0.11	-1.13
88.33333	21	19	14	54	44.92754	27	0	3.11	5.87
48.33333	11	16	15	42	27.53623	18	5	0.11	-0.13
51.66667	12	18	9	39	23.18841	17	-10	-1.89	0.87
21.66667	22	21	18	61	55.07246	11	6	-2.89	-4.13
63.33333	15	17	12	44	30.43478	19	7	0.11	0.87
56.66667	23	24	24	71	69.56522	16	8	-2.89	0.87
60	10	15	8	33	14.49275	17	7	1.11	-2.13
55	14	14	11	39	23.18841	18	5	0.11	-0.13
61.66667	10	21	18	49	37.68116	20	11	0.11	1.87
56.66667	17	17	15	49	37.68116	21	3	1.11	1.87
56.66667	19	25	17	61	55.07246	17	9	-1.89	0.87
53.33333	18	22	13	53	43.47826	14	4	-1.89	-2.13
66.66667	14	20	11	45	31.88406	21	-3	0.11	2.87
58.33333	9	17	21	47	34.78261	19	8	3.11	-2.13
51.66667	18	24	16	58	50.72464	14	9	-3.89	-0.13
53.33333	15	15	10	40	24.63768	19	3	0.11	0.87
66.66667	25	21	15	61	55.07246	18	6	-0.89	0.87
45	22	24	17	63	57.97101	16	-1	0.11	-2.13
46.66667	22	24	17	63	57.97101	16	-2	0.11	-2.13
58.33333	23	22	14	59	52.17391	16	9	-2.89	0.87
65	10	21	10	41	26.08696	19	8	0.11	0.87
51.66667	17	22	14	53	43.47826	15	10	-0.89	-2.13
51.66667	17	22	14	53	43.47826	15	10	-0.89	-2.13
75	17	19	15	51	40.57971	26	7	3.11	4.87
76.66667	11	16	14	41	26.08696	26	6	3.11	4.87
65	16	21	15	52	42.02899	17	6	-3.89	2.87
61.66667	11	18	11	40	24.63768	18	-1	-1.89	1.87
71.66667	15	18	14	47	34.78261	21	6	0.11	2.87
56.66667	25	25	21	71	69.56522	17	7	0.11	-1.13
61.66667	21	21	17	59	52.17391	21	0	2.11	0.87
60	18	21	16	55	46.37681	22	2	3.11	0.87
50	20	17	12	49	37.68116	17	9	-1.89	0.87
56.66667	20	21	23	64	59.42029	14	8	-0.89	-3.13
40	18	17	16	51	40.57971	21	-11	1.11	1.87
61.66667	16	18	14	48	36.23188	19	10	0.11	0.87
66.66667	15	16	12	43	28.98551	24	12	0.11	5.87
55	12	18	12	42	27.53623	18	7	0.11	-0.13
60	17	21	12	50	39.13043	21	11	1.11	1.87
78.33333	16	17	14	47	34.78261	23	4	2.11	2.87
60	17	23	12	52	42.02899	21	1	1.11	1.87
68.33333	16	17	14	47	34.78261	22	9	4.11	-0.13

48.33333	21	26	14	61	55.07246	20	11	2.11	-0.13
55	14	14	9	37	20.28986	19	6	0.11	0.87
55	18	17	14	49	37.68116	13	8	-3.89	-1.13
46.66667	15	16	12	43	28.98551	15	1	0.11	-3.13
65	15	19	12	46	33.33333	19	8	0.11	0.87
58.33333	14	20	12	46	33.33333	17	8	0.11	-1.13
21.66667	20	20	18	58	50.72464	9	12	-2.89	-6.13
68.33333	18	18	17	53	43.47826	19	6	3.11	-2.13
55	19	15	20	54	44.92754	20	13	3.11	-1.13
53.33333	11	20	17	48	36.23188	13	9	-3.89	-1.13
55	22	21	15	58	50.72464	18	5	1.11	-1.13
53.33333	19	17	13	49	37.68116	18	2	-0.89	0.87
53.33333	14	21	13	48	36.23188	16	10	-1.89	-0.13
63.33333	14	19	12	45	31.88406	18	8	1.11	-1.13
48.33333	13	20	12	45	31.88406	16	1	0.11	-2.13
56.66667	17	20	13	50	39.13043	19	5	0.11	0.87
60	17	18	14	49	37.68116	24	4	3.11	2.87
48.33333	22	19	14	55	46.37681	19	8	2.11	-1.13
61.66667	16	15	14	45	31.88406	17	6	-1.89	0.87
75	12	22	11	45	31.88406	22	5	0.11	3.87
46.66667	23	23	18	64	59.42029	17	3	0.11	-1.13
70	16	15	13	44	30.43478	21	7	2.11	0.87
53.33333	15	22	18	55	46.37681	17	13	0.11	-1.13
60	16	16	14	46	33.33333	17	1	0.11	-1.13
50	22	27	19	68	65.21739	17	9	0.11	-1.13
51.66667	15	26	16	57	49.27536	20	5	0.11	1.87
63.33333	17	24	17	58	50.72464	19	9	0.11	0.87
51.66667	17	22	12	51	40.57971	17	2	-1.89	0.87
73.33333	13	22	16	51	40.57971	24	8	2.11	3.87
55	15	17	10	42	27.53623	20	3	2.11	-0.13
61.66667	15	17	11	43	28.98551	19	6	0.11	0.87
56.66667	16	16	16	48	36.23188	20	8	0.11	1.87
58.33333	24	18	14	56	47.82609	15	8	-3.89	0.87
53.33333	12	16	11	39	23.18841	17	1	1.11	-2.13
58.33333	18	19	15	52	42.02899	18	7	2.11	-2.13
55	22	22	17	61	55.07246	19	12	1.11	-0.13
48.33333	15	22	10	47	34.78261	16	7	-2.89	0.87
76.66667	8	14	8	30	10.14493	21	3	-0.89	3.87
61.66667	19	21	14	54	44.92754	22	1	2.11	1.87
55	19	23	14	56	47.82609	15	10	0.11	-3.13
68.33333	18	16	14	48	36.23188	21	2	0.11	2.87
40	21	16	15	52	42.02899	14	4	-1.89	-2.13
51.66667	8	19	7	34	15.94203	18	3	1.11	-1.13
51.66667	8	19	7	34	15.94203	18	3	1.11	-1.13
63.33333	10	20	13	43	28.98551	17	7	-0.89	-0.13
60	21	22	15	58	50.72464	16	8	0.11	-2.13
51.66667	16	16	14	46	33.33333	20	5	0.11	1.87
58.33333	11	16	8	35	17.3913	18	11	-0.89	0.87
56.66667	18	20	14	52	42.02899	19	5	0.11	0.87
53.33333	15	17	14	46	33.33333	17	11	1.11	-2.13

58.33333	16	21	15	52	42.02899	20	1	1.11	0.87
71.66667	15	16	12	43	28.98551	23	8	4.11	0.87
45	23	26	25	74	73.91304	16	9	1.11	-3.13
50	19	23	14	56	47.82609	18	2	0.11	-0.13
63.33333	22	24	21	67	63.76812	21	-1	0.11	2.87
53.33333	21	28	23	72	71.01449	18	14	0.11	-0.13
46.66667	15	17	13	45	31.88406	18	6	0.11	-0.13
56.66667	21	20	18	59	52.17391	23	3	3.11	1.87
56.66667	16	15	14	45	31.88406	16	8	-2.89	0.87
61.66667	18	23	15	56	47.82609	20	7	4.11	-2.13
50	23	22	20	65	60.86957	17	15	-3.89	2.87
56.66667	13	14	15	42	27.53623	16	10	-2.89	0.87
43.33333	10	15	7	32	13.04348	15	7	0.11	-3.13
48.33333	19	22	15	56	47.82609	27	-10	3.11	5.87
63.33333	13	16	14	43	28.98551	21	7	-1.89	4.87
48.33333	14	14	12	40	24.63768	16	3	0.11	-2.13
63.33333	16	24	16	56	47.82609	19	9	0.11	0.87
51.66667	13	15	11	39	23.18841	19	4	0.11	0.87
71.66667	15	15	11	41	26.08696	26	3	2.11	5.87
43.33333	24	25	19	68	65.21739	8	10	-3.89	-6.13
48.33333	18	19	14	51	40.57971	17	16	-2.89	1.87
70	16	17	16	49	37.68116	22	8	0.11	3.87
65	11	16	12	39	23.18841	18	7	0.11	-0.13
53.33333	14	20	15	49	37.68116	19	15	0.11	0.87
56.66667	15	18	10	43	28.98551	20	2	0.11	1.87
56.66667	17	20	16	53	43.47826	15	9	-0.89	-2.13
41.66667	11	18	19	48	36.23188	14	7	-2.89	-1.13
58.33333	27	18	19	64	59.42029	21	12	1.11	1.87
40	16	19	15	50	39.13043	14	0	-1.89	-2.13
56.66667	16	17	13	46	33.33333	18	0	2.11	-2.13
56.66667	13	20	13	46	33.33333	21	9	2.11	0.87
48.33333	22	22	16	60	53.62319	14	13	-0.89	-3.13
48.33333	20	15	19	54	44.92754	18	-5	0.11	-0.13
68.33333	15	16	12	43	28.98551	22	1	-0.89	4.87
48.33333	18	20	14	52	42.02899	19	6	0.11	0.87
51.66667	14	23	12	49	37.68116	17	8	-1.89	0.87
55	14	23	12	49	37.68116	17	6	-1.89	0.87
56.66667	15	17	10	42	27.53623	19	1	0.11	0.87
60	15	20	16	51	40.57971	22	2	3.11	0.87
53.33333	24	24	21	69	66.66667	20	4	0.11	1.87
53.33333	21	15	18	54	44.92754	15	7	-0.89	-2.13
65	14	15	15	44	30.43478	21	6	-1.89	4.87
65	9	17	12	38	21.73913	21	6	-1.89	4.87
50	15	19	12	46	33.33333	20	6	0.11	1.87
51.66667	17	24	14	55	46.37681	13	10	-2.89	-2.13
66.66667	13	16	11	40	24.63768	18	6	0.11	-0.13
71.66667	18	16	11	45	31.88406	23	8	3.11	1.87
45	25	28	17	70	68.11594	12	11	-3.89	-2.13
46.66667	17	23	12	52	42.02899	15	11	-0.89	-2.13
66.66667	11	14	13	38	21.73913	20	6	-0.89	2.87

75	11	18	12	41	26.08696	22	5	1.11	2.87
51.66667	20	17	15	52	42.02899	15	0	-0.89	-2.13
60	16	18	14	48	36.23188	21	1	3.11	-0.13
66.66667	23	22	21	66	62.31884	19	1	-0.89	1.87
46.66667	24	24	21	69	66.66667	16	-2	0.11	-2.13
48.33333	19	17	18	54	44.92754	20	3	2.11	-0.13
50	16	17	14	47	34.78261	18	-2	1.11	-1.13
55	19	21	18	58	50.72464	21	4	1.11	1.87
56.66667	10	22	14	46	33.33333	16	8	0.11	-2.13
51.66667	18	19	15	52	42.02899	18	3	1.11	-1.13
53.33333	15	21	14	50	39.13043	14	8	-3.89	-0.13
66.66667	17	18	14	49	37.68116	19	3	0.11	0.87
51.66667	11	19	20	50	39.13043	17	12	-3.89	2.87
63.33333	17	17	14	48	36.23188	19	9	0.11	0.87
61.66667	13	16	13	42	27.53623	18	9	2.11	-2.13
45	10	17	19	46	33.33333	18	7	0.11	-0.13
46.66667	20	16	21	57	49.27536	17	17	0.11	-1.13
25	19	19	18	56	47.82609	11	0	-0.89	-6.13
53.33333	31	28	26	85	89.85507	14	0	-3.89	-0.13
60	31	28	26	85	89.85507	18	0	-3.89	3.87
51.66667	21	18	23	62	56.52174	17	12	-3.89	2.87
51.66667	16	16	14	46	33.33333	20	11	1.11	0.87
51.66667	16	16	14	46	33.33333	18	-1	1.11	-1.13
60	8	12	15	35	17.3913	14	6	-3.89	-0.13
80	24	26	23	73	72.46377	27	3	5.11	3.87
68.33333	8	16	12	36	18.84058	20	7	3.11	-1.13
50	12	18	14	44	30.43478	17	11	0.11	-1.13
71.66667	8	14	16	38	21.73913	21	4	0.11	2.87
55	10	16	13	39	23.18841	14	9	0.11	-4.13
41.66667	16	21	25	62	56.52174	16	17	-2.89	0.87
45	21	20	17	58	50.72464	17	4	0.11	-1.13
51.66667	15	23	15	53	43.47826	18	13	-0.89	0.87
41.66667	19	21	18	58	50.72464	10	-1	-1.89	-6.13
48.33333	18	18	15	51	40.57971	16	5	0.11	-2.13
55	15	18	13	46	33.33333	21	0	1.11	1.87
75	11	20	11	42	27.53623	26	7	3.11	4.87
33.33333	18	22	16	56	47.82609	18	-2	2.11	-2.13
46.66667	23	23	22	68	65.21739	14	2	-3.89	-0.13
78.33333	17	19	14	50	39.13043	25	-4	5.11	1.87
53.33333	21	18	16	55	46.37681	19	3	2.11	-1.13
46.66667	15	15	14	44	30.43478	9	-9	-2.89	-6.13
56.66667	21	23	18	62	56.52174	18	-2	2.11	-2.13
58.33333	21	20	23	64	59.42029	19	6	-0.89	1.87
58.33333	16	18	16	50	39.13043	20	1	2.11	-0.13
63.33333	16	19	12	47	34.78261	18	8	2.11	-2.13
48.33333	15	18	17	50	39.13043	13	8	-0.89	-4.13
65	18	22	15	55	46.37681	24	-3	5.11	0.87
55	18	19	18	55	46.37681	19	2	0.11	0.87
63.33333	15	19	17	51	40.57971	22	10	2.11	1.87
41.66667	13	21	13	47	34.78261	16	5	0.11	-2.13

50	19	17	15	51	40.57971	19	1	0.11	0.87
56.66667	10	19	14	43	28.98551	21	3	0.11	2.87
45	22	20	20	62	56.52174	13	14	0.11	-5.13
66.66667	12	17	13	42	27.53623	20	8	2.11	-0.13
51.66667	11	18	16	45	31.88406	19	0	-0.89	1.87
50	11	14	8	33	14.49275	18	4	2.11	-2.13
61.66667	16	15	13	44	30.43478	22	1	2.11	1.87
65	19	17	13	49	37.68116	24	7	4.11	1.87
61.66667	20	22	18	60	53.62319	25	4	4.11	2.87
78.33333	10	12	10	32	13.04348	27	2	5.11	3.87
56.66667	11	13	10	34	15.94203	17	7	2.11	-3.13
58.33333	15	12	11	38	21.73913	20	1	-0.89	2.87
56.66667	11	20	14	45	31.88406	17	9	-1.89	0.87
53.33333	17	25	14	56	47.82609	18	8	1.11	-1.13
61.66667	12	16	12	40	24.63768	16	7	-1.89	-0.13
46.66667	12	13	11	36	18.84058	19	1	0.11	0.87
70	9	14	12	35	17.3913	21	5	2.11	0.87
78.33333	16	17	14	47	34.78261	25	6	2.11	4.87
71.66667	9	16	11	36	18.84058	24	9	2.11	3.87
56.66667	9	14	12	35	17.3913	20	2	1.11	0.87
43.33333	21	24	17	62	56.52174	16	18	-3.89	1.87
60	11	15	18	44	30.43478	22	14	-0.89	4.87
18.33333	20	20	16	56	47.82609	11	0	-2.89	-4.13
53.33333	15	17	14	46	33.33333	15	11	-3.89	0.87
51.66667	14	17	16	47	34.78261	20	1	2.11	-0.13
65	22	24	20	66	62.31884	19	8	0.11	0.87
71.66667	15	15	14	44	30.43478	23	8	4.11	0.87
51.66667	8	15	15	38	21.73913	21	6	2.11	0.87
55	23	25	20	68	65.21739	18	7	0.11	-0.13
50	16	21	14	51	40.57971	15	5	-1.89	-1.13
55	12	21	15	48	36.23188	22	3	4.11	-0.13
65	16	16	14	46	33.33333	18	7	0.11	-0.13
63.33333	17	18	16	51	40.57971	22	0	4.11	-0.13
53.33333	16	15	14	45	31.88406	20	-4	0.11	1.87
65	16	22	14	52	42.02899	20	9	-2.89	4.87
86.66667	8	11	10	29	8.695652	29	3	5.11	5.87
53.33333	16	20	17	53	43.47826	19	3	0.11	0.87
50	16	16	15	47	34.78261	16	2	0.11	-2.13
75	15	15	15	45	31.88406	24	-1	3.11	2.87
51.66667	21	21	17	59	52.17391	21	0	4.11	-1.13
70	12	19	14	45	31.88406	22	8	1.11	2.87
51.66667	18	21	16	55	46.37681	18	3	0.11	-0.13
55	11	14	12	37	20.28986	18	11	2.11	-2.13
60	16	19	19	54	44.92754	21	9	2.11	0.87
50	22	25	23	70	68.11594	19	17	0.11	0.87
56.66667	11	17	16	44	30.43478	20	8	1.11	0.87
43.33333	27	26	24	77	78.26087	16	6	-3.89	1.87
45	18	20	12	50	39.13043	21	-4	4.11	-1.13
35	21	23	18	62	56.52174	18	-1	0.11	-0.13
53.33333	17	19	18	54	44.92754	20	4	2.11	-0.13

60	14	18	12	44	30.43478	22	8	4.11	-0.13
53.33333	18	25	16	59	52.17391	17	5	0.11	-1.13
48.33333	22	25	21	68	65.21739	20	13	0.11	1.87
45	18	18	20	56	47.82609	20	1	2.11	-0.13
50	19	18	19	56	47.82609	20	0	4.11	-2.13
61.66667	9	17	11	37	20.28986	20	7	2.11	-0.13
56.66667	14	16	12	42	27.53623	18	0	0.11	-0.13
53.33333	8	14	8	30	10.14493	18	2	1.11	-1.13
56.66667	11	16	14	41	26.08696	17	11	0.11	-1.13
45	21	18	21	60	53.62319	18	7	0.11	-0.13
51.66667	20	21	16	57	49.27536	20	1	2.11	-0.13
50	12	16	12	40	24.63768	20	0	0.11	1.87
68.33333	15	18	14	47	34.78261	22	9	0.11	3.87
63.33333	18	27	21	66	62.31884	24	2	2.11	3.87
53.33333	25	22	16	63	57.97101	19	13	0.11	0.87
46.66667	11	23	16	50	39.13043	17	-1	0.11	-1.13
43.33333	11	16	10	37	20.28986	11	1	-3.89	-3.13
48.33333	16	17	16	49	37.68116	16	5	0.11	-2.13
53.33333	21	18	17	56	47.82609	14	10	-3.89	-0.13
48.33333	17	17	16	50	39.13043	16	15	-1.89	-0.13
43.33333	11	17	11	39	23.18841	10	6	-3.89	-4.13
66.66667	12	19	20	51	40.57971	23	9	1.11	3.87
53.33333	17	17	14	48	36.23188	19	3	0.11	0.87
45	21	20	16	57	49.27536	18	-1	2.11	-2.13
58.33333	22	24	19	65	60.86957	21	4	3.11	-0.13
43.33333	18	22	15	55	46.37681	14	4	-1.89	-2.13
55	10	17	7	34	15.94203	19	2	0.11	0.87
51.66667	9	21	15	45	31.88406	20	5	0.11	1.87
50	15	17	10	42	27.53623	17	9	-3.89	2.87
46.66667	19	25	19	63	57.97101	21	-1	2.11	0.87
56.66667	13	15	15	43	28.98551	21	5	2.11	0.87
45	13	16	13	42	27.53623	17	6	1.11	-2.13
68.33333	13	14	11	38	21.73913	22	9	2.11	1.87
65	11	14	9	34	15.94203	25	2	4.11	2.87
65	11	15	8	34	15.94203	20	9	0.11	1.87
51.66667	10	16	12	38	21.73913	17	2	0.11	-1.13
61.66667	13	20	20	53	43.47826	21	4	3.11	-0.13
53.33333	20	24	17	61	55.07246	19	5	0.11	0.87
28.33333	27	19	22	68	65.21739	15	-4	0.11	-3.13
43.33333	25	13	16	54	44.92754	15	1	0.11	-3.13
65	8	18	14	40	24.63768	21	6	2.11	0.87
43.33333	16	16	15	47	34.78261	16	-2	0.11	-2.13
55	11	17	9	37	20.28986	16	3	-3.89	1.87
55	18	22	13	53	43.47826	18	3	-1.89	1.87
53.33333	16	16	14	46	33.33333	17	-5	0.11	-1.13
43.33333	23	19	19	61	55.07246	16	4	-2.89	0.87
50	10	11	12	33	14.49275	17	3	0.11	-1.13
63.33333	15	18	17	50	39.13043	22	10	0.11	3.87
63.33333	13	16	12	41	26.08696	13	3	-3.89	-1.13
40	13	13	14	40	24.63768	18	-2	0.11	-0.13

61.66667	26	23	19	68	65.21739	24	-1	2.11	3.87
56.66667	16	20	15	51	40.57971	18	4	2.11	-2.13
80	10	16	10	36	18.84058	23	3	4.11	0.87
61.66667	26	25	19	70	68.11594	17	2	0.11	-1.13
53.33333	18	20	16	54	44.92754	21	-3	2.11	0.87
60	11	13	11	35	17.3913	21	1	0.11	2.87
58.33333	21	19	21	61	55.07246	20	3	1.11	0.87
55	17	19	12	48	36.23188	22	5	2.11	1.87
60	19	18	12	49	37.68116	22	8	3.11	0.87
26.66667	14	18	18	50	39.13043	10	6	-1.89	-6.13
73.33333	20	21	17	58	50.72464	16	0	0.11	-2.13
60	16	17	13	46	33.33333	18	10	-1.89	1.87
66.66667	15	20	11	46	33.33333	20	6	0.11	1.87
51.66667	9	15	15	39	23.18841	17	6	1.11	-2.13
65	13	23	11	47	34.78261	19	8	-0.89	1.87
71.66667	9	18	14	41	26.08696	20	-5	3.11	-1.13
56.66667	16	15	13	44	30.43478	23	13	0.11	4.87
56.66667	13	15	15	43	28.98551	19	1	0.11	0.87
43.33333	16	16	16	48	36.23188	16	6	0.11	-2.13
70	14	17	16	47	34.78261	23	9	2.11	2.87
45	16	20	18	54	44.92754	18	7	-0.89	0.87
60	16	22	13	51	40.57971	17	9	0.11	-1.13
36.66667	31	27	24	82	85.50725	8	14	-3.89	-6.13
50	12	15	11	38	21.73913	16	10	-1.89	-0.13
45	16	17	17	50	39.13043	17	4	0.11	-1.13
38.33333	13	15	12	40	24.63768	20	-5	1.11	0.87
43.33333	21	23	19	63	57.97101	20	-2	0.11	1.87
58.33333	18	17	16	51	40.57971	20	-1	3.11	-1.13
48.33333	16	21	21	58	50.72464	16	11	-0.89	-1.13
93.33333	8	18	13	39	23.18841	28	0	4.11	5.87
60	9	14	9	32	13.04348	15	1	-3.89	0.87
45	16	21	17	54	44.92754	13	2	-3.89	-1.13
68.33333	17	20	13	50	39.13043	22	-3	0.11	3.87
61.66667	14	20	16	50	39.13043	22	9	3.11	0.87
55	19	24	17	60	53.62319	23	10	4.11	0.87
50	9	15	11	35	17.3913	18	6	0.11	-0.13
68.33333	15	20	9	44	30.43478	19	0	-1.89	2.87
46.66667	11	15	13	39	23.18841	17	5	0.11	-1.13
68.33333	16	22	16	54	44.92754	21	4	1.11	1.87
45	14	16	17	47	34.78261	18	3	1.11	-1.13
50	24	21	21	66	62.31884	14	12	-3.89	-0.13
51.66667	12	16	8	36	18.84058	16	1	1.11	-3.13
48.33333	12	12	11	35	17.3913	16	1	0.11	-2.13
55	14	18	14	46	33.33333	20	3	0.11	1.87
65	9	14	8	31	11.5942	15	4	-3.89	0.87
48.33333	23	20	20	63	57.97101	15	2	-0.89	-2.13
60	10	21	14	45	31.88406	15	7	-3.89	0.87
61.66667	13	13	12	38	21.73913	23	2	3.11	1.87
41.66667	13	22	17	52	42.02899	13	16	-3.89	-1.13
48.33333	15	26	15	56	47.82609	15	8	2.11	-5.13

53.33333	21	23	23	67	63.76812	20	10	0.11	1.87
55	11	19	19	49	37.68116	13	6	1.11	-6.13
65	15	17	13	45	31.88406	18	3	0.11	-0.13
45	17	16	15	48	36.23188	13	14	-3.89	-1.13
45	17	16	15	48	36.23188	13	14	-3.89	-1.13
48.33333	8	14	7	29	8.695652	14	7	-3.89	-0.13
35	19	15	15	49	37.68116	16	-1	0.11	-2.13
58.33333	16	19	10	45	31.88406	13	6	-2.89	-2.13
43.33333	22	23	19	64	59.42029	18	2	0.11	-0.13
43.33333	19	21	25	65	60.86957	11	5	-3.89	-3.13
60	23	24	18	65	60.86957	17	9	-3.89	2.87
55	11	21	17	49	37.68116	14	7	-0.89	-3.13
45	17	18	18	53	43.47826	15	4	-0.89	-2.13
40	19	20	17	56	47.82609	17	3	0.11	-1.13
51.66667	17	23	18	58	50.72464	8	5	-3.89	-6.13
55	18	16	24	58	50.72464	19	8	-0.89	1.87
55	12	29	15	56	47.82609	16	11	-3.89	1.87
25	29	24	21	74	73.91304	14	-1	1.11	-5.13
51.66667	8	21	8	37	20.28986	23	-12	4.11	0.87
65	12	22	10	44	30.43478	18	7	-0.89	0.87
53.33333	16	17	17	50	39.13043	15	11	-3.89	0.87
60	14	18	13	45	31.88406	22	2	3.11	0.87
35	10	14	12	36	18.84058	8	13	-3.89	-6.13
60	13	21	17	51	40.57971	21	11	-0.89	3.87
55	12	16	11	39	23.18841	21	4	2.11	0.87
53.33333	16	20	15	51	40.57971	15	7	-2.89	-0.13
60	17	17	14	48	36.23188	20	0	1.11	0.87
51.66667	14	16	11	41	26.08696	16	-1	-3.89	1.87
50	19	26	24	69	66.66667	16	2	0.11	-2.13
58.33333	12	17	11	40	24.63768	22	3	-1.89	5.87
55	20	21	21	62	56.52174	18	9	0.11	-0.13
60	15	16	13	44	30.43478	18	10	0.11	-0.13
53.33333	20	23	15	58	50.72464	17	7	1.11	-2.13
35	21	17	18	56	47.82609	16	-5	-0.89	-1.13
48.33333	14	18	14	46	33.33333	14	7	-2.89	-1.13
65	19	18	21	58	50.72464	20	1	2.11	-0.13
48.33333	15	16	15	46	33.33333	18	5	-0.89	0.87
40	22	21	19	62	56.52174	19	5	-2.89	3.87
56.66667	21	28	19	68	65.21739	20	-2	-1.89	3.87
58.33333	15	16	14	45	31.88406	21	2	2.11	0.87
50	19	22	17	58	50.72464	20	2	2.11	-0.13
51.66667	9	24	12	45	31.88406	16	5	-3.89	1.87
66.66667	21	22	10	53	43.47826	17	5	-3.89	2.87
63.33333	13	15	16	44	30.43478	20	2	2.11	-0.13
55	15	15	14	44	30.43478	22	5	6.11	-2.13
45	13	14	12	39	23.18841	17	6	1.11	-2.13
55	11	14	14	39	23.18841	17	12	-3.89	2.87
53.33333	19	14	16	49	37.68116	19	7	0.11	0.87
51.66667	12	18	17	47	34.78261	15	4	-1.89	-1.13
53.33333	19	21	14	54	44.92754	15	11	-2.89	-0.13

55	14	21	10	45	31.88406	18	3	-0.89	0.87
70	10	14	10	34	15.94203	23	-3	3.11	1.87
61.66667	9	13	8	30	10.14493	21	2	3.11	-0.13
40	16	19	11	46	33.33333	12	4	-3.89	-2.13
60	11	18	12	41	26.08696	20	12	0.11	1.87
50	16	16	15	47	34.78261	18	6	0.11	-0.13
36.66667	16	14	16	46	33.33333	12	18	-3.89	-2.13
43.33333	19	18	18	55	46.37681	15	7	-3.89	0.87
45	8	16	14	38	21.73913	17	4	-0.89	-0.13
48.33333	21	18	14	53	43.47826	14	13	0.11	-4.13
48.33333	16	15	12	43	28.98551	18	5	0.11	-0.13
43.33333	18	14	13	45	31.88406	15	5	0.11	-3.13
65	12	16	11	39	23.18841	22	3	4.11	-0.13
45	19	17	18	54	44.92754	18	-3	-1.89	1.87
50	16	22	12	50	39.13043	18	4	-0.89	0.87
80	8	10	10	28	7.246377	23	3	0.11	4.87
48.33333	14	15	15	44	30.43478	15	8	-3.89	0.87
48.33333	16	19	17	52	42.02899	14	11	-3.89	-0.13
50	18	18	18	54	44.92754	15	7	-3.89	0.87
58.33333	11	19	11	41	26.08696	21	2	1.11	1.87
65	15	22	24	61	55.07246	21	6	-0.89	3.87
55	20	17	19	56	47.82609	17	0	0.11	-1.13
51.66667	11	20	12	43	28.98551	17	6	-1.89	0.87
66.66667	19	18	13	50	39.13043	21	9	1.11	1.87
56.66667	13	20	15	48	36.23188	20	2	2.11	-0.13
41.66667	10	14	11	35	17.3913	13	4	-2.89	-2.13
50	14	15	11	40	24.63768	18	4	0.11	-0.13
45	10	18	9	37	20.28986	18	3	1.11	-1.13
61.66667	16	16	14	46	33.33333	22	1	3.11	0.87
71.66667	20	17	16	53	43.47826	27	2	6.11	2.87
50	15	15	16	46	33.33333	20	6	2.11	-0.13
53.33333	15	16	13	44	30.43478	20	4	0.11	1.87
20	15	24	18	57	49.27536	15	-5	0.11	-3.13
48.33333	12	17	13	42	27.53623	19	6	1.11	-0.13
51.66667	9	14	11	34	15.94203	18	9	0.11	-0.13
53.33333	15	16	14	45	31.88406	19	3	0.11	0.87
45	21	19	17	57	49.27536	16	5	0.11	-2.13
45	17	22	14	53	43.47826	25	8	4.11	2.87
51.66667	16	18	14	48	36.23188	17	2	1.11	-2.13
46.66667	18	21	18	57	49.27536	18	6	0.11	-0.13
66.66667	18	16	13	47	34.78261	21	5	-0.89	3.87
55	12	15	11	38	21.73913	16	9	-1.89	-0.13
58.33333	10	13	10	33	14.49275	21	2	4.11	-1.13
53.33333	13	27	17	57	49.27536	17	9	-1.89	0.87
66.66667	13	21	11	45	31.88406	21	1	1.11	1.87
53.33333	16	16	9	41	26.08696	20	4	0.11	1.87
56.66667	16	20	14	50	39.13043	18	12	1.11	-1.13
73.33333	18	15	15	48	36.23188	23	1	2.11	2.87
50	8	20	16	44	30.43478	17	5	0.11	-1.13
21.66667	21	19	13	53	43.47826	10	-3	-3.89	-4.13

56.66667	18	22	18	58	50.72464	20	-2	2.11	-0.13
53.33333	15	17	16	48	36.23188	23	1	5.11	-0.13
55	9	17	13	39	23.18841	17	12	-2.89	1.87
41.66667	24	25	22	71	69.56522	20	-7	-3.89	5.87
33.33333	18	22	14	54	44.92754	13	1	0.11	-5.13
51.66667	16	22	16	54	44.92754	20	5	1.11	0.87
56.66667	8	12	12	32	13.04348	19	5	1.11	-0.13
51.66667	9	15	9	33	14.49275	18	3	-0.89	0.87
70	12	19	9	40	24.63768	24	10	7.11	-1.13
53.33333	13	20	12	45	31.88406	12	8	-3.89	-2.13
66.66667	10	19	11	40	24.63768	18	4	-1.89	1.87
55	12	21	18	51	40.57971	18	7	-0.89	0.87
51.66667	24	24	21	69	66.66667	20	5	0.11	1.87
43.33333	20	16	17	53	43.47826	21	1	1.11	1.87
46.66667	16	16	14	46	33.33333	19	5	1.11	-0.13
51.66667	11	20	10	41	26.08696	14	5	-2.89	-1.13
61.66667	17	20	16	53	43.47826	21	8	2.11	0.87
38.33333	18	17	15	50	39.13043	15	8	3.11	-6.13
5	13	16	12	41	26.08696	8	-3	-3.89	-6.13
58.33333	22	18	16	56	47.82609	19	12	0.11	0.87
46.66667	24	24	21	69	66.66667	19	7	0.11	0.87
45	24	25	21	70	68.11594	20	-3	4.11	-2.13
55	15	19	14	48	36.23188	20	3	1.11	0.87
50	11	17	12	40	24.63768	18	4	1.11	-1.13
60	10	15	10	35	17.3913	22	0	4.11	-0.13
51.66667	9	17	9	35	17.3913	16	5	-1.89	-0.13
58.33333	12	15	12	39	23.18841	21	2	4.11	-1.13
51.66667	12	14	9	35	17.3913	19	4	0.11	0.87
48.33333	18	18	19	55	46.37681	16	7	-2.89	0.87
51.66667	10	15	9	34	15.94203	19	4	0.11	0.87
63.33333	12	18	10	40	24.63768	23	9	0.11	4.87
66.66667	17	21	14	52	42.02899	19	5	0.11	0.87
55	16	23	14	53	43.47826	17	12	-1.89	0.87
50	13	16	13	42	27.53623	17	3	-3.89	2.87
53.33333	27	27	16	70	68.11594	16	8	0.11	-2.13
38.33333	18	22	21	61	55.07246	15	8	-1.89	-1.13
68.33333	12	16	17	45	31.88406	19	-2	0.11	0.87
63.33333	13	17	12	42	27.53623	20	6	1.11	0.87
55	11	17	13	41	26.08696	20	3	2.11	-0.13
55	15	21	13	49	37.68116	13	8	-3.89	-1.13
33.33333	10	11	8	29	8.695652	16	0	0.11	-2.13
76.66667	18	23	17	58	50.72464	24	6	4.11	1.87
63.33333	13	16	12	41	26.08696	22	0	4.11	-0.13
45	16	19	17	52	42.02899	17	4	1.11	-2.13
45	18	20	18	56	47.82609	16	1	0.11	-2.13
53.33333	16	17	14	47	34.78261	20	8	0.11	1.87
48.33333	14	24	23	61	55.07246	15	14	-3.89	0.87
38.33333	13	19	11	43	28.98551	12	5	-3.89	-2.13
43.33333	16	21	12	49	37.68116	13	5	-3.89	-1.13
70	9	15	13	37	20.28986	22	8	1.11	2.87

61.66667	8	14	12	34	15.94203	18	1	0.11	-0.13
51.66667	13	18	12	43	28.98551	19	4	3.11	-2.13
53.33333	16	19	15	50	39.13043	11	7	-3.89	-3.13
58.33333	15	17	12	44	30.43478	22	3	1.11	2.87
40	14	13	11	38	21.73913	14	6	-3.89	-0.13
65	15	14	14	43	28.98551	17	6	-3.89	2.87
56.66667	15	14	10	39	23.18841	20	14	0.11	1.87
46.66667	20	20	14	54	44.92754	14	14	-0.89	-3.13
43.33333	14	19	14	47	34.78261	11	11	-3.89	-3.13
51.66667	13	17	19	49	37.68116	21	-2	3.11	-0.13
66.66667	9	12	10	31	11.5942	17	1	0.11	-1.13
65	13	17	13	43	28.98551	24	1	4.11	1.87
51.66667	16	19	16	51	40.57971	17	2	0.11	-1.13
51.66667	10	20	11	41	26.08696	20	5	2.11	-0.13
55	9	14	10	33	14.49275	21	4	3.11	-0.13
55	16	16	14	46	33.33333	20	3	2.11	-0.13
51.66667	17	18	15	50	39.13043	17	8	-2.89	1.87
60	15	17	12	44	30.43478	16	6	0.11	-2.13
61.66667	15	17	13	45	31.88406	19	2	2.11	-1.13
56.66667	21	26	19	66	62.31884	20	14	-1.89	3.87
63.33333	16	20	17	53	43.47826	21	3	1.11	1.87
56.66667	22	18	15	55	46.37681	21	3	1.11	1.87
60	9	15	10	34	15.94203	22	2	2.11	1.87
58.33333	11	14	13	38	21.73913	22	3	1.11	2.87
56.66667	14	21	17	52	42.02899	22	4	4.11	-0.13
65	10	17	13	40	24.63768	23	8	3.11	1.87
70	14	21	14	49	37.68116	20	6	2.11	-0.13
61.66667	18	12	10	40	24.63768	17	2	0.11	-1.13
48.33333	16	18	18	52	42.02899	10	9	-2.89	-5.13
45	19	16	19	54	44.92754	22	3	5.11	-1.13
56.66667	16	16	14	46	33.33333	19	5	0.11	0.87
51.66667	9	15	20	44	30.43478	10	7	-1.89	-6.13
46.66667	16	27	17	60	53.62319	17	7	2.11	-3.13
31.66667	17	18	17	52	42.02899	14	3	-2.89	-1.13
53.33333	15	18	13	46	33.33333	18	2	0.11	-0.13
61.66667	15	17	13	45	31.88406	17	6	-1.89	0.87
70	15	18	11	44	30.43478	24	10	3.11	2.87
60	14	19	17	50	39.13043	23	7	3.11	1.87
61.66667	18	22	22	62	56.52174	24	-3	4.11	1.87

CenBar	CenBen	CenIntent	Ceneffic
-2.61	3.82	1.59	0.56
3.39	3.82	-2.41	0.56
0.39	3.82	1.59	-0.44
1.39	-3.18	1.59	-0.44
0.39	2.82	-3.41	0.56
3.39	-2.18	-0.41	-0.44
-0.61	-2.18	-1.41	0.56
-5.61	3.82	-0.41	0.56
3.39	-8.18	1.59	-2.44
-3.61	-2.18	1.59	-0.44
-2.61	2.82	0.59	0.56
-1.61	-2.18	0.59	0.56
-2.61	-7.18	0.59	0.56
-3.61	2.82	-0.41	0.56
2.39	-5.18	-1.41	-1.44
0.39	-0.18	1.59	0.56
-0.61	3.82	1.59	0.56
-0.61	3.82	1.59	0.56
2.39	-2.18	-0.41	0.56
1.39	-1.18	-2.41	-1.44
-0.61	3.82	1.59	0.56
0.39	0.82	-0.41	-0.44
-0.61	-0.18	-1.41	0.56
2.39	3.82	-2.41	0.56
1.39	-2.18	-2.41	-0.44
-4.61	2.82	1.59	-0.44
-0.61	1.82	0.59	-0.44
-0.61	-3.18	-0.41	0.56
1.39	-0.18	1.59	-0.44
0.39	-1.18	-0.41	0.56
-0.61	2.82	1.59	-0.44
-0.61	3.82	1.59	0.56
8.39	2.82	1.59	0.56
0.39	0.82	1.59	0.56
-1.61	1.82	-0.41	0.56
-5.61	3.82	1.59	0.56
-5.61	2.82	-0.41	0.56
-2.61	3.82	1.59	0.56
-0.61	3.82	1.59	0.56
-1.61	2.82	0.59	-0.44
1.39	-1.18	-0.41	-0.44
1.39	-0.18	1.59	0.56
1.39	3.82	1.59	-0.44
0.39	0.82	0.59	0.56
4.39	1.82	-0.41	0.56
1.39	3.82	1.59	0.56
1.39	3.82	1.59	0.56
3.39	2.82	1.59	0.56
2.39	3.82	-2.41	0.56

0.39	3.82	1.59	0.56
-1.61	1.82	0.59	-0.44
4.39	3.82	1.59	-1.44
-2.61	-2.18	0.59	0.56
1.39	-2.18	-1.41	-0.44
-0.61	-0.18	-0.41	0.56
-2.61	2.82	-0.41	-1.44
-1.61	3.82	0.59	0.56
-3.61	2.82	-0.41	-0.44
-2.61	2.82	1.59	0.56
-3.61	-0.18	-0.41	0.56
-0.61	-2.18	-2.41	-0.44
-3.61	3.82	-0.41	0.56
-1.61	3.82	1.59	0.56
-1.61	1.82	1.59	-0.44
0.39	-2.18	0.59	-0.44
-2.61	0.82	-3.41	-0.44
-2.61	-0.18	0.59	0.56
1.39	-1.18	0.59	-0.44
4.39	3.82	1.59	-0.44
1.39	-2.18	-0.41	-0.44
-2.61	0.82	-0.41	-0.44
2.39	3.82	-0.41	0.56
3.39	0.82	-0.41	0.56
-0.61	-1.18	1.59	0.56
6.39	3.82	-2.41	-0.44
-4.61	-0.18	1.59	-0.44
0.39	0.82	-1.41	-0.44
-0.61	3.82	1.59	0.56
-0.61	2.82	1.59	0.56
0.39	-0.18	1.59	0.56
-5.61	3.82	0.59	0.56
-5.61	3.82	-1.41	0.56
-2.61	-2.18	-1.41	0.56
4.39	2.82	1.59	-0.44
0.39	2.82	1.59	-1.44
-1.61	2.82	-0.41	0.56
-1.61	0.82	1.59	0.56
0.39	2.82	1.59	0.56
0.39	3.82	-1.41	0.56
-0.61	0.82	-0.41	-0.44
-1.61	0.82	-2.41	-2.44
-2.61	3.82	0.59	-0.44
1.39	-1.18	1.59	0.56
-0.61	1.82	1.59	-0.44
0.39	3.82	1.59	-0.44
1.39	3.82	1.59	0.56
-2.61	-2.18	-0.41	0.56
-1.61	3.82	1.59	0.56
-1.61	3.82	-0.41	0.56

0.39	3.82	0.59	0.56
5.39	1.82	1.59	-0.44
1.39	3.82	1.59	0.56
0.39	3.82	1.59	0.56
-3.61	3.82	-0.41	0.56
3.39	3.82	1.59	0.56
-0.61	3.82	1.59	0.56
-2.61	0.82	1.59	0.56
-2.61	3.82	-2.41	0.56
-0.61	-14.18	1.59	0.56
-1.61	2.82	0.59	0.56
-2.61	3.82	1.59	-0.44
3.39	-1.18	-5.41	0.56
1.39	-8.18	1.59	0.56
-1.61	2.82	-0.41	0.56
-2.61	2.82	-0.41	0.56
-4.61	3.82	0.59	0.56
1.39	3.82	0.59	0.56
2.39	-1.18	0.59	-0.44
-1.61	-0.18	0.59	0.56
-6.61	-0.18	1.59	0.56
0.39	0.82	-0.41	-0.44
1.39	3.82	0.59	0.56
2.39	3.82	0.59	0.56
2.39	1.82	-0.41	-0.44
-1.61	-12.18	0.59	0.56
-1.61	3.82	0.59	0.56
-5.61	2.82	-0.41	-0.44
-3.61	1.82	1.59	0.56
-0.61	3.82	1.59	0.56
-1.61	2.82	1.59	0.56
-0.61	-2.18	-2.41	0.56
-2.61	-7.18	0.59	0.56
-1.61	-2.18	-0.41	-0.44
0.39	3.82	1.59	0.56
-1.61	-2.18	-0.41	0.56
-1.61	3.82	0.59	0.56
-2.61	3.82	1.59	-0.44
-2.61	-2.18	-1.41	-0.44
-1.61	-2.18	-0.41	0.56
-0.61	0.82	0.59	0.56
-0.61	3.82	1.59	0.56
1.39	3.82	-0.41	0.56
-4.61	3.82	0.59	0.56
3.39	-1.18	1.59	0.56
0.39	-3.18	-0.41	-0.44
-0.61	1.82	1.59	0.56
-1.61	2.82	-0.41	0.56
-1.61	-2.18	-0.41	0.56
0.39	-2.18	0.59	-1.44

-1.61	-6.18	-1.41	-1.44
2.39	-1.18	1.59	0.56
0.39	-2.18	1.59	0.56
-1.61	0.82	1.59	0.56
-0.61	0.82	1.59	0.56
-2.61	3.82	1.59	0.56
-3.61	2.82	0.59	0.56
-6.61	3.82	1.59	0.56
3.39	3.82	1.59	0.56
-4.61	3.82	-2.41	0.56
-2.61	2.82	-2.41	-0.44
-0.61	-2.18	-0.41	0.56
-0.61	2.82	-0.41	-0.44
2.39	2.82	0.59	0.56
-2.61	-2.18	1.59	-0.44
-0.61	-3.18	0.59	-0.44
0.39	3.82	-1.41	0.56
3.39	2.82	-0.41	0.56
0.39	-0.18	1.59	0.56
-0.61	-2.18	0.59	0.56
0.39	-4.18	1.59	0.56
-3.61	2.82	1.59	-0.44
-2.61	-2.18	-0.41	-0.44
-4.61	-8.18	-0.41	0.56
1.39	0.82	-0.41	-0.44
-4.61	-2.18	0.59	0.56
-0.61	-2.18	1.59	0.56
-1.61	3.82	1.59	0.56
-0.61	3.82	1.59	0.56
-2.61	-2.18	1.59	0.56
-3.61	3.82	1.59	0.56
2.39	3.82	1.59	0.56
5.39	3.82	0.59	-0.44
-0.61	3.82	0.59	0.56
0.39	3.82	1.59	0.56
4.39	-2.18	1.59	0.56
1.39	-2.18	0.59	0.56
-6.61	3.82	0.59	0.56
-0.61	3.82	-0.41	0.56
0.39	0.82	1.59	0.56
-6.61	3.82	0.59	0.56
-3.61	3.82	1.59	0.56
0.39	-2.18	-0.41	-0.44
0.39	2.82	0.59	-0.44
1.39	3.82	1.59	0.56
0.39	3.82	-0.41	0.56
0.39	3.82	-0.41	0.56
-0.61	3.82	1.59	0.56
-1.61	-2.18	-0.41	0.56
1.39	-2.18	-1.41	-0.44

1.39	-0.18	-1.41	0.56
2.39	1.82	-0.41	-0.44
-2.61	3.82	1.59	0.56
-0.61	0.82	0.59	0.56
0.39	1.82	1.59	0.56
1.39	3.82	1.59	-0.44
-1.61	-2.18	-2.41	-0.44
2.39	-0.18	1.59	0.56
-0.61	-3.18	-2.41	-0.44
0.39	-2.18	0.59	0.56
4.39	3.82	1.59	0.56
-2.61	3.82	1.59	0.56
-2.61	-2.18	-0.41	-0.44
-0.61	2.82	-2.41	0.56
2.39	3.82	0.59	0.56
6.39	2.82	1.59	0.56
2.39	2.82	0.59	-0.44
-0.61	-1.18	-1.41	0.56
0.39	-4.18	0.59	-0.44
-0.61	-2.18	1.59	-0.44
-0.61	-8.18	-0.41	-0.44
-0.61	2.82	-0.41	-0.44
4.39	0.82	0.59	-0.44
0.39	3.82	-2.41	0.56
-1.61	2.82	1.59	0.56
-1.61	-4.18	-1.41	-0.44
-0.61	2.82	-0.41	0.56
0.39	-3.18	-3.41	-1.44
-2.61	-2.18	-1.41	-0.44
3.39	-3.18	0.59	-0.44
8.39	-8.18	1.59	0.56
2.39	2.82	-1.41	0.56
1.39	-3.18	-2.41	-0.44
2.39	1.82	-1.41	0.56
-3.61	-2.18	-0.41	-0.44
0.39	-2.18	1.59	0.56
3.39	-2.18	1.59	-1.44
0.39	3.82	1.59	-2.44
1.39	-2.18	-1.41	-0.44
0.39	-2.18	1.59	0.56
2.39	3.82	-0.41	-0.44
-1.61	0.82	-0.41	0.56
5.39	-3.18	1.59	-0.44
-0.61	0.82	-1.41	-0.44
0.39	2.82	-1.41	0.56
-5.61	3.82	1.59	0.56
-6.61	-14.18	0.59	0.56
-2.61	-7.18	1.59	-2.44
-4.61	-7.18	1.59	0.56
-5.61	3.82	-2.41	-0.44

-1.61	2.82	-2.41	-0.44
-1.61	2.82	-2.41	-0.44
0.39	-2.18	-1.41	-0.44
0.39	-0.18	1.59	-0.44
1.39	0.82	1.59	0.56
1.39	1.82	1.59	0.56
1.39	-6.18	1.59	0.56
-1.61	-0.18	1.59	-0.44
3.39	-2.18	-1.41	0.56
8.39	2.82	-3.41	0.56
-1.61	-2.18	0.59	0.56
7.39	-8.18	1.59	-1.44
-6.61	-6.18	-0.41	0.56
1.39	2.82	-2.41	-0.44
0.39	2.82	-0.41	-2.44
1.39	2.82	-2.41	0.56
0.39	-0.18	1.59	-0.44
-1.61	3.82	1.59	-0.44
0.39	-2.18	-0.41	-0.44
-0.61	2.82	1.59	0.56
2.39	0.82	-0.41	0.56
6.39	-2.18	-0.41	-0.44
-0.61	1.82	0.59	-0.44
-0.61	2.82	1.59	0.56
0.39	-2.18	0.59	-0.44
3.39	3.82	0.59	-0.44
1.39	-5.18	-4.41	0.56
2.39	-5.18	-4.41	0.56
0.39	3.82	1.59	0.56
1.39	3.82	1.59	0.56
-1.61	2.82	-2.41	0.56
-1.61	2.82	-2.41	0.56
1.39	2.82	-0.41	0.56
2.39	2.82	1.59	0.56
3.39	3.82	-0.41	-2.44
5.39	-1.18	0.59	0.56
3.39	3.82	-1.41	-0.44
0.39	1.82	1.59	-0.44
3.39	-2.18	1.59	-0.44
1.39	-2.18	0.59	-0.44
-2.61	0.82	1.59	0.56
1.39	3.82	1.59	0.56
2.39	-14.18	1.59	-1.44
-0.61	3.82	-0.41	0.56
-2.61	3.82	-3.41	0.56
-0.61	0.82	0.59	0.56
-2.61	2.82	1.59	0.56
5.39	3.82	1.59	0.56
2.39	-2.18	-2.41	-0.44
0.39	3.82	-0.41	0.56

-5.61	-0.18	1.59	0.56
-0.61	-0.18	1.59	-0.44
1.39	3.82	0.59	0.56
1.39	-3.18	-0.41	-0.44
1.39	3.82	-2.41	0.56
0.39	2.82	0.59	0.56
-8.61	-2.18	0.59	-0.44
3.39	3.82	1.59	0.56
-4.61	2.82	0.59	0.56
0.39	3.82	1.59	-0.44
0.39	-0.18	0.59	0.56
1.39	-2.18	1.59	0.56
-1.61	2.82	1.59	-0.44
1.39	3.82	1.59	0.56
1.39	-3.18	1.59	-0.44
0.39	-0.18	0.59	0.56
-0.61	-2.18	0.59	-0.44
-3.61	-1.18	1.59	-0.44
2.39	2.82	-0.41	0.56
4.39	3.82	1.59	0.56
-0.61	-3.18	1.59	0.56
2.39	3.82	0.59	-0.44
-3.61	3.82	1.59	-0.44
4.39	-0.18	1.59	0.56
-2.61	0.82	-2.41	0.56
-1.61	-2.18	1.59	0.56
0.39	3.82	0.59	0.56
1.39	-2.18	1.59	-0.44
1.39	3.82	-0.41	0.56
0.39	-2.18	0.59	0.56
1.39	1.82	0.59	0.56
-1.61	0.82	1.59	-0.44
1.39	3.82	1.59	0.56
2.39	-2.18	-2.41	0.56
0.39	1.82	1.59	-0.44
-3.61	2.82	-2.41	0.56
-1.61	-0.18	-0.41	0.56
6.39	3.82	-0.41	-1.44
2.39	-2.18	-1.41	-0.44
-0.61	3.82	1.59	0.56
4.39	0.82	1.59	0.56
-1.61	-3.18	-1.41	-0.44
0.39	-2.18	-0.41	-1.44
0.39	-2.18	-0.41	-1.44
2.39	3.82	1.59	-0.44
1.39	3.82	0.59	0.56
-1.61	-2.18	-0.41	0.56
-1.61	3.82	1.59	0.56
0.39	-0.18	1.59	0.56
-2.61	2.82	-0.41	0.56

2.39	-2.18	1.59	0.56
1.39	3.82	1.59	0.56
-3.61	-0.18	0.59	0.56
0.39	-3.18	-1.41	-0.44
4.39	-2.18	-1.41	-0.44
-4.61	3.82	0.59	0.56
-2.61	-2.18	0.59	0.56
-0.61	-3.18	-0.41	0.56
0.39	2.82	1.59	-0.44
0.39	1.82	1.59	0.56
-5.61	3.82	1.59	0.56
-0.61	3.82	1.59	0.56
-2.61	-1.18	-0.41	-0.44
1.39	-14.18	1.59	0.56
0.39	1.82	1.59	0.56
0.39	-2.18	1.59	0.56
0.39	3.82	0.59	0.56
-0.61	-2.18	1.59	0.56
2.39	-0.18	1.59	0.56
-0.61	3.82	1.59	0.56
-6.61	3.82	1.59	0.56
1.39	3.82	1.59	0.56
2.39	3.82	1.59	0.56
-5.61	3.82	1.59	0.56
1.39	-2.18	1.59	-0.44
0.39	3.82	1.59	0.56
-2.61	-1.18	-0.41	0.56
-3.61	2.82	1.59	0.56
0.39	-5.18	1.59	0.56
3.39	-2.18	-0.41	-0.44
-2.61	0.82	0.59	-0.44
-3.61	3.82	0.59	0.56
3.39	-7.18	-2.41	-1.44
4.39	-0.18	1.59	0.56
-2.61	-2.18	-3.41	-0.44
-1.61	0.82	1.59	0.56
0.39	0.82	0.59	0.56
2.39	-2.18	1.59	0.56
1.39	-2.18	1.59	0.56
-0.61	-2.18	-2.41	-1.44
0.39	1.82	1.59	-0.44
1.39	1.82	1.59	0.56
1.39	1.82	1.59	0.56
-2.61	-2.18	-0.41	-0.44
-0.61	3.82	1.59	0.56
3.39	3.82	-0.41	0.56
1.39	3.82	1.59	0.56
-2.61	2.82	1.59	0.56
-3.61	1.82	1.59	0.56
2.39	2.82	1.59	-0.44

4.39	3.82	0.59	-1.44
3.39	-2.18	-2.41	-0.44
2.39	-2.18	-1.41	-0.44
5.39	0.82	-1.41	-1.44
2.39	-5.18	-2.41	-1.44
-1.61	-4.18	-0.41	-0.44
2.39	-5.18	1.59	-0.44
-0.61	-2.18	1.59	-0.44
0.39	2.82	-0.41	0.56
0.39	-2.18	-1.41	-0.44
0.39	2.82	0.59	-0.44
4.39	1.82	-1.41	0.56
-3.61	2.82	0.59	0.56
0.39	3.82	0.59	0.56
0.39	3.82	-2.41	-0.44
-3.61	-2.18	-0.41	0.56
-7.61	3.82	0.59	0.56
-2.61	-8.18	-6.41	0.56
4.39	-1.18	1.59	0.56
4.39	-1.18	1.59	0.56
-3.61	2.82	1.59	-0.44
-4.61	0.82	1.59	-0.44
2.39	-4.18	0.59	-0.44
3.39	3.82	1.59	-2.44
4.39	1.82	1.59	-2.44
2.39	3.82	1.59	0.56
-3.61	1.82	-1.41	0.56
4.39	2.82	-4.41	0.56
0.39	3.82	1.59	0.56
-8.61	2.82	-0.41	0.56
-1.61	-3.18	-0.41	0.56
-4.61	2.82	-1.41	-0.44
3.39	-3.18	-2.41	0.56
-0.61	-1.18	-0.41	-1.44
1.39	-4.18	-2.41	-1.44
1.39	2.82	0.59	0.56
-2.61	-10.18	-0.41	-1.44
1.39	-2.18	1.59	-0.44
8.39	-1.18	-0.41	-1.44
0.39	-2.18	-0.41	-0.44
9.39	-5.18	-2.41	0.56
4.39	-3.18	-2.41	-1.44
0.39	0.82	1.59	0.56
2.39	-2.18	-1.41	-1.44
1.39	3.82	1.59	0.56
-0.61	1.82	1.59	0.56
4.39	-4.18	-1.41	-1.44
1.39	-2.18	-5.41	-1.44
-1.61	2.82	1.59	0.56
-2.61	-3.18	1.59	0.56

0.39	-4.18	0.59	-0.44
0.39	-2.18	-0.41	-0.44
-4.61	3.82	0.59	0.56
1.39	3.82	1.59	0.56
1.39	-4.18	-0.41	0.56
-0.61	-2.18	-1.41	-0.44
2.39	-2.18	-0.41	0.56
-0.61	0.82	-0.41	-0.44
-0.61	-2.18	-1.41	-0.44
4.39	0.82	0.59	-0.44
0.39	1.82	-3.41	-0.44
2.39	-2.18	-1.41	-2.44
-0.61	2.82	-0.41	0.56
-1.61	0.82	1.59	0.56
2.39	3.82	-0.41	-0.44
-0.61	-5.18	-2.41	0.56
3.39	2.82	0.59	0.56
3.39	3.82	-2.41	-0.44
0.39	3.82	0.59	-0.44
1.39	-2.18	1.59	-0.44
-8.61	3.82	0.59	0.56
-4.61	3.82	1.59	0.56
-4.61	-10.18	1.59	-1.44
-1.61	3.82	1.59	0.56
0.39	-4.18	-2.41	-0.44
1.39	3.82	0.59	0.56
1.39	3.82	0.59	-0.44
-2.61	-2.18	-0.41	0.56
-0.61	0.82	1.59	0.56
0.39	-0.18	-1.41	0.56
-0.61	-3.18	0.59	-0.44
2.39	3.82	1.59	-0.44
3.39	-2.18	-0.41	0.56
3.39	-6.18	1.59	0.56
0.39	3.82	-0.41	0.56
5.39	2.82	-2.41	-0.44
0.39	-2.18	1.59	-0.44
1.39	-2.18	1.59	-0.44
6.39	-0.18	1.59	-0.44
0.39	-5.18	0.59	0.56
1.39	3.82	1.59	-0.44
0.39	-2.18	-0.41	0.56
-2.61	2.82	1.59	0.56
-1.61	1.82	0.59	-0.44
-7.61	3.82	0.59	0.56
-1.61	0.82	-0.41	-0.44
-2.61	-2.18	-1.41	-2.44
0.39	-9.18	-0.41	-0.44
-2.61	-9.18	-2.41	0.56
-0.61	-2.18	1.59	-1.44

-1.61	0.82	1.59	-0.44
0.39	-0.18	-1.41	0.56
-6.61	0.82	0.59	0.56
-1.61	-6.18	1.59	-1.44
0.39	-5.18	-1.41	-1.44
0.39	1.82	0.59	-0.44
3.39	-2.18	-2.41	-0.44
1.39	-2.18	-1.41	-0.44
-1.61	3.82	1.59	0.56
-3.61	-2.18	1.59	0.56
0.39	-4.18	0.59	-1.44
0.39	-5.18	-0.41	0.56
0.39	3.82	1.59	0.56
1.39	-2.18	0.59	0.56
-4.61	2.82	-0.41	0.56
1.39	-5.18	-0.41	0.56
2.39	-2.18	-2.41	-1.44
-0.61	-1.18	1.59	0.56
-0.61	3.82	1.59	0.56
-5.61	3.82	1.59	0.56
0.39	0.82	0.59	-0.44
-0.61	2.82	-0.41	-0.44
0.39	-2.18	-0.41	-0.44
0.39	-6.18	1.59	0.56
0.39	-1.18	-0.41	0.56
-0.61	-2.18	1.59	-0.44
1.39	-2.18	-3.41	-0.44
-1.61	-2.18	-2.41	0.56
-2.61	0.82	1.59	-1.44
-0.61	-7.18	-1.41	-0.44
-0.61	-1.18	0.59	-0.44
-2.61	-2.18	-2.41	-0.44
0.39	3.82	-1.41	-0.44
1.39	-2.18	-0.41	-0.44
0.39	3.82	-0.41	0.56
1.39	-2.18	-2.41	-0.44
1.39	-0.18	-2.41	-0.44
-0.61	-1.18	0.59	-0.44
-1.61	-11.18	-2.41	-0.44
0.39	-4.18	-1.41	0.56
1.39	1.82	-0.41	-0.44
1.39	-6.18	-1.41	-0.44
2.39	-0.18	-2.41	-0.44
1.39	-1.18	-2.41	-1.44
5.39	-5.18	1.59	-0.44
-1.61	-3.18	1.59	-1.44
0.39	-2.18	-2.41	-0.44
-1.61	2.82	1.59	0.56
6.39	3.82	0.59	-0.44
-0.61	-8.18	-2.41	-0.44

2.39	-4.18	-0.41	0.56
1.39	-0.18	-0.41	-0.44
6.39	3.82	-2.41	0.56
4.39	0.82	-1.41	-1.44
2.39	-6.18	-1.41	-0.44
2.39	-2.18	1.59	-1.44
1.39	-1.18	1.59	-0.44
-1.61	-2.18	0.59	0.56
-1.61	0.82	-0.41	0.56
-4.61	-4.18	-0.41	0.56
9.39	3.82	-2.41	-1.44
-0.61	3.82	-2.41	0.56
2.39	2.82	-2.41	-0.44
-0.61	-0.18	-2.41	-1.44
1.39	3.82	0.59	0.56
9.39	-1.18	-1.41	-1.44
-5.61	1.82	1.59	-0.44
2.39	-2.18	-2.41	-1.44
-2.61	-2.18	0.59	-0.44
0.39	3.82	-0.41	0.56
-3.61	-2.18	1.59	0.56
0.39	3.82	1.59	0.56
-4.61	3.82	1.59	0.56
-2.61	1.82	-3.41	-0.44
-1.61	-3.18	0.59	0.56
-0.61	-11.18	-1.41	-0.44
-0.61	-8.18	1.59	-0.44
3.39	-3.18	-2.41	-1.44
-3.61	1.82	1.59	0.56
9.39	3.82	1.59	-2.44
5.39	0.82	-0.41	0.56
1.39	-2.18	0.59	-0.44
6.39	-2.18	1.59	-2.44
-1.61	1.82	-0.41	0.56
-4.61	-0.18	-0.41	-0.44
-1.61	-1.18	1.59	0.56
6.39	0.82	-2.41	0.56
-1.61	-2.18	1.59	0.56
3.39	1.82	-0.41	0.56
-1.61	-4.18	-0.41	-2.44
-2.61	3.82	1.59	0.56
2.39	-2.18	0.59	-0.44
1.39	-3.18	1.59	0.56
0.39	-2.18	-2.41	-0.44
5.39	3.82	-3.41	0.56
1.39	-2.18	-1.41	0.56
2.39	3.82	1.59	-0.44
1.39	-2.18	0.59	-0.44
-6.61	3.82	-2.41	-0.44
-1.61	0.82	-0.41	0.56

-3.61	0.82	1.59	0.56
2.39	2.82	0.59	-0.44
4.39	1.82	-2.41	-0.44
-4.61	3.82	0.59	0.56
-4.61	3.82	-0.41	0.56
-0.61	0.82	1.59	0.56
-1.61	-8.18	-2.41	-0.44
3.39	3.82	-0.41	0.56
-1.61	-5.18	-0.41	-0.44
0.39	-0.18	1.59	0.56
0.39	3.82	-2.41	-0.44
1.39	2.82	-1.41	0.56
-0.61	-2.18	-1.41	-0.44
-2.61	-5.18	-0.41	-0.44
4.39	3.82	1.59	0.56
-1.61	0.82	0.59	0.56
-1.61	3.82	1.59	0.56
-3.61	-10.18	-2.41	-2.44
5.39	-12.18	1.59	0.56
2.39	3.82	-1.41	0.56
-1.61	3.82	1.59	0.56
1.39	-2.18	-2.41	0.56
-4.61	2.82	-2.41	0.56
-2.61	2.82	0.59	0.56
-0.61	-2.18	1.59	0.56
0.39	1.82	1.59	-0.44
3.39	-2.18	0.59	0.56
3.39	-3.18	1.59	-0.44
1.39	-2.18	1.59	0.56
0.39	-2.18	-1.41	-0.44
-1.61	1.82	-0.41	0.56
-0.61	3.82	-2.41	0.56
-0.61	0.82	1.59	0.56
0.39	-10.18	-2.41	-1.44
-0.61	0.82	-2.41	0.56
4.39	-0.18	1.59	-2.44
-1.61	-2.18	-0.41	-0.44
-4.61	-5.18	-1.41	0.56
3.39	-4.18	-1.41	-0.44
1.39	-2.18	-0.41	-0.44
-0.61	-4.18	-0.41	0.56
0.39	-0.18	0.59	-0.44
4.39	3.82	-0.41	0.56
3.39	-0.18	-0.41	-0.44
-1.61	-2.18	-0.41	-0.44
-2.61	-2.18	0.59	0.56
-2.61	3.82	-2.41	-0.44
-1.61	-0.18	-5.41	0.56
1.39	-0.18	0.59	-0.44
-1.61	3.82	-2.41	0.56

1.39	-1.18	-0.41	-0.44
6.39	-2.18	-0.41	-1.44
2.39	-1.18	-2.41	-0.44
-0.61	-2.18	0.59	0.56
-2.61	3.82	1.59	0.56
-1.61	-1.18	-1.41	0.56
-8.61	3.82	-0.41	0.56
-2.61	-1.18	0.59	0.56
-1.61	-3.18	-2.41	-0.44
-3.61	3.82	-1.41	0.56
-1.61	-2.18	-2.41	-0.44
-1.61	-2.18	-2.41	-0.44
2.39	-0.18	-0.41	-0.44
1.39	-7.18	-1.41	0.56
-0.61	-2.18	-0.41	0.56
6.39	3.82	-1.41	0.56
-1.61	0.82	0.59	-0.44
-2.61	2.82	-1.41	-0.44
-0.61	0.82	0.59	-0.44
1.39	-2.18	-0.41	-0.44
1.39	1.82	0.59	0.56
3.39	-2.18	-1.41	-0.44
-0.61	-0.18	0.59	0.56
0.39	3.82	1.59	-0.44
1.39	-2.18	-2.41	-0.44
-0.61	-2.18	-2.41	-0.44
-0.61	-2.18	-1.41	-0.44
-1.61	-4.18	-2.41	-0.44
2.39	-2.18	-0.41	0.56
2.39	-1.18	-0.41	-1.44
-2.61	-2.18	-0.41	-1.44
-0.61	-2.18	1.59	-0.44
-3.61	-14.18	0.59	-0.44
-2.61	-2.18	1.59	-0.44
-2.61	0.82	-0.41	0.56
0.39	-2.18	0.59	-0.44
-1.61	-2.18	-2.41	-0.44
-7.61	-5.18	-2.41	-1.44
1.39	-2.18	-2.41	-0.44
-2.61	-2.18	-2.41	-0.44
2.39	1.82	1.59	-0.44
-0.61	2.82	-1.41	-0.44
1.39	-2.18	-0.41	-0.44
-1.61	1.82	-0.41	0.56
4.39	-0.18	-0.41	-0.44
-0.61	-2.18	-0.41	-0.44
-2.61	3.82	0.59	0.56
5.39	0.82	1.59	0.56
-0.61	-1.18	-1.41	0.56
-1.61	-10.18	-2.41	0.56

3.39	-4.18	-0.41	-0.44
-0.61	-5.18	-0.41	0.56
-2.61	3.82	1.59	-0.44
1.39	-11.18	1.59	0.56
-1.61	-6.18	0.59	0.56
-1.61	-2.18	-0.41	-0.44
0.39	-0.18	0.59	-0.44
0.39	-2.18	0.59	0.56
-0.61	3.82	-1.41	-0.44
1.39	3.82	1.59	0.56
4.39	2.82	-0.41	-0.44
-0.61	0.82	-2.41	-0.44
-1.61	-2.18	-0.41	0.56
-2.61	-7.18	-0.41	0.56
-2.61	-3.18	-2.41	-0.44
1.39	0.82	0.59	-0.44
-0.61	1.82	-2.41	-0.44
-4.61	-2.18	-2.41	-0.44
-5.61	-14.18	1.59	-0.44
-2.61	3.82	-0.41	0.56
-3.61	-2.18	1.59	-0.44
0.39	-8.18	0.59	-0.44
0.39	-2.18	-2.41	-0.44
-0.61	-2.18	-1.41	-0.44
2.39	-3.18	-4.41	-0.44
0.39	-0.18	0.59	0.56
1.39	-2.18	0.59	0.56
-0.61	-2.18	-0.41	-0.44
-1.61	-0.18	-0.41	0.56
-0.61	-2.18	-1.41	-0.44
-1.61	1.82	-0.41	0.56
3.39	2.82	-0.41	0.56
-2.61	3.82	-0.41	0.56
0.39	-2.18	-0.41	-1.44
-0.61	1.82	1.59	0.56
-4.61	-2.18	-3.41	-0.44
7.39	-0.18	-0.41	0.56
1.39	1.82	-0.41	-0.44
0.39	-2.18	0.59	-0.44
1.39	3.82	-0.41	0.56
-2.61	-8.18	-2.41	-0.44
3.39	3.82	1.59	-0.44
3.39	-2.18	-0.41	0.56
-1.61	-3.18	0.59	-0.44
0.39	-4.18	-0.41	-0.44
-2.61	-0.18	-1.41	0.56
-4.61	3.82	1.59	0.56
-1.61	-2.18	0.59	0.56
-0.61	-1.18	1.59	-0.44
1.39	3.82	-0.41	0.56

4.39	-0.18	-6.41	0.56
-0.61	-2.18	-2.41	-1.44
2.39	3.82	0.59	0.56
0.39	-2.18	1.59	0.56
-2.61	-2.18	1.59	0.56
3.39	3.82	1.59	0.56
-4.61	3.82	-0.41	0.56
-4.61	3.82	-0.41	0.56
-2.61	2.82	-1.41	0.56
1.39	-6.18	-1.41	-0.44
6.39	1.82	-2.41	-0.44
2.39	-2.18	1.59	-0.44
1.39	-2.18	1.59	-0.44
-1.61	-2.18	-2.41	-0.44
-0.61	-2.18	-2.41	-1.44
0.39	-2.18	-2.41	-1.44
-1.61	0.82	-1.41	-0.44
2.39	2.82	1.59	-0.44
3.39	-0.18	-2.41	-0.44
-4.61	3.82	1.59	0.56
2.39	-0.18	1.59	-0.44
0.39	-2.18	1.59	-0.44
1.39	-2.18	-2.41	-0.44
0.39	-2.18	-2.41	-0.44
-0.61	-2.18	0.59	-0.44
-0.61	1.82	-0.41	-0.44
3.39	3.82	-0.41	0.56
4.39	0.82	-0.41	-0.44
0.39	3.82	1.59	0.56
-3.61	-6.18	-1.41	-0.44
0.39	-0.18	1.59	-0.44
2.39	3.82	1.59	0.56
-2.61	-1.18	-0.41	-0.44
-3.61	-6.18	-6.41	0.56
1.39	-2.18	-0.41	-0.44
2.39	2.82	1.59	0.56
-0.61	3.82	-0.41	-0.44
-1.61	-0.18	-1.41	-1.44
3.39	-5.18	-3.41	0.56
